# FAX1, a Novel Membrane Protein Mediating Plastid Fatty Acid Export

**DOI:** 10.1371/journal.pbio.1002053

**Published:** 2015-02-03

**Authors:** Nannan Li, Irene Luise Gügel, Patrick Giavalisco, Viktoria Zeisler, Lukas Schreiber, Jürgen Soll, Katrin Philippar

**Affiliations:** 1 Biochemie und Physiologie der Pflanzen, Department Biologie I - Botanik, Ludwig-Maximilians-Universität München, Planegg-Martinsried, Germany; 2 Research Center of Bioenergy and Bioremediation RCBB, College of Resources and Environment, Southwest University, Beibei Dist., Chongqing, P.R. China; 3 Munich Centre for Integrated Protein Science CIPSM, Ludwig-Maximilians-Universität München, München, Germany; 4 Max Planck Institut für Molekulare Pflanzenphysiologie MPIMP, Potsdam-Golm, Germany; 5 Institute of Cellular and Molecular Botany, Department of Ecophysiology, University of Bonn, Bonn, Germany; University of Massachusetts at Amherst, UNITED STATES

## Abstract

Fatty acid synthesis in plants occurs in plastids, and thus, export for subsequent acyl editing and lipid assembly in the cytosol and endoplasmatic reticulum is required. Yet, the transport mechanism for plastid fatty acids still remains enigmatic. We isolated FAX1 (*fatty acid export 1*), a novel protein, which inserts into the chloroplast inner envelope by α-helical membrane-spanning domains. Detailed phenotypic and ultrastructural analyses of FAX1 mutants in *Arabidopsis thaliana* showed that FAX1 function is crucial for biomass production, male fertility and synthesis of fatty acid-derived compounds such as lipids, ketone waxes, or pollen cell wall material. Determination of lipid, fatty acid, and wax contents by mass spectrometry revealed that endoplasmatic reticulum (ER)-derived lipids decreased when FAX1 was missing, but levels of several plastid-produced species increased. FAX1 over-expressing lines showed the opposite behavior, including a pronounced increase of triacyglycerol oils in flowers and leaves. Furthermore, the cuticular layer of stems from *fax1* knockout lines was specifically reduced in C29 ketone wax compounds. Differential gene expression in FAX1 mutants as determined by DNA microarray analysis confirmed phenotypes and metabolic imbalances. Since in yeast FAX1 could complement for fatty acid transport, we concluded that FAX1 mediates fatty acid export from plastids. In vertebrates, FAX1 relatives are structurally related, mitochondrial membrane proteins of so-far unknown function. Therefore, this protein family might represent a powerful tool not only to increase lipid/biofuel production in plants but also to explore novel transport systems involved in vertebrate fatty acid and lipid metabolism.

## Abstract 2

The novel protein FAX1 mediates the export of free fatty acids across the inner membrane of chloroplasts so that they can be processed in other plant cell organelles to generate oils, waxes, and other lipids.

## Author Summary

Fatty acid synthesis in plants occurs in chloroplasts—the organelle more commonly known for conducting photosynthesis. For subsequent lipid assembly to be possible in the endoplasmatic reticulum (ER), export of these fatty acids across the chloroplast envelope membranes is required. The mechanism of this transport until now has not been known. We isolated FAX1 (*fatty acid export 1*), a novel membrane protein in chloroplast inner envelopes. FAX1 function is crucial for biomass production, male fertility, and the synthesis of fatty acid-derived compounds like lipids, waxes, or cell wall material of pollen grains. Whereas ER-derived lipids decreased when FAX1 was missing, levels of plastid-produced lipids increased. FAX1 over-expressing mutants showed the opposite behavior, including an increase of triacyglycerol oils. Because FAX1 could complement for fatty acid transport in yeast, we concluded that FAX1 mediates the export of free fatty acids from chloroplasts. In vertebrates, FAX1 relatives are structurally related proteins of so-far unknown function in mitochondria. This protein family may thus represent a powerful tool not only to increase lipid oil and biofuel production in plants but also to explore novel transport systems in animals.

## Introduction

Fatty acids (FAs) are building blocks for the majority of cellular lipids, which are essential throughout life of organisms. Besides their role as constituents of biological membranes, plant acyl-lipids are used for diverse functions at different destinations and tissues (reviewed in [1]). For example, triacylglycerols (TAGs) in seeds of oilseed plants represent the major form of carbon and energy storage. Cuticular waxes at the surface of plants restrict loss of water and provide protection against pathogen attack. Furthermore, the formation of pollen cell walls is strictly dependent on delivery of modified FAs from tapetum cells in anthers (reviewed in [2]). De novo FA synthesis in plants occurs in plastids (for overview, see [1,3]). Growing alkyl chains in the plastid stroma are attached as acyl moieties to acyl carrier protein (ACP), and in seed plants become available for lipid assembly mainly in the form of palmitoyl (16:0)- and oleoyl (18:1)-ACP. Part of these long-chain FAs will be integrated into lipids inside plastids (prokaryotic pathway); the majority, however, is exported to the endoplasmic reticulum (ER) for further elongation, acyl editing, and lipid synthesis (eukaryotic pathway).

Although it is generally agreed that free FAs are shuttled across plastid envelope membranes, the mode of export still remains enigmatic [4] since until now, no membrane-intrinsic transporter protein could be associated with a direct function in plastid FA export (for overview, see [1,3]). On the one hand, a facilitated diffusion of free FAs through the lipid environment of membranes is suggested, which is supported by the recent finding that an acyl-ACP synthase in the cyanobacterium *Synechocystis* sp. PCC6803 is necessary and sufficient for FA transfer across membranes [5]. On the other hand, several ATP-binding cassette (ABC) transporter proteins for lipids, FAs, or acyl-coenzyme A (CoA), and for import of FAs into peroxisomes [6], as well as FA-transport systems from *Escherichia coli*, yeast, or mammals, provide evidence for an active mode of transport in plastids. Nevertheless, before transport, acyl-ACP thioesterases at the inner plastid envelope membrane catalyze the hydrolysation of fatty acyl-ACP to free FAs. After crossing both inner and outer plastid envelope membranes (IE, OE), free FAs are re-activated to acyl-CoAs by long-chain acyl-CoA synthetases (LACSs). As demonstrated for the protein LACS9, these enzymes can attach to the cytosolic face of the plastid OE [7–9]. At the ER membrane, the ABC transporter ABCA9 has recently been described to be involved in FA-uptake, most likely in the form of acyl-CoA, thereby being important for TAG synthesis during seed filling [10]. Once arrived in the ER lumen, plastid-derived FAs are utilized for synthesis of specific lipid classes via the so-called eukaryotic pathway, where phosphatidic acid (PA) represents an important intermediate, phosphatidyl-choline (PC) is a major membrane phospholipid, and TAGs are the energy storage lipids produced. Subsequently, these eukaryotic lipids are distributed to various subcellular locations. For re-import of eukaryotic lipids into plastids, most likely in the form of ER-derived PA, an ABC transporter system (TGD1, 2, 3) at the IE [3] and the PA-binding ß-barrel lipid transfer protein TGD4 in the OE [11] are required. In plastids, the diacylglycerol backbone from these eukaryotic precursors is used for synthesis of the galactolipids MGDG, DGDG (monogalactosyl-, digalactosyl-diacylglycerol), and the sulfolipid SQDG (sulfoquinovosyl-diacylglycerol). In addition, however, a prokaryotic-type pathway also produces MGDG, DGDG, SQDG, and the phospholipid phosphatidyl-glycerol (PG) directly from newly synthesized FAs and thus does not require previous FA-export from plastids (for overview, see [1]).

Here we describe FAX1, a novel protein in the IE of plastids that belongs to the Tmemb_14 superfamily of membrane proteins with so-far unknown function. Functional studies in yeast as well as *FAX1* mutant analysis in *Arabidopsis thaliana* clearly demonstrate that FAX1 mediates FA-export from plastids and thus, to our knowledge, represents the first membrane-intrinsic protein described to be involved in this process. In mammals, FAX1 relatives are structurally related mitochondrial membrane proteins, for which the biological task is not yet clear [12–14]. Thus, FAX1 not only is a missing link to explain the mode of plastid FA-export and to improve plant lipid/biofuel production but might also propel the understanding of Tmemb_14 protein performance in general.

## Results

### FAX1, a Novel Chloroplast Inner Envelope Membrane Protein

**Fig 1:**
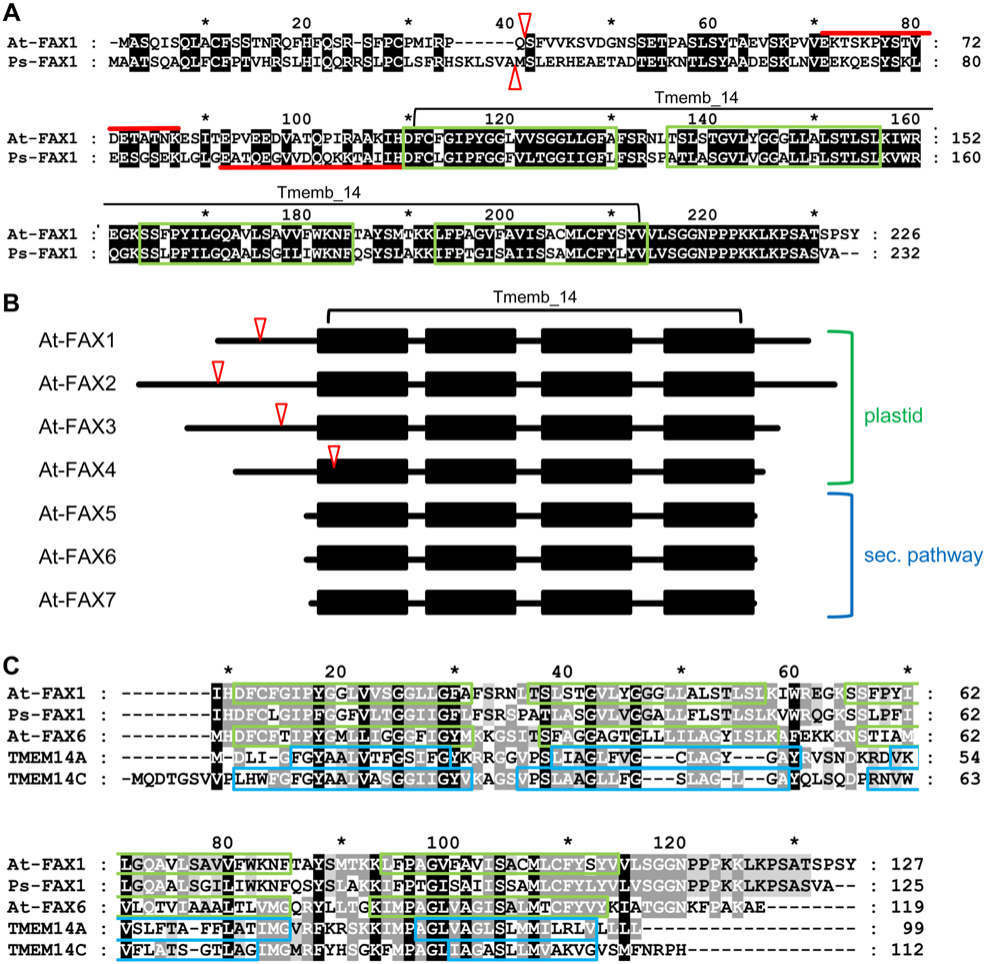
Plant FAX and human Tmemb_14 proteins. (A) Arabidopsis At-FAX1 (At3g57280, 226 amino acids [aa]) is accessible at the ARAMEMNON database [18], the sequence for pea Ps-FAX1 (232 aa) was deposited at National Center for Biotechnology Information (NCBI), GenBank acc. no. KF981436. Predicted chloroplast targeting peptides (ChloroP; http://www.cbs.dtu.dk/services/ChloroP), with 33 aa and 39 aa for At-FAX1 and Ps-FAX1, respectively, are marked with red triangles. The Tmemb_14 domain (Pfam|PF03647) of At-FAX1, including the four conserved hydrophobic domains, is indicated. Identical amino acids (49%) are shaded in black, hydrophobic α-helices (ARAMEMNON) are boxed in green, and peptides used for generation of antisera are indicated by red lines. (B) Members of the FAX/Tmemb_14 family in Arabidopsis. Whereas At-FAX1-At-FAX4 are predicted to be in plastids, At-FAX5-At-FAX7 most likely localize to membranes of the secretory pathway. Hydrophobic α-helices (black squares) and subcellular localization are depicted according to ARAMEMNON. Predicted chloroplast targeting peptides (ChloroP) are marked with red triangles. (C) At-FAX1 and Ps-FAX1 [sequence starting with Tmemb_14 domain, see (A)] in comparison to At-FAX6 (At3g20510) and human proteins of the Tmemb_14 superfamily: TMEM14A (Q9Y6G1) and TMEM14C (Q9POS9). Arabidopsis genome initiative (AGI) codes and InterPro accession numbers in brackets. Whereas At-FAX1 and Ps-FAX1 are slightly more similar to TMEM14C (17% identical, 35% similar aa), At-FAX6 shares 28% identical aa with both proteins TMEM14A and 14C. Predicted hydrophobic α-helices (ARAMEMNON) are boxed in green; α-helices in TMEM14A, 14C [14] are boxed in blue.

The Arabidopsis protein At-FAX1 (At3g57280, for *fatty acid export 1*) was previously annotated as potential plastid-targeted and plant-specific solute transporter by proteomic and phylogenetic analysis [15,16]. Furthermore, we identified transcripts of *At-FAX1* to be up-regulated upon induction of early leaf senescence [17]. To analyze protein function, we isolated the cDNA of *FAX1* genes from Arabidopsis and pea (*Pisum sativum*). For both proteins, chloroplast targeting peptides and four hydrophobic α-helices are predicted (Fig. 1A). By the latter, plant FAX1 clearly groups to the so-called Tmemb_14 superfamily of proteins with so-far unknown function. The Tmemb_14 family is ubiquitous, with members in nearly all eukaryotes and some bacteria (InterPro|UPF0136). In Arabidopsis, four proteins (FAX1–FAX4) are predicted to be targeted to plastids, while three (FAX5–FAX7) most likely are directed to other, non-plastid membranes via the secretory pathway (Fig. 1B). The plastid-intrinsic FAX1 is restricted to the chlorophyll-containing plant kingdom, with representatives in mono- and dicotyledons as well as in mosses and green algae (compare InterPro|UPF0136, [15]). Relatives of non-plastid predicted At-FAX proteins, however, can be found in eukaryotes such as mammals, insects, or yeast, and in some bacteria and cyanobacteria (e.g., *Chlamydiae* or *Nostocales*).

**Fig 2:**
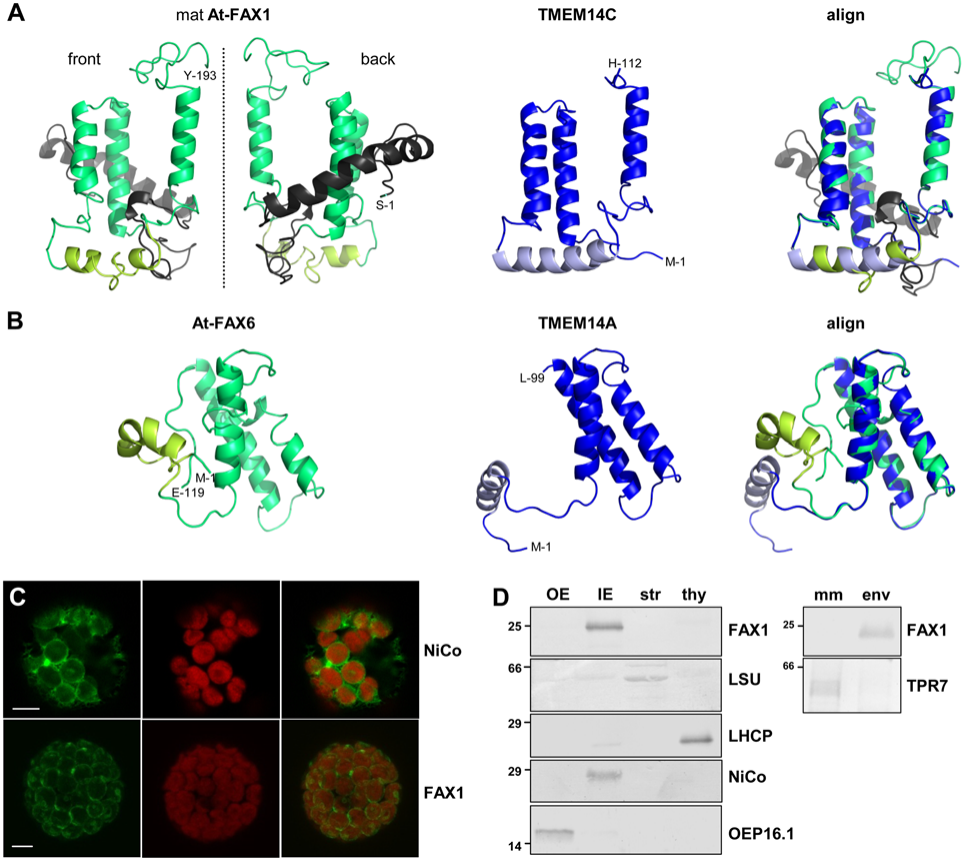
FAX1, a chloroplast IE protein of the Tmemb_14 family. (A, B) Structural models and alignment (right) of the mature At-FAX1 / human TMEM14C (A) and of At-FAX6 / human TMEM14A (B) proteins. Membrane-spanning and amphiphilic α-helices of FAX and of TMEM14 are depicted in green/yellow-green and blue/light blue, respectively. Please note that At-FAX1 contains an additional N-terminal stretch that most likely folds into another α-helix (gray). First and last amino acid residues are indicated. (C) In vivo green fluorescent protein (GFP)-targeting. Arabidopsis leaf protoplasts were transiently transformed with constructs for FAX1- and NiCo-GFP (chloroplast IE marker; [19]). Images show GFP and chlorophyll fluorescence, as well as an overlay of both (bar = 5 μm). (D) Immunoblot analysis of FAX1 in chloroplast subfractions. Equal protein amounts (5μg) of pea chloroplast outer envelope (OE), inner envelope (IE), stroma (str), thylakoids (thy), as well as 2.5μg protein of Arabidopsis microsomal membranes (mm) and chloroplast envelopes (env) were separated by SDS-PAGE and subjected to immunoblot analysis using antibodies directed against Ps-FAX1 and At-FAX1. Antisera against marker proteins LSU (str), LHCP (thy), NiCo (IE), OEP16.1 (OE), and TPR7 (mm, see [20]) were used as controls. For LSU and LHCP less protein (1μg, 0.2μg, respectively) was loaded. Numbers indicate molecular mass of proteins in kDa.

For all Tmemb_14 proteins, four hydrophobic α-helical domains are predicted (Fig. 1). However, nuclear magnetic resonance (NMR) structure determination of the human Tmemb_14 proteins TMEM14A and TMEM14C [14] showed that only three of these helices are membrane-spanning. TMEM14A contains an amphiphilic N-terminal helix, presumably located at the lipid micelle-water interface, while for TMEM14C an amphiphilic helix that orients perpendicular to the lipid bilayer, is placed between the second and third membrane domain. Amino acid sequences of the plastid FAX1 and the non-plastid At-FAX6 nicely align to both TMEM14A and 14C (Fig. 1C), but structural modeling revealed that the mature At-FAX1 and Ps-FAX1 are more similar to TMEM14C (Fig. 2A). Here, three membrane-spanning and one amphiphilic helix are likely, while the additional N-terminal amino acids of FAX1 proteins might form another α-helical domain not present in TMEM14C. In contrast, the structure of At-FAX6 resembles that of TMEM14A with an N-terminal amphiphilic helix followed by three transmembrane domains (Fig. 2B).

With its membrane-spanning domains, FAX1 inserts into the inner envelope membrane (IE) of plastids as could be shown by in vivo GFP-targeting and immunoblot analysis. At-FAX1-GFP signals in Arabidopsis protoplasts, which can be detected as rings around chloroplasts (Fig. 2C), point to an envelope localization. This could be confirmed and specified to IE by immunoblot analysis using sub-fractionated pea chloroplasts. In pea IE membranes as well as in Arabidopsis chloroplast envelopes, FAX1 signals appear as a band of about 25kDa (Fig. 2D). In agreement, FAX1 peptides in proteomic analyses of plastid membranes were exclusively detected in IE preparations (see [16] and references therein). To exclude ER localization, we further probed against ER-enriched Arabidopsis microsomal membranes (see [20]), where no FAX1 signals could be detected (Fig. 2D).

### Mutation of *FAX1* Affects Plant Biomass and Male Fertility

To study the in vivo function of FAX1, we analyzed loss-of-function and over-expressing mutant lines in Arabidopsis. We selected *fax1–1* and *fax1–2* with T-DNA insertions in the first intron and first exon of the *FAX1* gene, respectively (S1A Fig.). Reverse transcriptase-polymerase chain reaction (RT-PCR) analysis showed that both homozygous alleles represent knockouts for *FAX1* (S1B Fig.). To complement this loss-of-function, *At-FAX1* cDNA under control of the 35S promoter was introduced into heterozygous *fax1–2* plants. Subsequently, two lines homozygous for the *fax1–2* allele (Co#7 and Co#54) were selected for further analysis. To stable over-express *FAX1* in wild-type plants, the *35S*::*FAX1* construct was introduced into Col-0, and two independent lines named ox#2 and ox#4 were identified as FAX1 over-expressors. Quantitative real time RT-PCR revealed that *FAX1* transcripts in line Co#7 are at wild-type levels, whereas line Co#54 contains about 12 times more *FAX1* mRNA. Over-expression in ox#2 seedlings was mild (about 2-fold), but strong in line ox#4 (about 200 times more mRNA than in wild type; S1C Fig.). Immunoblot analysis confirmed the strength of FAX1 expression in these lines and the knockout in *fax1–2* on the protein level (S1D Fig.).

**Fig 3:**
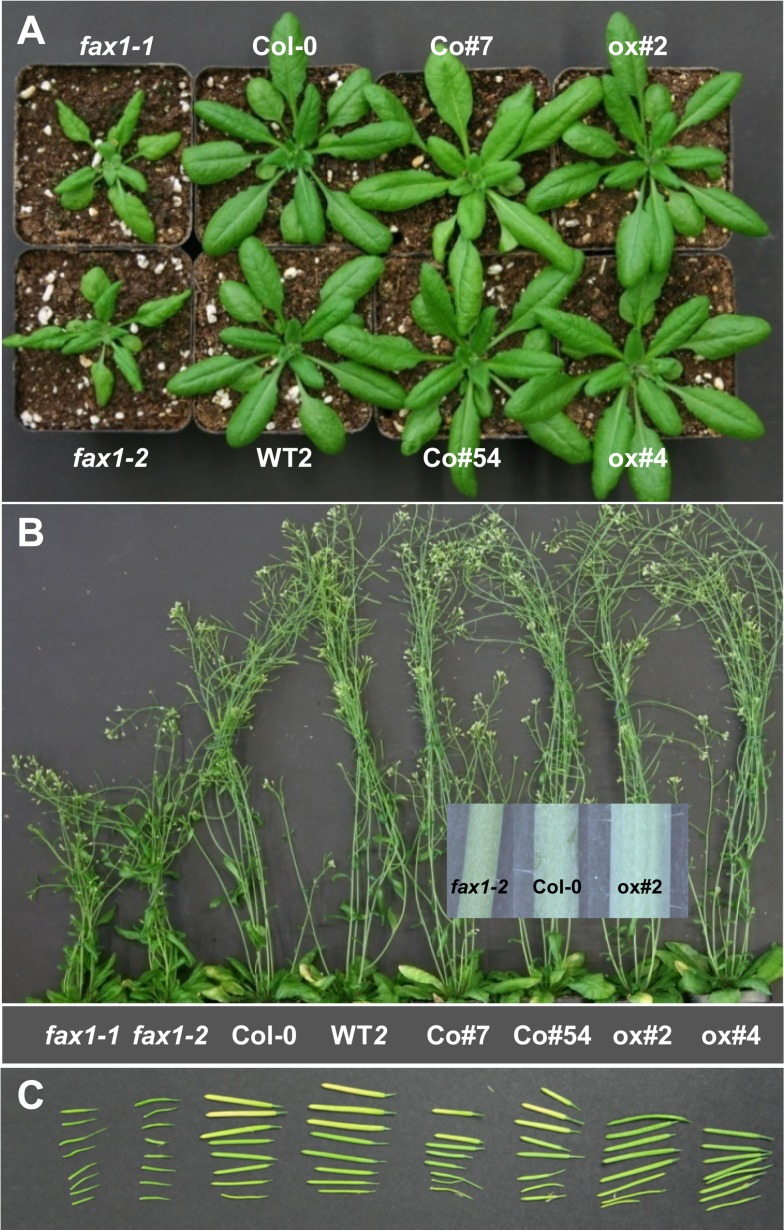
Mutation of *FAX1* in *Arabidopsis* affects plant growth. (A) 30-day-old plants of *FAX1* mutants and wild-type lines. *fax1–1*, *fax1–2*: homozygous knockout lines for *FAX1*; Col-0, WT2: wild-type *FAX1* alleles, WT2 represents *FAX1* wild-type progeny, segregated from heterozygous *fax1–2*; Co#7, Co#54: *fax1–2* knockout complementation lines; ox#2, ox#4: *FAX1* over-expressing lines in Col-0 background. (B) 7-week-old flowering plants of *FAX1* mutants and wild type as specified in (A). (Inset) Comparison of primary inflorescence stalks (bottom parts of 2^nd^ internode) from *fax1–2*, Col-0 and ox#2 plants. (C) Siliques produced by *FAX1* mutants and wild-type lines as depicted in (B).

Homozygous *fax1–1* and *fax1–2* knockout mutants both were characterized by reduced biomass at mature rosette stages (Fig. 3A, Table 1). Full flowering *fax1* knockouts were significantly smaller than wild type, had thinner inflorescence stalks, and flowers producing short siliques that contained almost no seeds (Fig. 3B, C). Detailed analysis of different tissues and organs revealed that the decrease in biomass of *fax1* lines was detectable throughout the entire plant body, including root, leaf, and stem tissues (Table 1). Because differences in stem dry weight were slightly more pronounced than in fresh-weight (FW) samples, most likely cell wall synthesis was affected. This could be confirmed by ultrastructural analysis of stem tissue (S2 Fig.). Here *fax1* knockouts showed small vascular bundles with reduced secondary cell walls (S2A, D, G Fig.). Since the same phenotype was detected in both independent T-DNA insertion lines *fax1–1* and *fax1–2*, and could be reverted by complementation in lines Co#7 and Co#54 (Fig. 3, Table 1), we conclude that the reduced biomass is caused by the loss of FAX1 function.

**Table 1:** Plant biomass of *FAX1* mutant lines and wild-type plants.

	*fax1–1*	*fax1–2*	Col-0	WT2	Co#7	Co#54	ox#2	ox#4
**Stem diameter** (mm; bottom part of 2^nd^ internode of primary infloresence stem; n = 4–13±SD)								
	**0.74 ± 0.08**	**0.91 ± 0.08**	1.24 ± 0.15	1.12 ± 0.08	1.12 ± 0.06	1.21 ± 0.10	**1.48 ± 0.14**	**1.43 ± 0.15**
p	0.00066	0.00014		0.07230	0.06407	0.36186	0.00824	0.02474
**Stem fresh weight** (mg/cm; 1 cm at bottom of 2^nd^ internode of primary inflorescence stem; n = 4–12±SD)								
	**5.05 ± 0.85**	**7.30 ± 1.48**	12.93 ± 2.98	10.87 ± 1.54	11.20 ± 1.33	13.57 ± 2.35	**18.52 ± 4.19**	**17.36 ± 4.04**
p	0.00014	0.00051		0.06406	0.11093	0.33961	0.01937	0.00909
**Stem dry weight** (mg/cm; same sample as for stem fresh weight; n = 4–12±SD)								
	**0.35 ± 0.12**	**0.73 ± 0.22**	1.46 ± 0.37	1.30 ± 0.24	1.38 ± 0.32	1.58 ± 0.58	1.57 ± 0.38	1.87 ± 0.56
p	0.00540	0.00429		0.04089	0.31021	0.48979	0.47439	0.07442
**Rosette fresh weight** (g; total mature rosettes; n = 8–13±SD)								
	**0.63 ± 0.14**	**0.57 ± 0.19**	1.12 ± 0.44	0.88 ± 0.13	0.89 ± 0.15	1.18 ± 0.31	**3.35 ± 1.23**	**2.67 ± 1.22**
p	0.00822	0.00080		0.29307	0.47241	0.09780	0.00001	0.00015
**Leaf fresh weight** (mg/0.09 cm^2^; centre of rosette leaf was punched as 0.09 cm^2^ disc; n = 6–11±SD)								
	**6.33 ± 0.50**	**6.36 ± 0.36**	8.87 ± 1.08	7.53 ± 0.38	7.68± 0.28	7.89 ± 0.87	**10.62 ± 0.58**	**10.06 ± 1.15**
p	0.00022	0.00016		0.04942	0.03075	0.06604	0.00122	0.00830
**Root weight** (g; total root tissue; n = 4–10±SD)								
	0.05 ± 0.01	**0.06 ± 0.01**	0.09 ± 0.02	0.07 ± 0.01	0.10 ± 0.02	0.10 ± 0.01	**0.14 ± 0.03**	**0.16 ± 0.03**
p	0.04269	0.00690		0.08435	0.24195	0.07855	0.00247	0.00060

7-week-old Arabidopsis plants from the respective *FAX1* mutants and wild-type lines as specified in the text were dissected into different organs, which were weighed and measured. Data that were significantly different when compared to Col-0 (Student’s *t-*test, *p* < 0.025) are in bold. Respective *p*-values (comparison to Col-0) are listed.

Remarkably, FAX1 over-expressing lines ox#2 and ox#4 were slightly bigger and produced more biomass as well as thicker inflorescence stalks than wild type (Fig. 3, Table 1), thus behaving opposite to *fax1* knockouts. In stems, this led to about one more hypodermal cell layer and to extended vascular strands, including an increased amount of xylem and phloem vessels, as well as a multi-layered procambuim (S2C, F, I Fig.). Because fresh weight of ox#2 and ox#4 stems was significantly higher than in wild type, but—in contrast to *fax1* knockouts—stem dry weight of FAX1ox lines was similar to wild type (Table 1), the increased biomass of FAX1 over-expressors is most likely mainly due to enhanced production of cells. However, since tracheid walls of ox#2 appeared to be slightly thicker than in Col-0 (S2H, I Fig.), we can’t fully exclude an additional effect on the size of secondary cell walls. Interestingly, the rate of FAX1 overproduction—i.e., 2-fold for ox#2, 200-fold for ox#4—did not quantitatively affect the strength of biomass phenotypes, indicating a rather non-linear effect of protein function.

To understand the peculiar loss-of-function phenotype of homozygous *fax1* knockouts during flower and silique development, segregation analysis of mutant alleles was performed. Self-fertilization of heterozygous *fax1–1* and *fax1–2* revealed that the ratio of homozygous progeny was 7% and 4%, respectively, pointing to defect male and/or female gametophytes (Table 2). However, when stigmata from homozygous *fax1–2* flowers were fertilized with wild-type *fax1–2* pollen, normal seeds with 100% heterozygous *fax1–2* mutant alleles were produced, indicating fertile *fax1* knockout female gametophytes and sporophyte organs. In contrast, pollination of wild-type stigmata with homozygous *fax1–2* anthers, produced short siliques, as observed during selfing of homozygous *fax1–2* mutants (see Fig. 3C), and led to an estimated seed yield <0.1% of wild type. Furthermore, during manual crossing it became evident that pollen grains of homozygous *fax1–2* flowers were improperly released from anthers. To minimize potential anther defects from the paternal sporophyte, we thus pollinated homozygous *fax1–2* stigmata with heterozygous *fax1–2* anthers, thereby producing 12% progeny homozygous for *fax1–2* (Table 2). In summary, these results point to impaired transmission of male gametophytes (pollen) and defects of the male sporophyte (anther) in *fax1* knockouts, finally leading to the observed male sterility.

**Table 2:** Segregation analysis of *fax1* knockout lines.

Crosses (♂ x ♀)	No.	ho %	he %	wt %
*fax1–1*(he) x *fax1–1*(he)	280	7 (25)	65	28
*fax1–2*(he) x *fax1–2*(he)	204	4 (25)	63	33
wt x *fax1–2*(ho)	92*	-	100	-
*fax1–2*(he) x *fax1–2*(ho)	171	12 (50)	88	-

Segregation of *fax1* knockout mutant alleles was analyzed by PCR-genotyping in the progeny produced by the respective crosses. ♂ x ♀: Mutant alleles of male and female gametophytes used for crossing. No.: Number of plants analyzed in the filial generation. “*fax1–1*(he) x *fax1-1*(he)” and “*fax1–2*(he) x *fax1–2*(he)” are self-pollinations of the heterozygous *fax1–1*, *fax1–2* mutants, respectively. “wt x *fax1–2*(ho)” represents the backcrossing of wild-type pollen with homozygous *fax1–2* female gametophytes. For “*fax1–2*(he) x *fax1–2*(ho)” heterozygous *fax1–2* pollen were crossed with homozygous *fax1–2* mutant female gametophytes. %: percentage of homozygous (ho, in brackets: expected values for normal Mendelian segregation) and of heterozygous (he) *fax1* mutant alleles, respectively; wt%: percentage of *FAX1* wild-type alleles.

* mix from 10 crossing events.

### FAX1 Function Is Essential for Pollen Cell Wall Assembly

**Fig 4:**
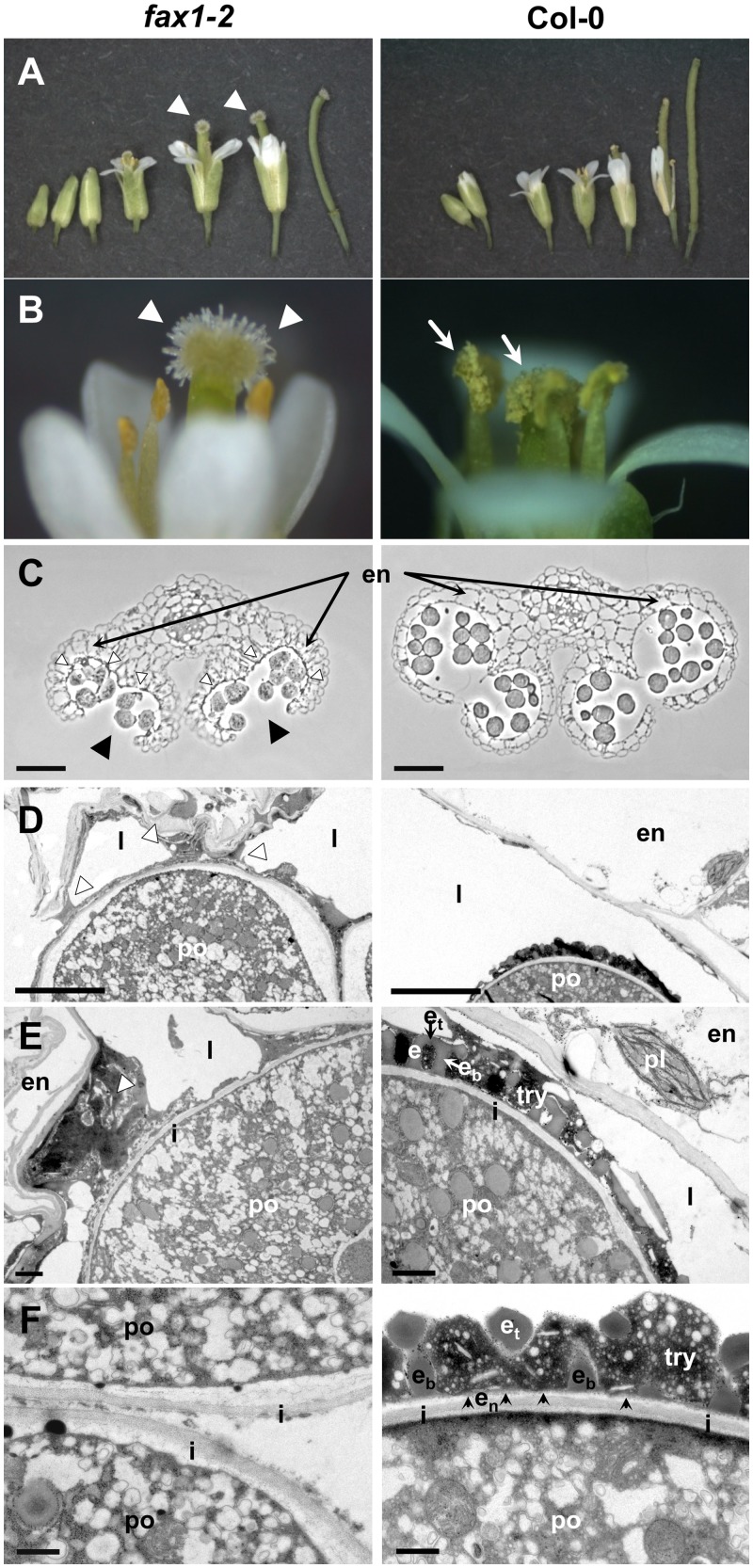
FAX1 function is essential for pollen cell wall assembly. Pictures of flowers, anthers, and mature pollen of 5-week-old *fax1–2* knockout and Col-0 wild-type plants (left and right panels, respectively). (A) Development of flower buds and young siliques. (B) Close-up of opened flowers. Arrowheads: non-pollinated stigma in *fax1–2*; arrows: anthers with released pollen in Col-0. (C) Cross sections of mature, dehisced anthers (light microscopy, bar = 50 μm). Black and white arrowheads in *fax1–2*: fully opened pollen sacks, and dark material covering endothecium/locule boundary, respectively. en: endothecium cells of anthers. (D), (E), (F) Transmission electron microscopic (TEM) pictures of anther cell/pollen grain intersections (D, bar = 5 μm; E, bar = 1μm) and pollen cell wall (F, bar = 500 nm) at mature tricellular pollen stages. White arrowheads in *fax1–2*: debris material sticking to pollen grains. en: endothecium cell; e: exine layer with e_b_: bacula, e_t_: tectum structures; e_n_: nexine layer (black arrowheads), i: intine layer; po: cytosol of pollen grain; pl: plastid; try: tryphine pollen coat.

To further analyze flower development, in particular that of male parts, we examined the morphology of flower tissue from *FAX1* mutant lines (Fig. 4; S3 Fig.). In *FAX1* wild-type and over-expressors (ox#2, ox#4), pollen release by anther dehiscence, transfer to the stigma, and fertilization as indicated by high yield of viable seeds was normal. However, flowers of *fax1* knockout mutants showed stigmata with non-pollinated papillae. In addition, *fax1* anthers released only very few pollen grains (Fig. 4A, B; S3A, B, G Fig.). In flowers of complemented *fax1–2* lines (Co#7, Co#54) in comparison, more free pollen grains than in *fax1* knockouts but less than in wild type were visible, indicating incomplete recovery of pollen release (S3D, G Fig.). In contrast to the rest of the plant organs, where regeneration of *fax1* knockout defects in Co#7, Co#54 lines was 100% (see Fig. 3; Table 1), complementation of *fax1–2* pollen phenotypes was incomplete. This effect was best visualized by the colorless pollen of *fax1* knockout and complementation lines (S3B, D, G Fig.), due to the absence (*fax1–2*) or incomplete (Co#54) assembly of a pollen coat (compare Fig. 4D–F; S3I–K Fig.) that normally includes yellow flavonoid and carotenoid deposits (for overview, see [2]). The incomplete complementation was restricted to pollen grains and most likely is due to the *35S* promoter system, which in Arabidopsis shows no or reduced activity in pollen grains and anther tissue, respectively [21].

Subsequently, the detailed structure of anther tissue and pollen grains of *fax1–2* knockout, Col-0 wild type, and the complementation line Co#54 was visualized by light- and transmission electron microscopy (TEM) at the mature, tricellular pollen stage (Fig. 4C–F; S3H–K Fig.). In general, anthers of *fax1–2* were smaller than in wild type and the surface of pollen grains appeared to be wrinkled (Fig. 4C). Cross sections revealed an impaired release of *fax1–2* pollen, although pollen sacks were wide open, indicating full dehiscence of anthers. Tapetum cells seemed to be degraded as expected for the developmental stage analyzed, however, the locule of *fax1–2* anthers was covered by an electron-dense material, which stuck to pollen grains and thus most likely was responsible for improper pollen delivery (Fig. 4C–E). Ultrastructural resolution demonstrated that the well-defined structures of the outer pollen cell wall—i.e., the exine layer and the pollen coat, which covers the exine surface and its cavities—were absent in *fax1–2* knockouts (Fig. 4E, F). The intine, representing the innermost layer of the pollen cell wall and composed of cellulose, pectin, and various proteins, secreted by the microspore (gametophytic origin, see [22]), however, looked intact. In contrast, mature wild-type pollen showed a complete exine, consisting of a flat nexine layer and the sculpted sexine parts tectum and bacula. Furthermore, the latter were filled and covered with the tryphine pollen coat (Fig. 4E, F). Pollen cell walls of Co#54 displayed an intermediate state of biogenesis with visible nexine layers, but incomplete arrangement of tectum and bacula structures as well as pollen coat material (S3J, K Fig.). As described above, these findings point to an incomplete complementation of *fax1–2* knockouts in pollen.

In conclusion, structural analysis of anthers and mature pollen grains showed that FAX1 is essential for biogenesis of the outer pollen cell wall, in particular for the assembly of exine and pollen coat. Both layers consist of complex biopolymers, such as sporopollenin (exine) and tryphine (pollen coat), that are mainly made of FA-derivatives and lipids originating from the tapetum tissue of anthers (sporophytic origin, see [2]). Thus, FAX1 might play a role in delivery of these compounds by mediating FA-export from tapetum cell plastids. Most likely, the electron-dense, sticky material in *fax1* knockout anthers that prevents release of pollen grains represents cellular debris of degenerated tapetum cells and/or not-incorporated sporopollenin or tryphine material.

### FAX1 Affects Cell Wall Size and Cuticular Wax Composition

**Fig 5:**
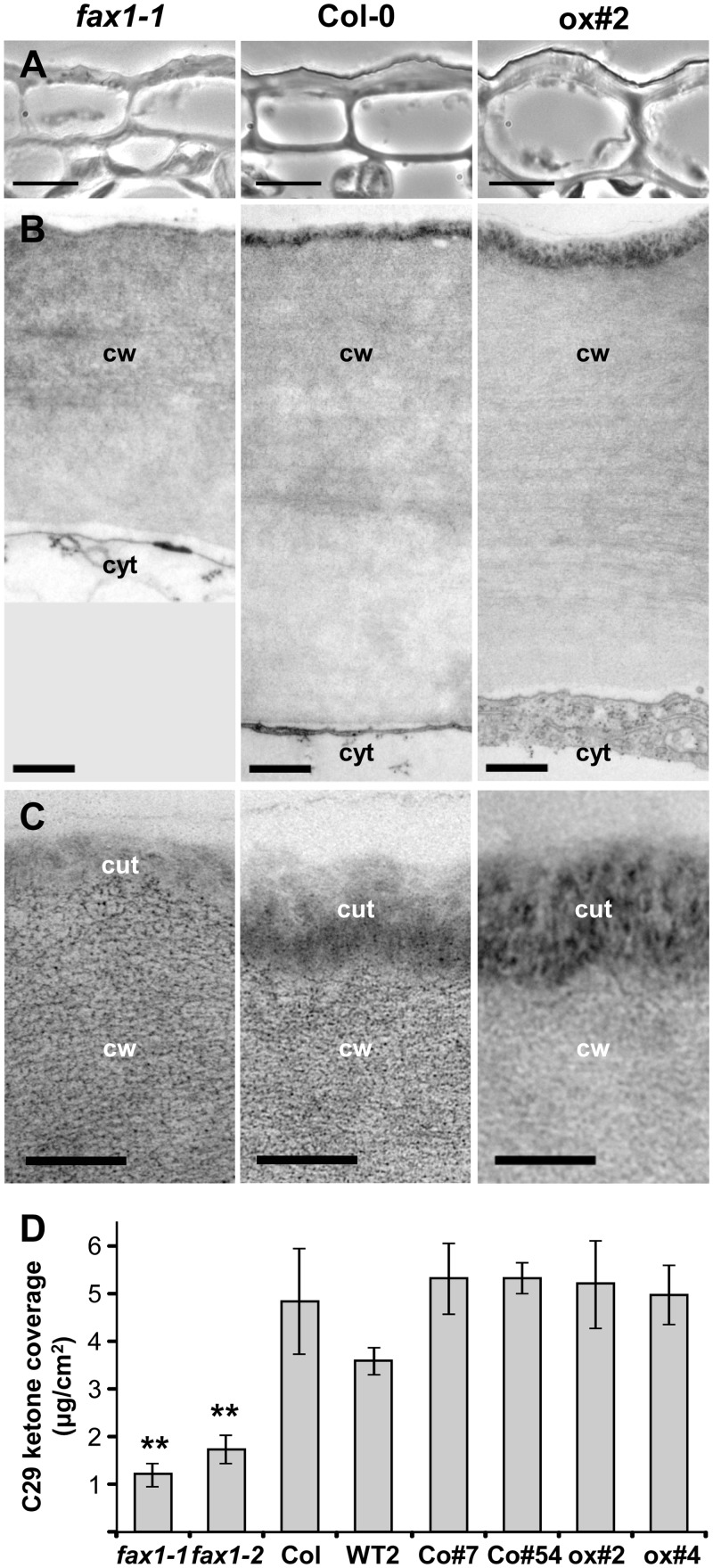
FAX1 affects cell wall size and cuticular wax composition. Stem tissue (1 cm at the bottom of the second internode) of the primary inflorescence stalk of 5-week-old *fax1–1* knockout, Col-0 wild-type, and *FAX1* over-expressor line ox#2 (left, middle, and right panels, respectively). (A) Light microscopic pictures of stem epidermal cells (bar = 10μm). (B) Transmission electron microscopic pictures of cell walls from stem epidermal cells (bar = 500 nm). (C) Close-ups of cell wall / cuticular layer boundary from cells in (B) (TEM, bar = 200 nm). cut: cuticular layer; cw: cell wall; cyt: cytosol. (D) C29 ketone wax coverage in μg per cm^2^ of stem surface from *FAX1* mutant and wild-type lines (*n* = 3–7 ± SD; *n* = 12 for Col-0). For each replicate, three to four stem sections between internode 2–4 of 7-week-old, mature flowering plants were pooled. Asterisks indicate highly significant different contents (**: *p* < 0.001, Student’s *t*-test) when compared with Col-0 (for numerical values, see S1A Data).

Because during analysis of *FAX1* mutants an altered surface of epidermal cells was apparent, we investigated structure as well as wax and cutin coverage of epidermis cells from primary inflorescence stalks of *FAX1* mutants (Fig. 5). Microscopic analysis revealed that the width of epidermal cell walls in *fax1–1* was strongly reduced when compared to wild type (Fig. 5A, B). As for cell walls in xylem vessels (S2 Fig.), a strong effect was only found for knockout and not for FAX1 over-expressing lines. However, an electron-dense cover at the extracellular side of the cell walls, most likely representing the cutin matrix of the cuticular layer, appeared to be more intense in ox#2, but reduced in *fax1–1* (Fig. 5B, C). To examine the constitution of the cuticular layer, we therefore determined wax and cutin coverage from stem epidermis cells. Surprisingly, the total loads of different wax and cutin species were not altered for all lines analyzed (*fax1–1*, *fax1–2*, Col-0, WT2, Co#7, Co#54, ox#2, ox#4, see S1A Data). Furthermore, no change in composition regarding aliphatic chain length or functional groups (e.g. ketones, acids or aldehydes) could be detected. The only significant difference we found was for C29-ketone wax components, which were reduced in both *fax1* knockout lines by more than 50% when compared to stems from all other plants (Fig. 5D).

Since cutin contents were unchanged, the different strengths of the outer layer of epidermal cell walls observed by TEM most likely are due to stronger (ox#2) and weaker (*fax1–1*) crosslinking of the cutin matrix with cell walls. The wax composition of cuticular layers, however, is dependent on plastid FA-synthesis as well as excretion of modified FAs via the plasma membrane of epidermis cells (for overview, see [1]). In parallel to the assembly of sporopollenin and tryphine material in pollen cell walls (see above), FAX1 might thus be necessary for plastid FA-export, previous to synthesis and release of ketone wax components.

### Plastid FAX1 Impacts Cellular FA and Lipid Homeostasis

Because *fax1* knockouts revealed a lack of FA- and lipid-derived compounds in pollen as well as stem epidermis cells (see above), we measured free FAs and polar lipid species such as phospho-, sulfo-, galacto-lipids, and triacylglycerols in leaves and flowers of mature *FAX1* mutant plants (S1 Table). To spotlight changes in *FAX1* mutants compared to wild type, we determined relative values and summarized representatives of significantly different levels, as well as abundant species from each molecule class in the next two figures. For comparison to the overall FA/lipid composition of each tissue, we listed contents in mol% of all significantly different species in S2–S4 Tables, and further estimated the impact of changes in mol% of each molecule class in the next table.

**Fig 6:**
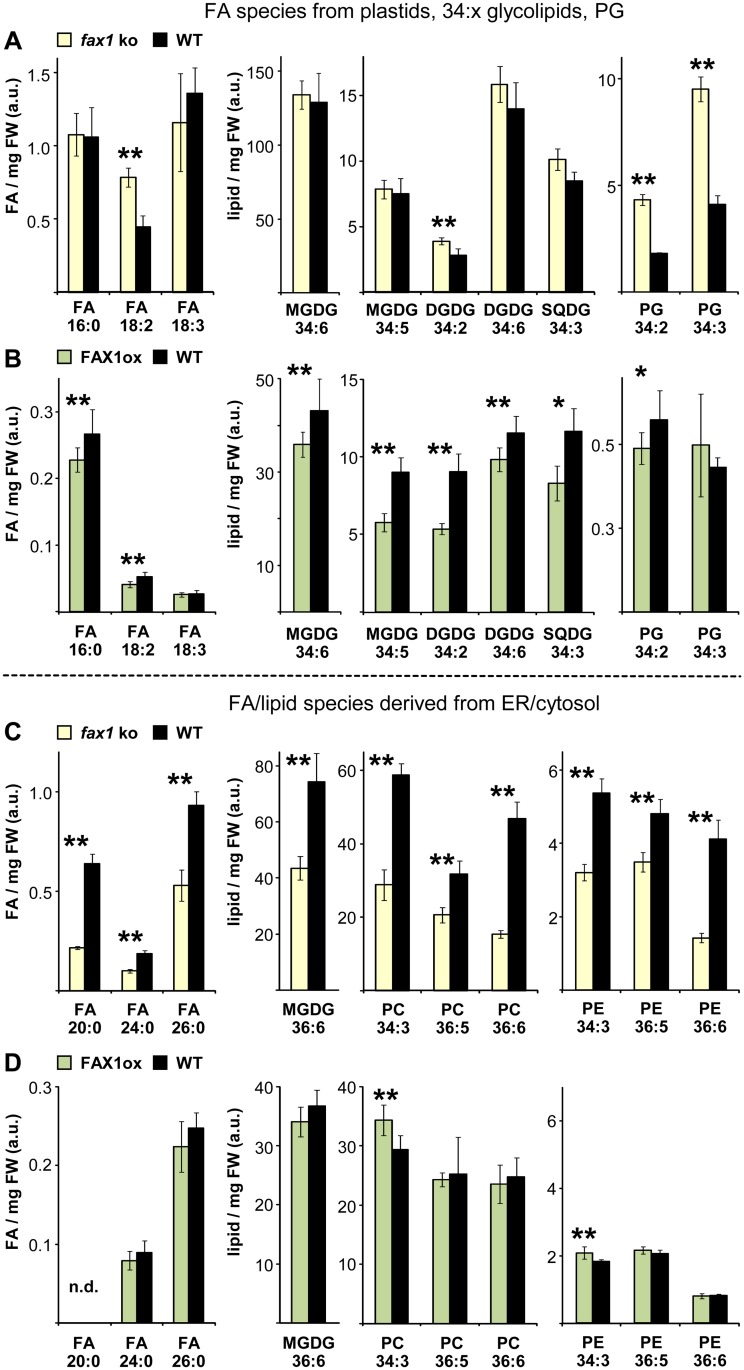
Plastid FAX1 impacts cellular FA/lipid homeostasis in leaves. Free fatty acid (FA) and polar lipid species were determined in leaf tissue of 7-week-old, mature flowering plants. Data (arbitrary units) are expressed relative to the internal standard (PC 34:0) and normalized to mg fresh weight (FW). For overview, we depict representatives of the most abundant species and those significantly different in *FAX1* mutants. A complete dataset with details on analysis is given in S1 Table; values in mol % are listed in S2 Table. Levels in FAX1 mutants significantly different to wild type are indicated by asterisks (Student’s *t*-test, *: *p* < 0.05, **: *p* < 0.01). For a better resolution of differential patterns, *y*-axes are scaled differently. C_16–18_ FAs are exclusively and glycolipids in (A) and (B) are mainly synthesized in plastids (for details, see Discussion). Please note that the diacylglycerol backbone for the “34”-glycolipids can originate from prokaryotic (from plastids) and eukaryotic (from the ER) phospholipid precursors, respectively. C_20–26_ FAs and phospholipids in (C) and (D), as well as precursors for “36”- glycolipids, are only produced outside plastids in the cytosol and/or ER. (A), (C) Free FA and lipid levels in caulinary leaves of *fax1–1*, *fax1–2* knockout and Col-0, WT2 wild-type lines (yellow and black bars, respectively) were determined by UPLC-Orbitrap MS [23]. Mean values (*n* = 6 ± SD), averaged over both *fax1* knockouts and both wild types, respectively, are shown. (B), (D) Free FA and lipid content (*n* = 6–12 ± SD) in caulinary leaves of the *FAX1* over-expressing line ox#4 and Col-0 wild type (green and black bars, respectively) was measured by UPLC-qTOF MS [24]. Please note that in comparison to the dataset in (A) and (C), usage of a different mass spectrometer results in different scaling of the relative values. DGDG: digalactosyl-diacylglycerol; FA: fatty acid; MGDG: monogalactosyl-diacylglycerol; n.d.: not determined; PC: phosphatidyl-choline; PE: phosphatidyl-ethanolamine; PG: phosphatidyl-glycerol; SQDG: sulphoquinovosyl-diacylglycerol.

In leaves of *fax1* knockout plants, levels of 30 FA and polar lipid species (irrespective of TAGs) were significantly different from wild type (S2 Table). For free FAs, we observed an increase of plastid-produced FA 18:2 (Fig. 6A, S2A Table) and a decrease for FAs 20:0, 24:0, 26:0 (Fig. 6C, S2C Table), which are elongated at the ER. Whereas aggregate levels of 34:x glycolipids (MGDG, DGDG, SQDG) were only slightly elevated (Table 3, S2A Table), the highly abundant MGDG 36:6 (11.7 mol% in wt) with an ER-made diacylglycerol backbone was considerably less (2.7 mol%) than in wild type (Fig. 6C, S2C Table). The eukaryotic-type DGDG 36:6, however, increased contributing about 0.7 mol% more to the overall lipid content (Table 3, S2C Table). Strong upward changes were observed for phosphatidyl-glycerol (PG) species (2.7- to 5-fold; Fig. 6A, S2A Table), leading to an entire gain of up to 3.2 mol% (Table 3) of these mainly plastid-derived phospholipids. In contrast, the ER-produced phospholipids phosphatidyl-choline (PC) and -ethanolamine (PE) mostly decreased in *fax1* knockout leaves (Fig. 6C, S2C Table). Here the effect, in particular of highly abundant PC 34:3, PC 36:6 (9.3, 7.4 mol% in wt), was especially strong and is estimated to primarily contribute to a total reduction of PCs by 8.8 mol% (Table 3). Whereas the overall decrease of PE was about 0.5 mol%, phosphatidyl-inositol (PI) contents showed a pronounced upward fold change, which, however, only very slightly contributed to the overall lipid composition, and thus leaf PI might rather be involved in signaling (Table 3, S2C Table).

**Table 3:** Impact of *FAX1* mutation on cellular FA/lipid homeostasis.

	***fax1* ko**: 55.5% (106/191)		**FAX1ox**: 52.8% (84/159)	
	mainly plastid	cytosol/ER	mainly plastid	cytosol/ER
	**FA (16:0, 18:0, 18:1)**	**FA (20:0, 24:0, 26:0)**	**FA (16:0, 18:0, 18:1)**	**FA (20:0, 24:0, 26:0)**
leaf	nd	-0.10	-0.02	nd
flower	+0.29	-0.10	+0.01	nd
	**MGDG (34:x)**	**MGDG (36:x)**	**MGDG (34:x)**	**MGDG (36:x)**
leaf	+0.09	-2.71	-2.75	-0.37
flower	+0.72	+0.88	-0.50	+0.004
	**DGDG (34:x)**	**DGDG (36:x)**	**DGDG (34:x)**	**DGDG (36:x)**
leaf	+0.37	+0.72	-1.85	nd
flower	+0.05	+0.98	-0.03	-0.14
	**SQDG(34:x)**	**SQDG (36:x)**	**SQDG(34:x)**	**SQDG (36:x)**
leaf	+0.01	+0.04	-0.94	-0.04
flower	nd	+0.01	-0.20	-0.42
	**PG**		**PG**	
leaf	+3.17		-0.02	
flower	nd		nd	
		**PC**		**PC**
leaf		-8.80		+2.96
flower		-1.09		-5.56
		**PE**		**PE**
leaf		-0.46		+0.08
flower		+0.42		-0.47
		**PI**		**PI**
leaf		+0.08		nd
flower		-0.26		nd
		**TAG**		**TAG**
leaf		-4.30		+3.20
flower		-7.22		+6.63

Depicted are differences in contents (mol%) of the respective FA/lipid molecule class in *FAX1* mutants versus wild type. Only values significantly different were used for calculation; for single data on each FA/lipid species, see S2–S4 Tables. In *fax1* knockout lines, in total 55.5% (106 of 191) and in FAX1 over-expressors 52.8% (84/159) of all species measured significantly changed (compare S1–S4 Tables). nd: no significant differences determined.

In leaves of FAX1 over-expressing lines (Fig. 6B, D; Table 3), we found an opposite distribution of free FAs and lipids as in *fax1* knockouts. Here, without counting TAGs, 28 molecule species were significantly different from wild type (S2B, D Table). Contents of all differentially regulated and mainly plastid-derived FAs, 34:x glycolipids (MGDG, DGDG, SQDG) as well as PG 34:2 dropped (Fig. 6B, S2B Table). A considerable impact on the total lipid content came from reduction of highly abundant molecules such as MGDG 34:5, 34:6; DGDG 34:2, 34:3; or SQDG 34:3, all with levels higher than 2 mol% in wild type. In consequence, the estimated overall reduction was about 2.8, 1.9, and 0.9 mol% for 34:x MGDG, DGDG, and SQDG, respectively (Table 3). For lipids produced by the eukaryotic pathway at the ER, we found a mild decrease of MGDG 36:5 (0.4 mol%) and only very minor changes (0.04–0.08 mol%) for SQDG 36:4, 36:5, and PE 34:3 (Table 3, S2D Table). The effect on PC contents, however, again was quite strong (total increase of about 3.0 mol%, see Table 3), including elevated levels of the abundant PC 34:1, 34:3, 36:2, and 36:3 (Fig. 6D, S2D Table).

When compared to leaves, flower tissue of *fax1* ko and FAX1ox lines showed a similar differential pattern of free FAs and lipids, which are presumably mainly produced via the prokaryotic pathway (Table 3, S3A, B Table). Whereas in *fax1*, FAs that after synthesis have to be exported from chloroplasts (i.e. 16:0, 18:0, 18:1) increased when compared to wild type (largest change for 16:0 = 0.24 mol%), the plastid internal FA 18:3 and the plastid external FA 24:0 decreased by about 0.1 mol% each (S3A, C Table). In FAX1 ox flowers only a minor increase of FA 18:0 was detected (S3B Table). As found in leaves, overall levels of 34:x MGDG, DGDG, and SQDG increased in knockouts but decreased in over-expressors (Table 3). The most prominent changes were for MGDG 34:6 (increase of 0.6 mol% in *fax1*, S3A Table) and for MGDG 34:5 (decrease of 0.3 mol% in FAX1ox, S3B Table).

For several lipid species, which are assembled at the ER, however, patterns in flowers were different and more diverse than in leaves. These included a rise in 36:x MGDG levels (dominated by +0.8 mol % of MGDG 36:6) in *fax1* knockouts (S3C Table); an increase and a decrease of about 0.45 mol% PE in *fax1* and FAX1ox, respectively (Table 3); as well as a strong reduction of PC in FAX1ox flowers (up to 5.6. mol%, Table 3). In *fax1* knockout flowers in contrast, PC (mostly PC 36:6 by-1.0 mol%; S3C Table) and also PI species (-0.26 mol%) significantly dropped (Table 3).

**Fig 7:**
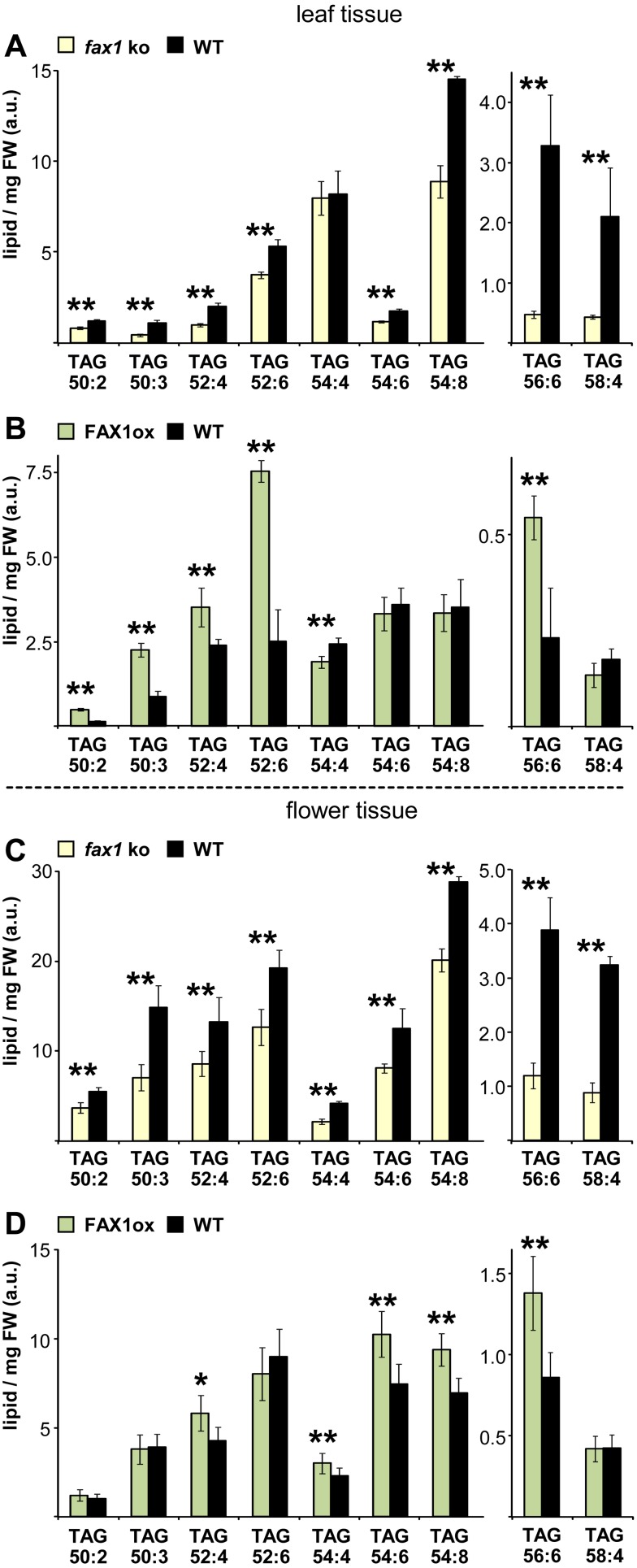
Plastid FAX1 impacts TAG storage lipid homeostasis. Triacylglycerol (TAG) oils were determined in tissues of 7-week-old, mature flowering plants (compare Fig. 6). Data (arbitrary units) are expressed relative to the internal standard (PC 34:0) and normalized to mg fresh weight (FW). For overview, we selected representatives of the most abundant species and those significantly different in *FAX1* mutants. A complete dataset with details on analysis is given in S1 Table; values in mol % are listed in S4 Table. Levels in FAX1 mutants significantly different to wild type are indicated (Student’s *t*-test, *: *p* < 0.05, **: *p* < 0.01). We show high and low abundant TAGs (left and right graphs, respectively); thus for better resolution of differential patterns, *y*-axes are scaled differently. (A) TAG levels in caulinary leaves of *fax1–1*, *fax1–2* knockout and Col-0, WT2 wild-type lines (yellow and black bars, respectively). Mean values (*n* = 4–6 ± SD), averaged over both *fax1* knockouts and both wild types, respectively, are shown. (B) TAG content in caulinary leaves of the *FAX1* over-expressing line ox#4 (green bars; *n* = 6–12 ± SD) and Col-0 wild type (black bars, *n* = 5–10 ± SD). (C) TAG levels in flowers of *fax1–1*, *fax1–2* knockout and Col-0, WT2 wild-type lines (yellow and black bars, respectively). Mean values (*n* = 6 ± SD), averaged over both *fax1* knockouts and both wild types, respectively, are shown. (D) TAG content (*n* = 7–12 ± SD, for TAG 58:6: *n* = 5) in flowers of the *FAX1* over-expressing line ox#4 and Col-0 wild type (green and black bars, respectively). Please note that in comparison to the dataset for *fax1* knockouts in (A) and (C), usage of a different mass spectrometer for FAX1ox data in (B) and (D) results in different scaling of the relative values (compare Fig. 6).

The most robust differential distribution in both mutant lines and tissues, however, was found for triacylglycerol oils (Fig. 7; Table 3). Here we determined significant changes for more than half of the molecules measured (S4 Table). More important, however, was a massive decrease of high and low abundant TAGs in *fax1* knockout leaves (Fig. 7A, S4A Table) and flowers (Fig. 7C, S4C Table) as well as a strong increase in FAX1ox leaves (Fig. 7B, S4B Table) and flowers (Fig. 7D, S4D Table). Fold changes were highest for low abundant TAGs (e.g., 8.3-fold decrease for TAG 56:7 in *fax1* leaves, S4A Table). As expected, the biggest impact on overall TAG content was by significant changes in high abundant species, resulting in a drop of-4.3 and-7.2 mol% in leaves and flowers of *fax1* knockouts and an accumulation of +3.2 and +6.6 mol% for leaf and flower tissue from FAX1ox lines, respectively (Table 3).

In summary, determination of free FAs and lipids in *FAX1* mutants clearly shows that the function of FAX1 in the IE membrane of chloroplasts impacts cellular FA and lipid homeostasis. Overall we found significant differences compared to wild type for more than 50% of all species determined (Table 3). Together with the observed lack of FA- or lipid-derived compounds in *fax1* knockout pollen cell walls and cuticular waxes (see above), these findings point to a function of FAX1 in FA-export from plastids (for details, see Discussion).

### FAX1 Is Able to Mediate FA-Transport into Yeast Cells

**Fig 8:**
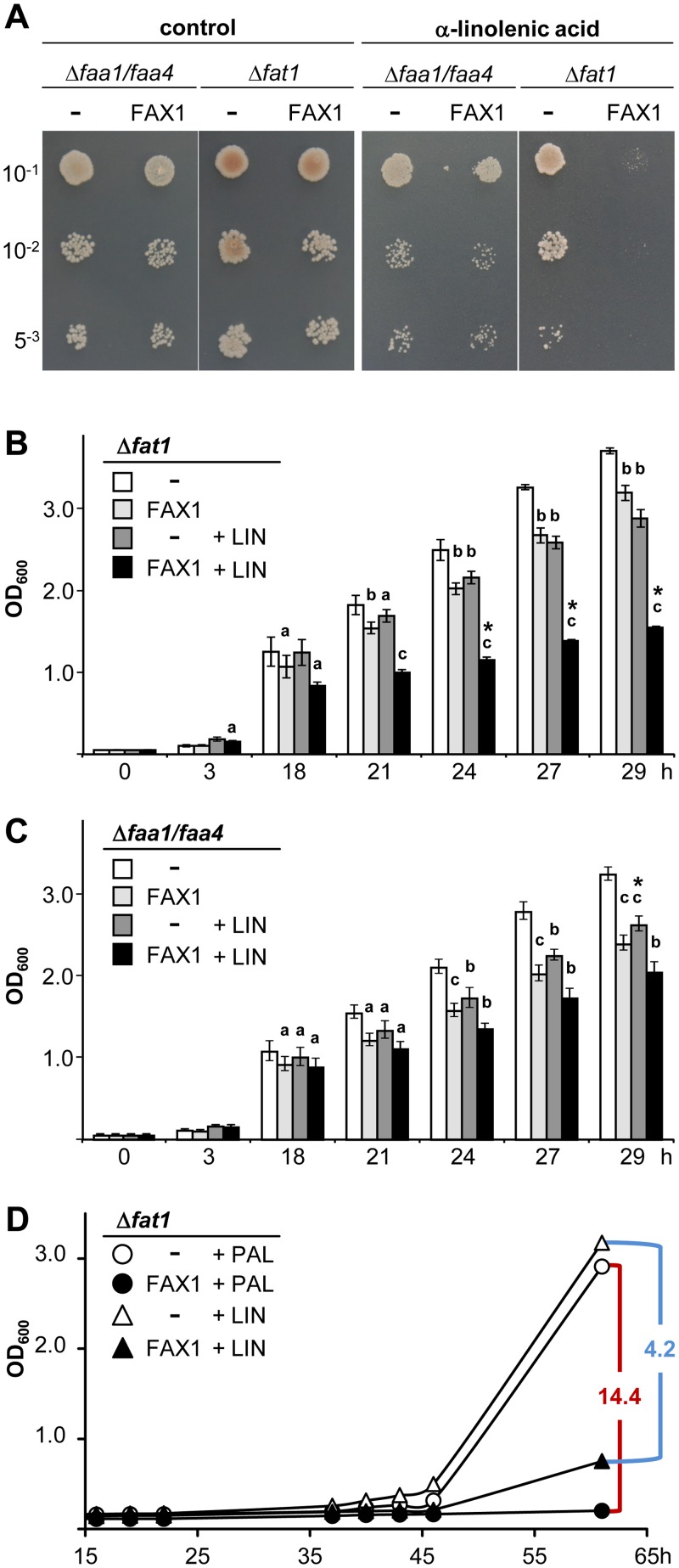
FAX1 mediates FA-transport in yeast. The empty plasmid pDR195 (-) and the mature At-FAX1 cDNA in pDR195 (FAX1) were introduced into *faa1/faa4* and *fat1* yeast mutants, respectively. (A) Serial dilutions (OD_600_ of 10^–1^, 10^–2^, and 5^–3^) of rapidly growing yeast cells on SD-ura plates (0.1% glucose, 1% tergitol). Control plates (left) in comparison to plates with 3.6 mM α-linolenic acid (C_18:3_; right). (B), (C) Growth of *fat1* (B) and *faa1/faa4* (C) cells in liquid SD-ura [see (A)]. The OD_600_ was monitored within 29 h incubation at 30°C. White and light gray bars: growth of pDR195 (-) and matFAX1/pDR195 (FAX1) at control conditions. Gray and black bars: growth of (-) and FAX1 in presence of 3.6 mM α-linolenic acid (LIN). Error bars depict SD (*n* = 4), for numerical values, see S1B Data. a (*p* < 0.05), b (*p* < 0.005), c (*p* < 0.0005), * (*p* < 0.00005) indicate significantly different values (Student’s *t*-test) compared to (-) cells without and with LIN, respectively. (D) Representative growth curves of *fat1* cells in liquid SD-ura (2% glucose, 0.5% Brij 58, 0.7% KH_2_PO_4_) in the presence of 10μM cerulenin (CER, inhibitor of FA-biosynthesis) and 100 μM palmitic acid (PAL, C_16:0_) or 100 μM α-linolenic acid (LIN, C_18:3_). Assays were performed according to [26,27]. For comparison with stearic (C_18:0_) and oleic acid (C_18:1_), see S4 Fig. White and black circles and triangles: growth of pDR195 (-) and matFAX1/pDR195 (FAX1) cells supplemented with PAL and LIN, respectively. Red/blue lines and numbers indicate maximal difference of cell density ratios for (-) versus FAX1 (compare S1B Data).

In baker’s yeast (*Saccharomyces cerevisiae*) import of exogenous long-chain FAs by the so-called vectorial acylation process requires a multiprotein complex, which consists of Fat1p (the membrane-spanning transport protein) and Faa1p or Faa4p, acyl-CoA synthetases for intracellular FA activation [25]. In order to test a potential FA-transport function of FAX1, we thus analyzed growth complementation of the yeast *fat1* and *faa1/faa4* mutants, which represent knockouts for Fat1p and Faa1p/Faa4p, respectively [26]. Therefore, we transformed the coding sequence of the mature At-FAX1 protein into *fat1* and *faa1/faa4* cells. Since previous studies revealed that the uptake of the polyunsaturated FA α-linolenic acid (C_18:3_) into yeast cells was toxic for wild-type but not for *fat1* cells [5], we challenged growth of FAX1-containing yeast mutants by addition of high α-linolenic acid concentrations (3.6 mM) to the media (Fig. 8A–C). In drop tests on agar plates, all cells showed normal growth under control conditions (Fig. 8A, left). In addition, yeast mutant cells, transformed with the empty vector only, were resistant to excess α-linolenic acid (Fig. 8A, right). However, *fat1* cells expressing the mature At-FAX1 protein died in the presence of α-linolenic acid overload (Fig. 8A, right), indicating that FAX1 is able to restore FA-uptake in *fat1* mutants. In contrast, α-linolenic acid induced cell death was not observed in *faa1/faa4* cells, neither with nor without FAX1, pointing to a FAX1 function in FA-transport and not in FA-activation.

Furthermore, we monitored a very similar behavior for growth kinetics of the respective yeast cells in liquid media (Fig. 8B, C). Here, a FAX1-mediated toxicity of α-linolenic acid was significant after 18 h when compared to empty vector cells. While this effect was highly significant and strong in *fat1* mutants as indicated by a reduction of cell density to about 54% after 29 h (Fig. 8B), only a mild growth inhibition was detected in Δ*faa1/faa4* (density of FAX1 cells was about 78% of control cells after 29 h; Fig. 8C). In addition, when compared to vector-only cells grown without α-linolenic acid, we observed a slight growth reduction by addition of α-linolenic acid itself as well as for FAX1-transformed cells in absence of α-linolenic acid, independent of yeast mutant strains (Fig. 8B, C). Whereas the former observation can be explained by unspecific, background uptake of α-linolenic acid provided at excess external concentrations, the latter effect might be due to a general, but minor, toxic effect of FAX1 expression in yeast.

To assess specificity of FAX1 for FAs, which have to be exported from chloroplasts in vivo, i.e., palmitic (C_16:0_), stearic (C_18:0_), and oleic acid (C_18:1_), we performed additional yeast growth complementation assays in the presence of the FA-biosynthesis inhibitor cerulenin and supply of moderate external FA concentrations (i.e., 100 μM; Fig. 8D, S4 Fig.). Results with rapidly (S4A–C Fig.) and non-exponentially growing cells (S4D Fig.) allowed definition of a potential substrate specificity of FAX1, preferring C_16:0_ over C_18:1_ and C_18:0_ FAs (for details see S4 Fig. and Discussion). When we tested α-linolenic acid (C_18:3_), which in planta is not exported from plastids (see [1]), as a control in this assay, FAX1 specificity was in the range as for stearic/oleic acid, but significantly lower than for palmitic acid (Fig. 8D, S4B Fig.).

## Discussion

In summary, our results show that the protein FAX1 in the IE of plastids is able to mediate FA-export as supported by the following findings: (i) FA-transport function of FAX1 in yeast; (ii) differential distribution of ER- and plastid-derived FAs/lipids in *FAX1* mutant plants; (iii) male sterility of *fax1* knockout lines, caused by impaired delivery of FA-derived compounds; (iv) decreased ketone wax compounds in cuticular layers of *fax1* knockout stems; (v) a focus on differential expression of genes for acyl lipid as well as carbohydrate and cellular/cell wall metabolism in *FAX1* mutant lines (see S1 Text).

### On the Function of FAX1

Complementation of the yeast *fat1*, but not of the *faa1/faa4* mutant, indicates that FAX1 is acting only in membrane transfer of FAs and not in FA-activation. This is in contrast to, for example, yeast and human FA-transporters such as Fat1p and FATPs, which in addition have acyl-activating functional domains [28,29]. FAX proteins group into the Tmemb_14 family and thus most likely contain three hydrophobic, membrane-spanning α-helical domains and one amphiphilic helix at the lipid bilayer/water interface. Thus, it is tempting to speculate that the latter might be responsible for binding and transfer of FA-chains across the IE membrane. Once loaded with a FA produced in the plastid stroma, this α-helix might become lipophilic enough to fold into the lipid bilayer and flip FAs over the IE. Furthermore, FAX1 and also FAX2 (see Fig. 1B) contain an extended N-terminal region (gray helix in Fig. 2A). Structural modeling indicates that these stretches fold into additional, most likely non-membrane associated α-helices: one for FAX1, two for FAX2, respectively. Interestingly, the two anti-parallel helices of the FAX2 N-terminus fit to sequence and structure of a ‘four-helical up-and-down bundle’ of the human apolipoprotein apoE3, which is involved in lipid transport and binding during formation of lipoprotein particles. Amphiphilic α-helices in the C-terminus of apoE3 are described to bind to lipids and thereby induce a conformational change in the N-terminal helix bundle that allows detergent-like solubilization of lipids and formation of lipoprotein particles (for overview, see [30,31]). Therefore, the N-terminal helices of plastid FAX proteins might be involved in similar functions during FA-transport. The different apparent molecular weights observed for FAX1 (S1D Fig.), most likely resulting from discrete conformations and/or packing of membrane domains, support these hypotheses for a transport mode. Once at the intermembrane space, FA-handover from FAX1 to substrate binding proteins, and transport across the OE membrane via a ß-barrel protein might be possible. For plastid re-import of eukaryotic lipids for example, the latter two proteins are represented by TGD2 (substrate binding) and TGD4 (OE ß-barrel, [3,11]). Furthermore, in *E*. *coli*, a similar system has been described for export of lipopolysaccharides, including an ABC transporter that flips the lipid moiety across the inner membrane, transfer proteins in the periplasm, and a ß-barrel protein in the outer membrane [32]. For plastids, subsequently an acyl-CoA synthetase (ACS) at the cytosolic face of the OE might finally drive FA-transfer in a passive, carrier-like process. Co-expression of At-FAX1 with LACS4 (ATTED-II coexpression networks), and regulation of LACS1, 3, and 5 expression in *FAX1* mutants (S5 Table, S6 Table) underline a possible cooperation with ACS.

The close structural similarity of FAX proteins to the human TMEM14A and TMEM14C, which both localize to mitochondrial membranes, in the future might enable explanation of TMEM14 protein function in vertebrates. Whereas TMEM14C was identified to coexpress with the core machinery of heme biosynthesis and its knockdown causes anemia in zebrafish [12], TMEM14A was described to stabilize mitochondrial membrane potential and thereby inhibit apoptosis in a yeast system [13]. However, their exact biological function is still unknown. Since animal mitochondria are the site for FA-degradation via ß-oxidation, a role for TMEM14 proteins in FA/lipid homeostasis, energy metabolism or disease (e.g., apoptosis) in vertebrates might be possible.

### FAX1 Function Impacts Cellular Lipid Homeostasis

Levels and subcellular distribution of free FAs and polar lipids in Arabidopsis *FAX1* mutants mainly correlated with a FA-export function, by which FAX1 influences cellular FA and acyl lipid homeostasis (for overview, see [1]). Most likely because of their toxicity and high metabolic fluxes for primary metabolites, changes in free FAs were not very pronounced. However, very-long–chain FAs (C_20_), which are elongated outside plastids and thus require previous export of C_16–18_ FAs, were significantly reduced in *fax1* knockouts (Table 3). According to acyl-ACP synthesis rates and specificity of thioesterases in *Arabidopsis* chloroplasts, oleic acid (C_18:1_) is the major free FA exported from chloroplasts, followed by palmitic acid (C_16:0_) and only very little amounts of stearic acid (C_18:0_; compare [1,33,34]). FAX1 in yeast assays performed best for FA 16:0 (determined specificity range: 16:0 > 18:1 ~ 18:0 ~ 18:3) and thus, most likely, mainly is involved in the plastid export of free palmitic acid but also can transport oleic acid, which at the stromal side of the plastid IE is provided at highest substrate concentrations. The fact that in yeast, FAX1 was also able to transport α-linolenic acid (C_18:3_), which in planta is retained inside plastids, indicates that the protein does not discriminate between different degrees of unsaturation, but in general prefers C_16_ over C_18_ FAs. In chloroplast IE membranes, FAX1 most likely functions in a passive, carrier-like mode, driven by concentration gradients of free FA substrates (see above). Interestingly, accumulation of export-directed C_16–18_ FAs in flower tissue of *fax1* knockouts (+0.24 > +0.04 > +0.01 mol% for 16:0 > 18:0 > 18:1), reflect the substrate specificity range of FAX1 in yeast (compare S3A Table and S4 Fig.). Furthermore, non-exported FA 18:3 significantly decreased (0.14 mol%; S3A Table) in flowers of *fax1* knockouts, thereby maybe pointing to stronger fluxes of FAs into plastid-intrinsic pathways (e.g., synthesis of oxilipin hormones), due to a block in FA export via FAX1.

Besides changes in free FAs, 65% of the differential lipid patterns depicted in Table 3 underline the hypothesis of plastid FA-export via FAX1, best documented by the strong reciprocal changes in TAG oils. Here for almost 90% of all significantly distributed TAGs (compare S4 Table), the pattern in both *FAX1* mutants and tissues perfectly matched to a FA-export function of FAX1. The distribution of 34:x glycolipids (MGDG, DGDG, SQDG), which increased in *fax1* knockouts but decreased in FAX1 over-expressors, also corresponded to our theory. In this case, we can, however, not exclude a contribution of ER-made species, since the diacylglycerol backbone for the “34”-glycolipids can originate both, from prokaryotic (from plastids) and eukaryotic (from the ER) phospholipid precursors, respectively. Yet, *Arabidopsis* is a so-called 16:3 plant, which for galactolipids prefers the prokaryotic pathway with high levels of 16:3 acyl chains. In contrast, ER-derived “34” DAG-backbones contain 16:0 saturated acyl moieties (compare [1,34]). Thus, we can assume that MGDG 34:x and DGDG 34:x with more than four desaturated C-bonds are completely synthesized in plastids. For the strong MGDG 34:x reductions in FAX1ox leaves and flowers (2.8 and 0.5 mol%, Table 3) and the increase in *fax1* flowers (+0.7 mol%) indeed 34:4, 34:5 and 34:6 are the major contributing species (see S2 Table, S3 Table) and therefore support our hypothesis. Most likely at least the abundant forms of phosphatidyl-glycerol (PG 34:3, 34:4) are exclusively made inside plastids as well (see [1]), and thus the pronounced overall increase of PG in *fax1* leaf tissue (+3.2 mol%) also mainly is due to a block of FA-export via FAX1.

Our assumption that FAX1 mediates plastid FA-export is further confirmed by a large decrease of PC-levels in *fax1* knockout tissues (up to 8.8 mol%) and a strong increase of PC in FAX1ox leaves (+3.0 mol%). However, also, considerable contrasting evidence is found for three ER-made lipids in flower tissue (i.e., +0.9–1.0 mol% MGDG 36:x, DGDG 36:x in *fax1*; -5.6 mol% PC in FAX1ox). The latter findings that only apply to lipid species synthesized in the cytosol/ER of flowers might be explained by the inhomogeneity of mature flowers, consisting of leaf, stalk, pollen, ovary, and seed/silique tissues, and/or by a preferential flow of FA-building blocks for lipids into TAG oils during seed development. Furthermore, for the bilayer-forming DGDG 36:x, a plastid export is described to act as surrogate lipid for the lack of PC at, e.g., phosphate-limited growth conditions (see [1] and references therein). Thus, the observed increase of DGDG 36:x species in *fax1* knockout mutants might compensate for the strong decrease of PC in the same tissues (compare Table 3).

In summary, levels and subcellular distribution of free FAs and polar lipids in *Arabidopsis FAX1* mutants mainly support a plastid FA-export function of FAX1. In addition, we can, however, not exclude plastid FA-export via different mechanisms or a bypass by other plastid FAX proteins (see below). Due to this potential functional redundancy of plastid FAX proteins, mutation of FAX1 alone does not affect all lipid species present in plants. Effects in leaf tissue, in particular of *fax1* knockouts are somewhat more straightforward and stronger than in FAX1 over-expressing lines. The latter is not unexpected for mutation of a protein involved in transport, which is highly expressed in leaf tissues (see below and S5 Fig.). However, the impact of FAX1 mutation on TAG-oil levels might be of future biotechnological importance. Interestingly, already the enhanced FA-transport by FAX1 was able to significantly increase TAG contents in leaves and flower tissues. Furthermore, our finding is in line with higher TAG when FA-loading to the ER in seeds is improved by over-expression of the ABC transporter ABCA9 [10]. Thus, coupling of the bottlenecks FA-transport (e.g., FAX1, ABCA9) with those of FA-synthesis and acyl-editing processes and a seed-specific expression might boost plant oil production in future approaches.

### The Role of FAX1 in Plant Development

Transcripts of *At-FAX1* are present in all developmental stages and peak in leaf tissues (cotyledons, rosette, caulinary, senescent leaves, and flower sepals) as well as in early pollen development (S5A–C Fig.). Consequentially, the strongest phenotype of *fax1* knockout mutants can be observed during growth (e.g., reduced rosette leaf size and biomass) and in particular in pollen grains, leading to almost complete male sterility due to the absence of pollen cell walls and impaired pollen release by anthers. For FAX1, we propose a function in FA-export from plastids of tapetum cells in anthers, which in *fax1* knockouts leads to the strongly impaired assembly of exine layers and pollen coat, most likely because of the lack of FA-precursors for sporopollenin and/or tryphine synthesis (for a detailed description, see S2 Text).

Since FAX1 in *Arabidopsis* belongs to a family of seven proteins, the plastid-predicted FAX2, 3, and 4, whose expression is regulated throughout plant development as well (S5 Fig.), most likely can bypass the loss of FAX1 function in all tissues and organs, leading to the rather mild overall phenotype of *fax1* knockouts. Especially in seed tissue (S5D, E Fig. and S6 Fig.), FAX2, 3, and 4 most likely play a more prominent role than FAX1. Indeed transcripts for *FAX2* and *FAX3*—i.e., the highest plastid *FAX* genes in seed development and germination—showed to be significantly up-regulated (1.13- and 1.24-fold) in *fax1* knockout flowers. Please note that with a relative signal of 1585 in wild type, *FAX2* and *FAX3* are strongly expressed in flower tissue we used for microarray analysis (among highest 9% of all genes on the chip; compare microarray dataset E-MTAB-3090 at www.ebi.ac.uk/arrayexpress).

The function of FAX1 in FA-delivery for pollen cell wall as well as for cuticular wax assembly is further underlined by differential gene expression in *FAX1* mutants (see S1 Text, S5 Table, S6 Table), and by the occurrence of phenotypes similar to *fax1* when biosynthesis pathways for FA/lipid-derived precursor material are mutated in *Arabidopsis*. These include a plastid-intrinsic fatty-acyl-ACP reductase (AlcFAR2/MS2), involved in primary fatty alcohol synthesis for anther cuticle and pollen sporopollenin formation [35]; as well as cytochrome P450 enzymes (CYP703A2, CYP704B1; [36,37]) that hydroxylate FAs, and the ACS ACOS5 [38] that activates FAs for sporopollenin synthesis in the cytosol of anther cells. Furthermore, several long-chain ACS (LACS1, 2, 4) are necessary to activate long-chain and very-long–chain FAs for building of cutin and wax as well as pollen exine layers [39,40]. In addition, several ABC transporters in the plasma membrane are required for deposition of surface lipids, displaying *fax1*-like phenotypes upon mutation: ABCG26 for pollen exine formation from tapetum cells, as well as ABCG11, G12, and G13 in lipid export from epidermis cells for formation of cuticular wax layers (for overview, see [41]). As for FAX1, pathways for synthesis of precursors of pollen cell wall and cutin/wax components often overlap. In stems of *fax1* knockouts, we further identified strong regulation of two genes involved in wax biosynthesis: AlcFAR3/CER4 and CYP96A15/MAH1 (S6 Table). Because the latter enzyme is catalyzing synthesis of wax ketone components, its differential expression is in line with the observed lack of C29 ketones in *fax1* knockout stems.

Besides deranged acyl lipid homeostasis, obviously also carbohydrate and cellular/cell wall metabolism was affected in *FAX1* mutants as reflected by the impact on plant biomass production and differential regulation of gene expression (see S1 Text, S7–S10 Figs.). In general, we assume that these effects are rather secondary and most likely might result from still unknown signaling events, due to changed FA/lipid homeostasis. Since *fax1* knockout plants are short of energy-rich lipids, they most likely turn down anabolic carbohydrate metabolism required for polysaccharide synthesis, resulting in, e.g., reduced biomass and secondary cell walls. The opposite effect is observed in *FAX1* over-expressors, in which an excess of lipids most likely leads to more biomass and the production of additional cell layers in stems. These observations clearly demonstrate regulation of energy metabolism and a close correlation between the availability of FAs/lipids and the utilization of carbohydrates in growth processes. This link is further underlined by the finding that the flow of carbon into oil can be promoted by activating plastid FA synthesis and repressing starch synthesis [42]. In this context FAX1, to our knowledge, is not only the first membrane protein identified that mediates FA-export from plastids, but FAX1 and its relatives represent key transport proteins and thus—together with enzymes of FA/lipid-synthesis and modification—might provide powerful future tools to modulate plant lipid and bioenergy production [43].

## Materials and Methods

### Plant Material and Growth Conditions

Experiments were performed on *Arabidopsis thaliana* ecotype Columbia (Col-0, Lehle Seeds; Round Rock, United States). The T-DNA insertion lines SAIL_66_B09 (*fax1–1*) and GABI_599E01 (*fax1–2*) were purchased from NASC (Nottingham Arabidopsis Stock Center, Nottingham, United Kingdom) and GABI-Kat (MPI for Plant Breeding Research, Köln, Germany), respectively. To generate complementation lines of *fax1–2* and over-expressing *At-FAX1* under the control of the 35S promoter, the coding sequence of *At-FAX1* was subcloned into pH2GW7 [44]. At-FAX1/pH2GW7 was transformed into *Agrobacterium tumefaciens* GV3101, which was used to transfect heterozygous *fax1–2* and Col-0 plants as described [45]. Arabidopsis seeds were sown on soil, vernalized at 4°C in the dark for two days, and grown in a 16 h light (22°C; 100 μmol photons ⋅ m^–2^ ⋅ s^–1^) and 8 h dark (18°C) cycle.

### Isolation of FAX1 cDNA Molecules

At-FAX1 cDNA was purchased as SSP pUNI51 clone U12755 [46]. The corresponding mRNA (NCBI reference sequence NM_15588) is predicted to be 1,030 bp long, including 180 bp 5´- and 169 bp 3´-untranslated regions (UTRs; S1A Fig.). For amplification of FAX1 from pea, RT-PCR was performed using pea seedling cDNA as template and oligonucleotide primers designed according to a pea EST contig sequence [47]. The corresponding mRNA molecule was 1,115 bp long, with 143 bp 5´UTR, 699 bp coding region, and 273 bp 3´UTR (GenBank accession no. KF981436). For primer sequences, see S7 Table; for amino acid sequences, see Fig. 1A.

### In Vivo GFP-Targeting

To generate a fusion of GFP to the preprotein At-FAX1, the coding sequence was subcloned into the p2GWF7 plasmid [44]. p2GWF7 provides a fusion of GFP to the C-terminal end of the respective proteins, which are expressed under the control of the constitutive 35S promoter. Transformation and analysis of *Arabidopsis* mesophyll protoplasts was performed as described [45]. GFP fluorescence was detected at 672 to 750 nm and chlorophyll autofluorescence was monitored at 503 to 542 nm by confocal laser scanning microscopy (Leica TCS SP5/DM 6000B, argon laser, excitation wavelength of 488 nm).

### Protein Extraction and Immunoblot Analysis

Pea chloroplasts isolated from leaf tissue of 10-day-old pea plantlets were sub-fractioned into OE and IE membranes, stroma and thylakoids as described [48]. Chloroplast envelopes, total protein extracts, and microsomal membranes from *Arabidopsis* plants were prepared as specified in [45] and [20], respectively. FAX1 antisera were raised in rabbit (Pineda Antibody Service, Berlin, Germany) against N-terminal peptide sequences of At-FAX1 (17 aa) and Ps-FAX1 (18 aa), respectively (see Fig. 1A). Antisera for marker proteins were produced as described previously [45,49]. Appropriate amounts of organellar or total cellular proteins were separated by SDS-PAGE, transferred to PVDF membranes and subjected to immunoblot analysis using primary antisera in 1:250 to 1:5000 dilutions in TTBS buffer (100 mM Tris-HCl pH 7.5, 150 mM NaCl; 0.2% Tween-20; 0.1% BSA). Non-specific signals were blocked by 3% skim milk powder and 0.1% BSA. Secondary anti-rabbit IgG alkaline phosphatase antibodies (Sigma-Aldrich) were diluted 1:30,000. Blots were stained using the alkaline phosphatase reaction with 0.3 mg/ml nitroblue tetrazolium (NBT) and 0.16 mg/ml bromochloroindolyl phosphate (BCIP) in 100 mM Tris pH 9.5, 100 mM NaCl, 5 mM MgCl_2_.

### Genotyping of *FAX1* Mutant Lines in *Arabidopsis*

Genomic DNA of the T-DNA insertion lines *fax1–1* and *fax1–2* was screened by PCR genotyping. To identify plants with T-DNA insertion in both *At-FAX1* alleles (homozygous), combinations of gene-specific primers that flank the predicted insertion sites with each other and with T-DNA-specific left border (LB) primers (S7 Table) were used. Positions and orientations of T-DNA inserts and oligonucleotide primers in *fax1–1* and *fax1–2* are shown in S1A Fig. To verify insertion sites, PCR-genotyping products were sequenced. T1 generations of generated *FAX1* over-expression and complementaion lines were selected by hygromycin (30 μg/ml). Stable insertion of *35S*::*FAX1* was controlled by PCR-genotyping in all subsequent generations. Therefore, a vector-specific primer in combination with a *FAX1* cDNA specific primer was used (S7 Table). In the T2 generation, complementation lines were selected for homozygous alleles of the original T-DNA insertion in *fax1–2* (see above), resulting in lines Co#7 and Co#54. For FAX1 over-expression in Col-0 background, we selected the lines ox#2 and ox#4 in the T2 generation.

### Microscopic Analysis

For microscopic analysis we used 5-week-old plants and dissected anthers from mature flowers or cut 1–2 mm^2^ stem segments 1 cm above the bottom of the second internode of the primary inflorescence stalk. We analyzed four individual *fax1–2* knockouts, two of each Co#7, Co#54 complementation lines, and five Col-0 wild-type plants for anthers/pollen grains, and pictured stem tissue of independent *fax1–1*, *fax1–2* knockouts, three ox#2, four ox#4 over-expressors, as well as seven individual Col-0 wild-type plants, respectively. Tissue was fixed immediately after harvest with 2.5% (w/v) glutaraldehyde (4°C, at least 24 h) in 75 mM cacodylate buffer (2 mM MgCl_2_, pH 7.0), rinsed several times with fixative buffer, and subsequently post-fixed with 1% (w/v) osmium tetroxide for at least 2.5 h in fixative buffer at 20°C. After two washing steps in distilled water, samples were stained with 1% (w/v) uranyl acetate in 20% acetone, dehydrated with a graded acetone series and embedded in Spurr’s low viscosity epoxy resin [50]. For light microscopy, semithin-sections (1–2 μm) were cut with a glass knife (Pyramitome 11800, LKB). Ultrathin-sections (50–70 nm) for transmission electron microscopy were prepared with an ultramicrotome (EM UC6, Leica) and post-stained with aqueous lead citrate (100 mM, pH 13.0). Micrographs were taken at 80 kV with a 268 electron microscope (Fei Morgagni).

### Analysis of Wax and Cutin

The second to fourth internode region of primary inflorescence stalks from 7-week-old plants was used for wax and cutin analyses. For each replicate, stem segments from three to four individual plants were pooled, and samples were provided from two independent harvests of each *FAX1* mutant line and respective wild-type controls. Determination of wax and cutin coverage of stems was essentially carried out as described previously [51,52]. Wax was extracted in chloroform and C24 alkane was added as internal standard. For cutin analysis, exhaustively extracted stems (1:1; methanol:chloroform) were transesterified using methanolic HCl, and cutin monomers were extracted in hexane containing C32 alkane as internal standard. Gas chromatographic and mass spectrometric analysis was carried out after derivatization of extracted wax and cutin with pyridine and BSTFA.

### Measurement of Polar Lipids and Free Fatty Acids

For each independent harvest (2-times for *fax1* knockout, 4-times for *FAX1* over-expressing lines) cauline leaves and flowers (stage 10–15, according to [53]) were sampled from at least ten individual, 7-week-old plants and grinded in liquid nitrogen. To be able to work on tissue of identical sample pools (i.e., from 7-week-old plants) for wax/cutin analysis, FA/lipid determination, and transcript profiling, as well as because FAX1 is highly expressed in cauline leaves (see S5A Fig.), we chose the latter instead of old rosette leaves. Tissue powder of each harvest was portioned into three aliquots of 50 mg, which were used to determine polar lipid and free FA contents. For details on data analysis, see S1 Table. Lipids/FAs were extracted from six (*fax1* k.o) to 12 (*FAX1* over-expressors) biological replicates using 1 ml of a pre-cooled (−20°C) methanol:methyl-tert-butyl-ether (1:3) mixture, spiked with 0.1 μg/ml PC 34:0 (17:0, 17:0) as internal standard. The samples were incubated for 10 min at 4˚C, followed by another 10 min incubation in an ice-cooled ultrasonication bath. After adding 650 μl of UPLC grade water:methanol (3:1), the homogenate was vortexed and centrifuged for 5 min in a table top centrifuge. The addition of water:methanol leads to a phase separation producing an upper organic phase, containing the lipids, and a lower phase containing the polar and semi-polar metabolites. The upper organic phase was removed, dried in a speed-vac concentrator, and re-suspended in 500 μl buffer B (see below) and transferred to a glass vial. 2 μl of this sample were injected onto a C8 reversed phase column (100 mm × 2.1 mm × 1.7 μm particles BEH C8, Waters), using a Waters Acquity UPLC system. The two mobile phases were water (UPLC MS grade, BioSolve) with 1% 1 M NH4Ac, 0.1% acetic acid (buffer A), and acetonitrile:isopropanol (7:3, UPLC grade BioSolve) containing 1% 1 M NH4Ac, 0.1% acetic acid (buffer B). The gradient separation, which was performed at a ﬂow rate of 400 μl/min, was 1 min 45% A, 3 min linear gradient from 45% A to 35% A, 8 min linear gradient from 25 to 11% A, 3 min linear gradient from 11% A to 1% A. After washing the column for 3 min with 1% A the buffer was set back to 45% A and the column was re-equilibrated for 4 min (22 min total run time). Mass spectra were acquired as described [23,24,54] using either an Orbitrap Exactive mass spectrometer (Thermo-Fisher) for *fax1* knockout lines or a Waters Synapt G1 (Waters) for *FAX1* over-expressors, and corresponding wild types, respectively. The spectra were recorded using altering full scan and all-ion fragmentation scan mode, covering a mass range from 100–1,500 m/z. The resolution was set to 10,000 with 10 scans per second. Spectra were recorded from min 0 to min 20 of the UPLC gradients. The analysis of the spectra (alignment, peal picking, normalization and peak integration) was performed with the software package CoMet 2.0 (Nonlinear Dynamics) according to the instructions of the vendor.

### Complementation of FA Uptake in Yeast

For growth assays in yeast, the coding sequence of the mature At-FAX1 protein was subcloned into the yeast expression plasmid pDR195 (XhoI/BamHI). Therefore, we fused the open reading frame of the predicted mature At-FAX1, starting with aa 34 of the preprotein, behind an “ATG” base triplet by PCR amplification. The yeast mutant strains *fat1* (LS2020-YB332) and *faa1/faa4* (LS1849-YB525) are specified in [26]. Both strains were transformed with mature At-FAX1/pDR195 and the vector control pDR195 as described [45]. If not denoted elsewhere, liquid cultures of the respective yeast cells were grown to exponential phase in synthetic defined medium (SD-ura), containing 0.1% (w/v) glucose, 0.7% (w/v) yeast nitrogen base without amino acids, and necessary auxotrophic amino acids without uracil. Subsequently, 2 μl drops of the cultures were spotted in different dilutions onto SD-ura plates (2% agar), supplemented with 3.6 mM α-linolenic acid (0.1%, w/v in ethanol), and 1% tergitol (to increase α-linolenic acid solubility). For control plates, an equal amount of the solvent ethanol was added instead of α-linolenic acid. Assays in the presence of cerulenin were performed according to [26,27] in SD-ura media supplemented with 2% (w/v) glucose, 0.5% Brij 58, 0.7% KH_2_PO_4_, 10μM cerulenin and either 100μM of palmitic, stearic, oleic or α-linolenic acid. Growth of yeast cells on solid media was documented between 2 to 6 days at 30°C. OD_600_ measurements were performed in identical liquid media, inoculated to a starting OD_600_ of 0.05 or 0.06/0.03 for cerulenin experiments with the respective yeast cells. Cultures were continuously shaken at 30°C and the OD at 600nm was determined at indicated time points.

### DNA Microarray Analysis

Tissue from flowers (stage 10–15 according to [53], compare S3 Fig.) and from the second to fourth internode of primary inflorescent stalks for each harvest was pooled from more than ten individual, 7-week-old plants (identical sample pool for lipid analysis) and used for preparation of RNA samples by the Plant RNeasy Extraction kit (Qiagen). RNA (200 ng) of four or five independently harvested samples (*n* = 4–5) from wild type (Col-0 and WT2, segregated from heterozygous *fax1–2*), *fax1* knockout (*fax1–1* and *fax1–2* lines) as well as *FAX1* over-expressors (ox#2 and ox#4 lines) was processed and hybridized to Affymetrix GeneChip *Arabidopsis* ATH1 Genome Arrays using the Affymetrix 3´-IVT Express and Hybridisation Wash and Stain kits (Affymetrix, High Wycombe, UK) according to the manufacturer’s instructions. Raw signal intensity values (CEL files) were computed from the scanned array images using the Affymetrix GeneChip Command Console 3.0. For quality check and normalization, the raw intensity values were processed with Robin software [55] default settings as described [19]. Specifically, for background correction, the robust multiarray average normalization method [56] was performed across all arrays (between-array method). Statistical analysis of differential gene expression of mutant versus wild-type samples was carried out using the linear model-based approach developed by [57]. In total, we analyzed the following comparisons (see S7 Fig.): (A) flowers: *fax1* knockout (*n* = 5) versus wild type (*n* = 5); (B) flowers: *FAX1* over-expressors (*n* = 8, four times each ox#2, ox#4) versus wild type (*n* = 5); (C) stems: *fax1* knockout (*n* = 4) versus wild type (*n* = 4). The obtained *p* values were corrected for multiple testing using the nestedF procedure, applying a significance threshold of 0.05 in combination with the Benjamini and Hochberg false-discovery rate control [58]. All microarray data are available in the ArrayExpress database (www.ebi.ac.uk/arrayexpress) under accession number E-MTAB-3090.

### Structural Modeling

Structural models of At-FAX1 and At-FAX6 were generated by Phyre^2^ [59], based on alignments with the PDB entries for human TMEM14C (c2losA) and TMEM14A (c2lopA), respectively. Identity of At-FAX1 with its template TMEM14C was 21% and for At-FAX6 with TMEM14A 36%, while confidence of both models was 99.9%, thereby indicating a high confidence and accuracy of the core models. Structural alignments were created with PyMOL [60].

## Supporting Information

S1 Data Supporting numerical data. (XLSX)

S1 Fig Mutation of *FAX1* in Arabidopsis. (A) Schematic representation of the *At-FAX1* gene (At3g57280). Black arrows indicate six exons, white lines represent introns. Two T-DNA insertion sites in the first intron (*fax1–1*, position +526) and in the first exon (*fax1–2*, position +388–405, including a 17bp deletion of *FAX1*) are indicated by triangles. T-DNAs are pCSA110 in the SAIL_66_B09 line (*fax1–1*) and pAC161 in the GABI-Kat line 599E01 (*fax1–2*), respectively. Binding sites for *FAX1* gene-specific primers and T-DNA specific left border (LB) primers used for PCR genotyping and for RT-PCR are depicted. +1: predicted transcriptional start. (B) RT-PCR analysis of the *FAX1* transcript content in leaves and flowers of homozygous *fax1–1*, *fax1–2* knockout lines, Col-0 wild type, and wild type segregated from heterozygous *fax1–2* line (wt2). RNA was prepared from 7-week-old plants and reverse transcribed into cDNA [45]. PCR reactions were conducted with gene-specific primers for *FAX1* (LCfw and LCrev, 265 bp product on wild-type cDNA). For primer positions, see (A). As control, constitutively expressed actin 2/8 (PCR product of 435 bp) was analyzed. (C) Quantitative real-time RT-PCR was performed as described [45] on RNA, isolated from 14-day-old seedlings of *FAX1* wild type (Col-0 and wt2), *fax1–2* complementation (Co#7, Co#54), and *FAX1* overexpressing (ox#2, ox#4) lines. The transcript content was quantified relative to 10,000 molecules of actin 2/8 mRNA (*n* = 3; ±SD) and normalized to the amount in Col-0, which was set to 1.0 (for numerical values, see S1C Data). Please note that the *y*-axis for ox#4 (right) is scaled up 10-fold. (D) Immunoblot of At-FAX1 on total protein extracts isolated from leaf material of 30-day-old *fax1–2*, Col-0, Co#54, ox#2, ox#4 plants (see [C]). Please note that for detection of signals in all samples, different amounts of protein were loaded: 80, 80, 40, 80, and 5μg, respectively. Antiserum against the inner envelope protein TIC62 was used as loading control. For comparison, purified inner envelope membranes from pea (IE, 40μg protein) were stained with Ps-FAX1 antiserum. Numbers indicate the molecular mass of proteins in kDa. Please note that the band at 25kDa represents the main signal for FAX1 in all samples (compare Fig. 2D). However, around 23kDa a second band becomes visible, when a high amount of protein is loaded (triangles). When FAX1 is overexpressed (Co#54, ox#4), this band represents a strong signal and therefore most likely corresponds to FAX1 proteins with a more packed conformation. In addition, several other signals appear in FAX1 overexpression lines (asterisks). All bands are absent in *fax1–2* knockout leaves and can thus be regarded as specific for FAX1.
(TIF)

S2 Fig Stem tissues of *FAX1* mutants. Cross-sections and vascular tissue of primary inflorescence stems (bottom part of second internode) from 5-week-old homozygous *fax1–2* knockout [(A), (D), (G)], Col-0 wild-type [(B), (E), (H)] and the FAX1 over-expressor ox#2 [(C), (F), (I)]. (A), (B), (C) Overview of stem cross sections (light microscopy, bar = 100 μm). (D), (E), (F) Close-up of sclerenchyma/phloem (left) and xylem (right) (light microscopy, bar = 25 μm). (G), (H), (I) Cell walls of tracheids in xylem tissue (TEM, bar = 5 μm). h: hypodermis; p: phloem; s: sclerenchyma; x: xylem. Please note that FAX1ox#2 stems are characterized by an increased amount of xylem and phloem vessels as well as by a multi-layered procambium as depicted by arrowheads in (C) and (F).
(TIF)

S3 Fig FAX1 function is essential for male fertility and pollen cell wall assembly. Pictures of flowers, anthers, and mature pollen of 5-week-old *fax1–1* knockout, WT2 wild-type, complementation lines Co#7, Co#54, and *FAX1* over-expressors ox#2, ox#4. (A), (C), (E) Development of flower buds and young siliques. Brackets indicate flower stages 10–15 [53] used for FA/lipid and microarray analysis. (B), (D), (F) Close-up of opened flowers. Arrowheads: non- or weakly pollinated stigma in *fax1–1* (B) and Co#7, Co#54 (D), respectively; arrows: anthers with released pollen in WT2, ox#2, and ox#4; white circles: colorless pollen grains, released by Co#54 anther. (G) Close-up of dehiscent anthers. Please note that while *fax1* k.o. anthers do not release pollen grains, Co#7 and Co#54 anthers produce few and colorless (white circles), and Col-0 wild-type generate many, yellow pollen, respectively. (H) Cross section of mature, dehisced anther of line Co#54 (light microscopy, bar = 50 μm). The appearance of Co#54 anthers is intermediate to that of *fax1–2* and Col-0 (compare Fig. 4C). White arrowheads indicate that still some debris material is sticking to the pollen grain/endothecium boundary. en: endothecium cells of anthers. (I), (J), (K) TEM pictures of anther cell/pollen grain intersections in Co#54 (I, bar = 5 μm; J, bar = 1μm) and pollen cell wall (J, bar = 500 nm) at mature tricellular pollen stages. Please note that still some debris material is sticking to pollen grains (white arrowheads) and that in comparison to wild type (see Fig. 4E, F), the pollen exine is not fully established. For example, tectum structures seem to be absent, and the trypine pollen coat is not correctly assembled. en: endothecium cell; e: exine layer with e_b_: bacula structures; e_n_: nexine layer (black arrowheads), i: intine layer; po: cytosol of pollen grain; try: tryphine pollen coat.
(TIF)

S4 Fig FA-transport specificity of FAX1 in yeast. The mature At-FAX1 cDNA in pDR195 (FAX1) and the empty plasmid pDR195 (-) were introduced into *fat1* and *faa1/faa4* yeast mutants, respectively (compare Fig. 8). Growth assays were performed in the presence of 5–10 μM cerulenin (CER, inhibitor of FA-biosynthesis) and 100μM FAs according to [26,27]. To test for FAs, which in planta have to be exported from chloroplasts (see [1]), we used palmitic acid (PAL, C_16:0_), stearic acid (STE, C_18:0_), and oleic acid (OLE, C_18:1_). For results with the control α-linolenic acid (LIN, C_18:3_), which in vivo is not exported from plastids, see (B), right panel, and Fig. 8D. (A), (B) For growth on solid medium, 2 μl of exponentially growing yeast cells (diluted to an OD_600_ of 0.1/ml) were grown at 30°C on SD-ura plates (2% glucose, 0.5% Brij 58, 0.7% KH_2_PO_4_) in the presence of 10μM (A) and 5μM (B) CER, respectively. Cartoon: distribution of different strains on plates. Whereas *faa1/faa4* mutants (on lower halves of plates) did not grow at all, *fat1* cells transformed with the empty plasmid (-, upper right quarter) showed colonies in all assays after four to six days of incubation. In contrast, growth of *fat1* with FAX1 (upper left quarter on plates with 10μM CER in [A]) was strongly restricted in the presence of PAL (left) and reduced with STE (middle) and OLE (right panel). In the presence of 5μM CER (B), growth inhibition by FAX1 was not as strong but still differential, resulting in an OD_600_/ml of all cells grown in the upper left quarter of 2.5 (for PAL), 3.6 (for STE), 4.0 (for OLE, see S1D Data), and 4.5 (for LIN), respectively. (C), (D) Growth of *fat1* cells in liquid SD-ura with 10μM CER [see (A)]. White circles and black triangles: growth of pDR195 (-) and matFAX1/pDR195 (FAX1) cells. Red bars and numbers indicate maximal difference of cell density ratios, for numerical values see S1D Data. As on plates in (A) and (B), liquid cultures of *faa1/faa4* cells did not grow (see S1D Data). (C) Cell growth at 30°C was started with an OD_600_ of 0.06 from exponentially growing cultures and was monitored from 15 h (all cells at OD_600_ 0.2) until 45 h (left) and 65 h (middle, right graphs). In general, growth curves reflected behavior on plates in (A). Whereas growth of *fat1* (FAX1, black triangles) in comparison to *fat1* (-, white circles) was strongly inhibited by PAL (left), reduction of cell amplification in the presence of STE (middle) and OLE (right) was less. Please note different scaling of *y*-axes. For comparison to LIN, see Fig. 8D (please note that in the latter assay growth of all cells was delayed, when compared to curves in [C]). (D) Growth curves of slowly amplifying *fat1* cells. In contrast to (C), cell growth was started with an OD_600_ of 0.03 from non-exponentially growing cultures and monitored for 60 h incubation at 30°C. Please note that in slow-growing, diluted cell suspensions, FAX1 (black triangles) can complement for the lack of FAs. Here, the strongest growth promotion was with OLE (3.9-fold), followed by PAL (2.9-fold) and STE (2.5-fold). Without CER—i.e. no inhibition of intrinsic FA-synthesis—cells entered a logarithmic, rapid growth phase between 15–20 h, and differences in cell density were only marginal (<1.5), except for OLE between 14–20 h, which reflected differences in cell density ratios at 60 h in the presence of CER (S1D Data). **FA-transport specificity of FAX1 in yeast**: Due to the results depicted in (A)–(D), we conclude that in yeast, FAX1 is able to transport all FAs that are exported from chloroplasts in planta, but prefers palmitic (C_16:0_) over oleic (C_18:1_) and stearic acid (C_18:0_). Between the latter two, however, we were unable to distinguish specificity. FAX1 transport of α-linolenic acid (C_18:3_), a polyunsaturated FA, which in planta has not been described to be exported from chloroplasts, was in the range of oleic/stearic acid but significantly lower than for palmitic acid (compare Fig. 8D). **Discussion**: In general, CER inhibits FA-biosynthesis, and thereby growth of yeast cells becomes dependent on FA-uptake from the extracellular medium. Depending whether we used slow or rapidly growing yeast cells, we found opposite effects for FAX1. (**1**.) In slow-growing *fat1* cells (D), FAX1 could partly complement growth defects in the presence of CER, most likely by mediating uptake of FAs (assay for yeast Fat1, compare Fig. 2 in [27]). Without FAX1, in contrast, growth inhibition of *fat1* (-) cells was strong over the time course of the experiment. The growth promoting effect of FAX1 was highest for oleic acid (C_18:1_), followed by palmitic (C_16:0_) and stearic acid (C_18:0_), and thus exactly reflecting the in planta situation (see below). Although FAX1 most likely is not directly linked to yeast endogenous FA-activation and metabolism (see below), strong inhibition of FA-synthesis by CER in highly diluted, slow-growing cell cultures most likely pushes free FAs transported by FAX1 into yeast intrinsic FA-metabolism pathways. (**2**.) In long-term growth of rapidly amplifying *fat1* cells (A, B, and C), however, FAX1 was inducing growth inhibition that was strongest for palmitic acid (C_16:0_), followed by stearic (C_18:0_), oleic acid (C_18:1_), and the control α-linolenic acid (C_18:3_). We therefore conclude that the presence of FAX1 during prolonged incubation of these cells leads to an accumulation of toxic free FAs and in consequence growth arrest as observed for an excess of α-linolenic acid (see Fig. 8A–C). Since FAX1—unlike the yeast Fat1 protein—probably is not directly interacting and co-operating with the endogenous acyl-CoA synthetases for intracellular FA-activation (Faa1p or Faa4p), extended FAX1 FA-uptake most likely leads to a surplus of toxic free FAs, which can’t be efficiently esterified to CoA for subsequent metabolism. In contrast, *fat1* cells without FAX1 (transformed with empty vector control) were able to grow after 6 days on plate (A), (B) and about 24 h in liquid culture (C). This late growth activation can be explained by a decline of FA-biosynthesis inhibition by CER at high cell densities, presumably in combination with a bypass of FA-uptake either by yeast intrinsic transporters and/or passive diffusion through membranes. Both hypotheses are supported by the following findings: (i) no growth of *fat1* (-) at low cell densities (strong CER effect, see above); (ii) less pronounced differences in growth promotion/inhibition with 5μM CER (B) or 50μM of FAs (see S1D Data); (iii) no growth of *faa1/faa4* cells in presence of CER, due to the lack of FA-activation for subsequent metabolism. In summary, the observed growth effects are nontrivial, because of simultaneous interference with yeast FA-biosynthesis (CER), FA-transport, and FA-activation (*fat1*, *faa1/faa4* mutants). Furthermore, toxicity effects of accumulating free PAL, STE, OLE or LIN might be diverse. However, we can show reproducible and specific results for FAX1 and define a substrate specificity range. According to acyl-ACP synthesis rates and specificity of thioesterases in *Arabidopsis* chloroplasts, oleic acid (C_18:1_) is the major FA exported, followed by palmitic acid (C_16:0_) and only little amounts of stearic acid (C_18:0_; compare [1,33,34]). FAX1 in our yeast assays preferred FA 16:0 over 18:1 and 18:0 and thus most likely mainly is involved in the plastid export of palmitic acid. Because these observations were made in vivo, but in a heterologous and nontrivial system, we can, however, only approach the in planta situation.
(TIF)

S5 Fig Expression of plastid *At-FAX* genes throughout development. Expression profiles of *Arabidopsis FAX1*, *FAX2*, *FAX3*, *FAX4* (green, light green, orange, light orange bars, respectively) during development. Data used to create digital Northern blots are based on DNA microarray analyses obtained from AtGenExpress developmental series (A–E; [61]), pollen development arrays (C; [62]), and from different seed microarray analyses (E; [63,64]). If not denoted elsewhere, the ecotype is Col-0. Mean signal intensities in (A)–(E) were averaged from two to three replicates (arbitrary units ± SD, for numerical values see S1E Data). Since expression data for *FAX1–4* in seed tissue is generally high but differs between experiments and ecotypes (see also S6 Fig.), we show representative data in (D) and (E): (i) In general, *FAX1* expression in seeds is low when compared to all other plastid *FAX* genes (see D, E, S6A Fig.). (ii) In contrast, *FAX3* expression is quite strong in mature and dry seeds (see D, E). (iii) However, upon imbibition in aqueous solutions, expression of *FAX2* and *FAX4* is induced as well (see E, S6A Fig.), so that upon germination most likely FAX2, FAX3, and FAX4 are predominant. (iv) Whereas *FAX2* and *FAX3* are expressed in seed coat, endosperm and embryo of mature seeds, *FAX1* and *FAX4* transcripts are absent in seed coats (see S6A Fig.). (A), (B) Developmental series. Tissues and organs are specified as follows, age of plants in days grown in continuous light is indicated in brackets. (A) hy: hypocotyl (7); co: cotyledon (7); rl: rosette leaf no. 10 (17); sl: senescing leaf (35); cl: cauline leaf (21+); sa: shoot apex, before bolting (14); st: stem, second internode (21+); ro: root (17). (B) Dissected mature, open flowers (21+), stage 15 according to [53]. fl: total flower; ped: pedicel; sep: sepal; pet: petal; sta: stamen; car: carpel. (C) Pollen development. unm: uninucleate microspore; bcp: bicellular pollen; tcp: tricellular pollen; mp: mature pollen grain (mp data points from AtGenExpress). (D) Embryo and seed development. Seed stages 3–10 are defined according to embryo development as follows: 3: mid-globular to early heart; 4: early heart to late heart; 5: late heart to mid-torpedo; 6: mid-torpedo to late torpedo; 7: late torpedo to early walking stick; 8: walking stick to early curled cotyledons; 9: curled cotyledons to early green cotyledons; 10: green cotyledons. Note that stages 3–5 include silique tissue. (E) Comparison of dry seeds and seeds imbibed in water for 6 h or 24 h to induce germination. AtGenExpress data (left); micorarray data, according to [64] (middle); dissected endosperm (end) and embryo (emb) from Ler ecotype seeds [63] (right).
(TIF)

S6 Fig Expression of plastid *At-FAX* genes in seed tissues. (A) Expression of *FAX1–4* in tissues of linear cotyledon (lc) and maturation green (mg) stage embryos in late seed development (Harada-Goldberg dataset of laser capture microdissected seeds: “Gene Networks in Seed Development”). Please note that during fixation, tissue was submerged in aqueous solutions and therefore transcript levels of *FAX2* and *FAX4* might resemble those of imbibed tissue in S5E Fig. CZE, chalazal endosperm; CZSC, chalazal seed coat; EP, embryo proper; GSC, general seed coat; MCE, micropylar endosperm; PEN, peripheral endosperm; S, suspensor. Tissues are colored according to transcript density for signals that are absent (white), insufficient (blue), <500 (beige), 500–5,000 (orange), 5,000–10,000 (purple), and >10,000 (dark red). Highest expression in mature seed tissue is indicated. Data is available at http://estdb.biology.ucla.edu/seed/. (B) Seed anatomy series from the genevestigator database [65]. Please note that expression from large sets of samples including different ecotypes and experimental setups is depicted as boxplots of log2 values. Data is available at https://www.genevestigator.com/gv/.
(TIF)

S7 Fig Differential gene expression in FAX1 mutants. Venn diagram summarizing numbers and overlaps of significantly regulated genes (*p*-value ≤ 0.05) from DNA microarray analysis (ATH1 GeneChip) of FAX1 mutants in flower and stem tissues (see E-MTAB-3090 at www.ebi.ac.uk/arrayexpress). (A) Comparison *fax1* knockout (*n* = 5) versus wild type (*n* = 5) in flower tissue. Of 3346 differentially regulated genes, 1676 were significantly up-regulated, whereas 1670 were down-regulated in *fax1* knockout flowers. (B) Comparison *FAX1* over-expressors (*n* = 8, 4 times each line ox#2, ox#4) versus wild type (*n* = 5) in flower tissue. In flowers of *FAX1* over-expressors 2366 genes showed to be significantly regulated (964 up, 1402 down). (C) Comparison *fax1* knockout (*n* = 4) versus wild type (*n* = 4) in stem tissue. Of 1967 differentially regulated genes, 1335 were significantly up-regulated, whereas 632 were down-regulated in *fax1* knockout stems.
(TIF)

S8 Fig Differential gene expression in *fax1* knockout versus wild type flowers. Results of DNA microarray analysis (ATH1 GeneChip) for the comparisons depicted in S7A Fig.: *fax1* knockout (*n* = 5) versus wild type (*n* = 5) in flower tissue. For better visualization, we sub-divided TAIR10 functional categories (Ath_AFFY_ATH1_TAIR10_Aug2012; http://mapman.gabipd.org) into portions containing between 50–600 genes. Furthermore, we displayed only those categories containing more than 10% of significantly regulated genes (*p*-value ≤ 0.05), respectively (see S1F Data for numerical values). The complete microarray data are available in the ArrayExpress database (www.ebi.ac.uk/arrayexpress) under accession number E-MTAB-3090. Depicted functional categories are as follows: 1. photosynthesis; 2. major CHO metabolism; 4. glycolysis; 10. cell wall; 26.3. gluco-, galacto- and mannosidases; 26.18. invertase + pectin methylesterase inhibitor family; 8. TCA cycle / org transformation; 9. mitochondrial electron transport; 13. amino acid metabolism; 15. metal handling; 16. secondary metabolism; 17. hormone metabolism; 18. Co-factor and vitamin metabolism; 21. redox; 23. nucleotide metabolism; 26.28. GDSL-motif lipases; 27.2. transcription; 27.3.6. regulation of transcription: bHLH, Basic Helix-Loop-Helix family; 27.3.24. regulation of transcription: MADS box transcription factor family; 29.1./2. protein aa activation/protein synthesis; 29.4. protein postranslational modification; 29.6. protein folding; 33.1./2. development: storage/late embryogenesis abundant proteins; 30.3. signalling calcium; 30.4. signalling phosphoinositides; 34. transport.
(TIF)

S9 Fig Differential gene expression in *FAX1* over-expressors versus wild type flowers. Results of DNA microarray analysis (ATH1 GeneChip) for the comparisons depicted in S7B Fig.: *FAX1* over-expressors (*n* = 8, 4 times each line ox#2, ox#4) versus wild type (*n* = 5) in flower tissue. For better visualization, we sub-divided TAIR10 functional categories (Ath_AFFY_ATH1_TAIR10_Aug2012; http://mapman.gabipd.org) into portions containing between 50–600 genes. Furthermore, we displayed only those categories containing more than 7.5% of significantly regulated genes (*p*-value ≤ 0.05), respectively (see S1F Data for numerical values). The complete microarray data are available in the ArrayExpress database (www.ebi.ac.uk/arrayexpress) under accession number E-MTAB-3090. Depicted functional categories are as follows: 1. photosynthesis; 2. major CHO metabolism; 4. glycolysis: cytosolic branch; 10. cell wall; 26.3 gluco-, galacto- and mannosidases; 26.4 beta 1,3 glucan hydrolases; 26.18 invertase + pectin methylesterase inhibitor family protein; 31.4 cell: vesicle transport; 9. mitochondrial electron transport; 16.5 secondary metabolism: sulfur-containing.glucosinolates; 16.8 secondary metabolism: flavonoids; 17.1 hormone metabolism: abscisic acid; 17.2 hormone metabolism: auxin; 17.3 hormone metabolism: brassinosteroid; 17.6 hormone metabolism: gibberelin; 20.2 stress abiotic; 26.7 oxidases—copper, flavone etc.; 26.8 nitrilases, nitrile lyases, berberine bridge enzymes, reticuline oxidases, troponine reductases; 26.10 cytochrome P450; 26.12 peroxidases; 26.13 acid and other phosphatases; 26.21 protease inhibitor/seed storage/lipid transfer protein (LTP) family protein; 26.28 GDSL-motif lipase; 27.2 transcription; 27.3.22 regulation of transcription: HB, Homeobox transcription factor family; 27.3.24 regulation of transcription: MADS box transcription factor family; 29.1 protein aa activation; 29.2.11 protein synthesis: ribosomal protein.prokaryotic; 29.2.2 protein synthesis: ribosome biogenesis; 29.4.1 protein: postranslational modification.kinase; 29.5.1 protein: degradation.subtilases; 29.5.3/4/5 protein: degradation Cys/Asp/Ser protease; 29.6 protein folding; 33.1/2 development: storage proteins/late embryogenesis abundant; 30.2 signalling: receptor kinases; 30.3 signalling: calcium; 30.5 signalling: G-proteins; 30.8 signalling: RALF genes; 34 transport; 35.1.40 glycine rich proteins; 35.1.41/42 hydroxyproline + proline rich protein family.
(TIF)

S10 Fig Differential gene expression in *fax1* knockout versus wild type stems. Results of DNA microarray analysis (ATH1 GeneChip) for the comparisons depicted in S7C Fig.: *fax1* knockout (*n* = 4) versus wild type (*n* = 4) in stem tissue. For better visualization, we sub-divided TAIR10 functional categories (Ath_AFFY_ATH1_TAIR10_Aug2012; http://mapman.gabipd.org) into portions containing between 50–600 genes. Furthermore, we displayed only those categories containing more than 7.5% of significantly regulated genes (*p*-value ≤ 0.05), respectively (see S1F Data for numerical values). The complete microarray data are available in the ArrayExpress database (www.ebi.ac.uk/arrayexpress) under accession number E-MTAB-3090. Depicted functional categories are as follows: 3. minor CHO metabolism; 4. glycolysis; 10. cell wall; 26.2 UDP glucosyl and glucoronyl transferases; 26.3 gluco-, galacto- and mannosidases; 26.4 beta 1,3 glucan hydrolases; 26.18 invertase + pectin methylesterase inhibitor family; 31.1 cell organisation; 31.2 cell division; 31.3 cell cycle; 31.4 cell: vesicle transport; 16.2 secondary metabolism: phenypropanoids; 16.5 secondary metabolism: sulfur-containing glucosinolates; 17.2 hormone metabolism: auxin; 17.3 hormone metabolism: brassinosteroid; 20.2 stress abiotic; 23.3 nucleotide metabolism: salvage; 26.21 protease inhibitor/seed storage/lipid transfer protein (LTP) family protein; 28.1.3 DNA synthesis: chromatin structure.histone; 28.2 DNA repair; 29.3 protein targeting; 29.4.1 protein: postranslational modification.kinase; 30.2 receptor kinases; 30.3 signalling calcium; 34.3 transport: amino acids/ammonium; 35.1.41/42 hydroxyproline + proline rich protein family.
(TIF)

S1 Table Fatty acid and polar lipid contents in *FAX1* mutants. Free fatty acid (FA) and polar lipid species were determined in flowers and caulinary leaves of 7-week-old, mature flowering plants. Data (arbitrary units) are expressed as ratios to the internal standard (PC 34:0) and normalized to mg fresh weight (FW). Significantly different *p-*values (Student’s *t*-test) for comparisons of FAX1 mutants versus wild type (wt) are indicated by orange (*p* < 0.05) and light blue (*p* < 0.01) background, respectively. Numerical values depicted in Fig. 6 and Fig. 7, are highlighted by green and purple background, respectively. Since for *fax1* knockout lines (ko; Orbitrap MS, [23]), measurements were conducted with a different mass spectrometer than for FAX1 over-expressors (ox; qTOF MS, [24]), different scaling of the relative values is obtained. Data analysis details: For *fax1* knockouts, analysis was performed on tissue from two independent *fax1–1*, *fax1–2* knockout and Col-0, WT2 wild-type lines, separately harvested for two times. For FAX1 over-expressors the two independent lines ox#2 and ox#4 were compared to Col-0 wild type in four different harvests. For each harvest, cauline leaves and flowers were sampled from at least ten individual plants and tissue powder was portioned into three aliquots. In total this resulted in *n* = 12 data points for each molecule species, determined in each genotype (*fax1* knockout, FAX1 over-expressor, and wild types). Significance analysis of mutant to wild-type differences was performed over all harvests and lines in each tissue. Only those changes that showed to be significant (*p*-value < 0.05) in both lines and all harvests were selected to be robust, thereby covering a pool of 8–12 single data points. Few exceptions are indicated and highlighted in yellow. Whereas for *fax1* knockout lines, mean values, averaged over *fax1–1*, *fax1–2*, and Col-0, WT2 wild type, repectively, (*n* = 4–6 ± SD) for one harvest are shown, mean data for over-expressors represent those of line ox#4 and the wild type Col-0 (*n* = 5–12 ± SD), averaged over four independent harvests.
(XLSX)

S2 Table Plastid FAX1 impacts cellular FA/lipid homeostasis in leaves. Content (mol %) of free FAs and polar lipids, determined in caulinary leaves of 7-week-old, mature plants. Please note that only species significantly different in *FAX1* mutants (mu) compared to wild type (wt) are depicted. For a complete dataset, details on samples, and significance analysis see S1 Table. Samples and subdivision into different species (A–D) are identical to Fig. 6. Numbers in subheadings indicate significantly different species versus all molecules measured (see S1 Table). The direction of changes (↑: up; ↓: down), the fold change (FCH), and the differences of mol% in *FAX1* mutants versus wild type are given. Asterisks label the two most abundant species of each molecule class determined (compare S1 Table). DGDG: digalactosyl-diacylglycerol; FA: free fatty acid; MGDG: monogalactosyl-diacylglycerol; PC: phosphatidyl-choline; PE: phosphatidyl-ethanolamine; PG: phosphatidyl-glycerol; PI: phosphatidyl-inositol; SQDG: sulphoquinovosyl-diacylglycerol.
(DOCX)

S3 Table Plastid FAX1 impacts cellular FA/lipid homeostasis in flowers. Content (mol %) of free FAs and polar lipids, determined in flower tissue of 7-week-old, mature plants. Please note that only species significantly different in *FAX1* mutants (mu) compared to wild type (wt) are depicted. For a complete dataset, details on samples and significance analysis see S1 Table. Subdivision into different species (A–D) is similar to Fig. 6; numbers in subheadings indicate significantly different species versus all molecules measured (see S1 Table). The direction of changes (↑: up; ↓: down), the fold change (FCH), and the differences of mol% in *FAX1* mutants versus wildtype are given. Asterisks label the two most abundant species of each molecule class determined (compare S1 Table). DGDG: digalactosyl-diacylglycerol; FA: free fatty acid; MGDG: monogalactosyl-diacylglycerol; PC: phosphatidyl-choline; PE: phosphatidyl-ethanolamine; PG: phosphatidyl-glycerol; PI: phosphatidyl-inositol; SQDG: sulphoquinovosyl-diacylglycerol.
(DOCX)

S4 Table Plastid FAX1 impacts TAG storage lipid homeostasis. Content (mol %) of triacylglycerol (TAG) oils, determined in tissues of 7-week-old, mature flowering plants. Please note that only species significantly different in *FAX1* mutants (mu) compared to wild type (wt) are depicted. For a complete dataset, details on samples, and significance analysis see S1 Table. Samples and subdivision into (A–D) are identical to Fig. 7. Numbers in subheadings indicate significantly different species versus all molecules determined (see S1 Table). The direction of changes (↑: up; ↓: down), the fold change (FCH), and the differences of mol% in *FAX1* mutants versus wild type are given. Asterisks label the five most abundant species of all TAGs measured (compare S1 Table).
(DOCX)

S5 Table Genes of acyl lipid metabolism, simultaneously regulated in flowers of *FAX1* knockouts and over-expressors. Depicted are 64 genes (plus two with strongest changes), which according to DNA micorarray analysis are significantly regulated in flower tissue of both *fax1* knockout and *FAX1* over-expressor lines, and represent genes of acyl lipid metabolism (ARALIP database; http://aralip.plantbiology.msu.edu/; see [1]). Genes also regulated in stems of *fax1* knockouts (see S6 Table) are boxed and underlined. Arabidopsis Genome Initiative (AGI) codes and the average scaled signals of mutant and wild type, as well as the fold change (FCH) in flowers of *FAX1* knockouts (ko) and over-expressors (ox) are given. Annotation of acyl lipid pathway, protein family, and gene names is according to the ARALIP database. nr: not significantly regulated
(DOCX)

S6 Table Genes of acyl lipid metabolism, regulated in stem tissue of *fax1* knockout mutants. Depicted are 58 genes, which according to DNA micorarray analysis are significantly regulated in stem tissue of *fax1* knockout mutants, and represent genes of acyl lipid metabolism (ARALIP database; http://aralip.plantbiology.msu.edu/; see [1]). Genes also regulated in FAX1 mutant flowers (see S5 Table) are boxed and underlined. Arabidopsis Genome Initiative (AGI) codes and the average scaled signals of mutant and wild type as well as the fold change (FCH) in flowers of *fax1* knockouts (ko) are given. Annotation of acyl lipid pathway, protein family and gene names is according to the ARALIP database.
(DOCX)

S7 Table Oligonucleotides used in this study. (DOCX)

S1 Text Differential gene expression in FAX1 mutants. (DOCX)

S2 Text The role of FAX1 in male flower tissue. (DOCX)

## Abbreviations

ABC: ATP-binding cassette
ACP: acyl carrier protein
ACS: acyl-CoA synthetase
AGI: Arabidopsis genome initiative
CoA: coenzyme A
DGDG: digalactosyl-diacylglycerol
ER: endoplasmatic reticulum
FA: fatty acid
FAX1: fatty acid export 1
FW: fresh weight
GFP: green fluorescent protein
IE: inner envelope of chloroplasts
LACS: long-chain acyl-CoA synthetase
MGDG: monogalactosyl-diacylglycerol
NMR: nuclear magnetic resonance
OE: outer envelope of chloroplasts
PA: phosphatidic acid
PC: phosphatidyl-choline
PE: phosphatidyl-ethanolamine
PG: phosphatidyl-glycerol
PI: phosphatidyl-inositol
RT-PCR: reverse transcriptase-polymerase chain reaction
SQDG: sulphoquinovosyl-diacylglycerol
TAG: triacylglcerol
TEM: transmission electron microscopy
UTR: untranslated region
