# Supplementary figures and images for: FAX1, a Novel Membrane Protein Mediating Plastid Fatty Acid Export
Source: PLoS Biol. 2015 Feb 3;13(2):e1002053. doi: 10.1371/journal.pbio.1002053 (PMC4344464; doi:10.1371/journal.pbio.1002053)

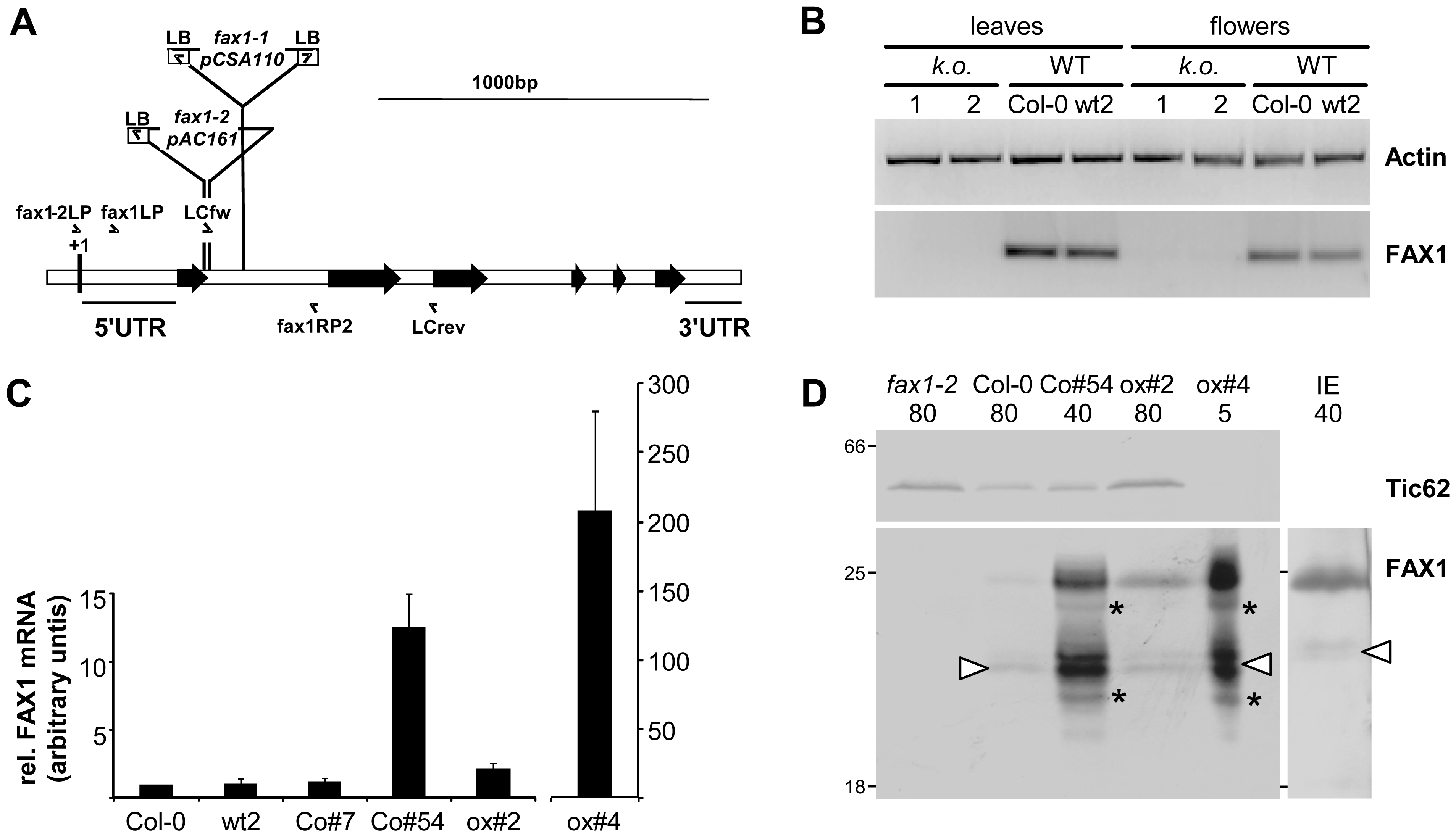

Supplement: S1 Fig — (A) Schematic representation of the At-FAX1 gene (At3g57280). Black arrows indicate six exons, white lines represent introns. Two T-DNA insertion sites in the first intron (fax1–1, position +526) and in the first exon (fax1–2, position +388–405, including a 17bp deletion of FAX1) are indicated by triangles. T-DNAs are pCSA110 in the SAIL_66_B09 line (fax1–1) and pAC161 in the GABI-Kat line 599E01 (fax1–2), respectively. Binding sites for FAX1 gene-specific primers and T-DNA specific left border (LB) primers used for PCR genotyping and for RT-PCR are depicted. +1: predicted transcriptional start. (B) RT-PCR analysis of the FAX1 transcript content in leaves and flowers of homozygous fax1–1, fax1–2 knockout lines, Col-0 wild type, and wild type segregated from heterozygous fax1–2 line (wt2). RNA was prepared from 7-week-old plants and reverse transcribed into cDNA [45]. PCR reactions were conducted with gene-specific primers for FAX1 (LCfw and LCrev, 265 bp product on wild-type cDNA). For primer positions, see (A). As control, constitutively expressed actin 2/8 (PCR product of 435 bp) was analyzed. (C) Quantitative real-time RT-PCR was performed as described [45] on RNA, isolated from 14-day-old seedlings of FAX1 wild type (Col-0 and wt2), fax1–2 complementation (Co#7, Co#54), and FAX1 overexpressing (ox#2, ox#4) lines. The transcript content was quantified relative to 10,000 molecules of actin 2/8 mRNA (n = 3; ±SD) and normalized to the amount in Col-0, which was set to 1.0 (for numerical values, see S1C Data). Please note that the y-axis for ox#4 (right) is scaled up 10-fold. (D) Immunoblot of At-FAX1 on total protein extracts isolated from leaf material of 30-day-old fax1–2, Col-0, Co#54, ox#2, ox#4 plants (see [C]). Please note that for detection of signals in all samples, different amounts of protein were loaded: 80, 80, 40, 80, and 5μg, respectively. Antiserum against the inner envelope protein TIC62 was used as loading control. For comparison, purified inner env [file pbio.1002053.s002.tif]

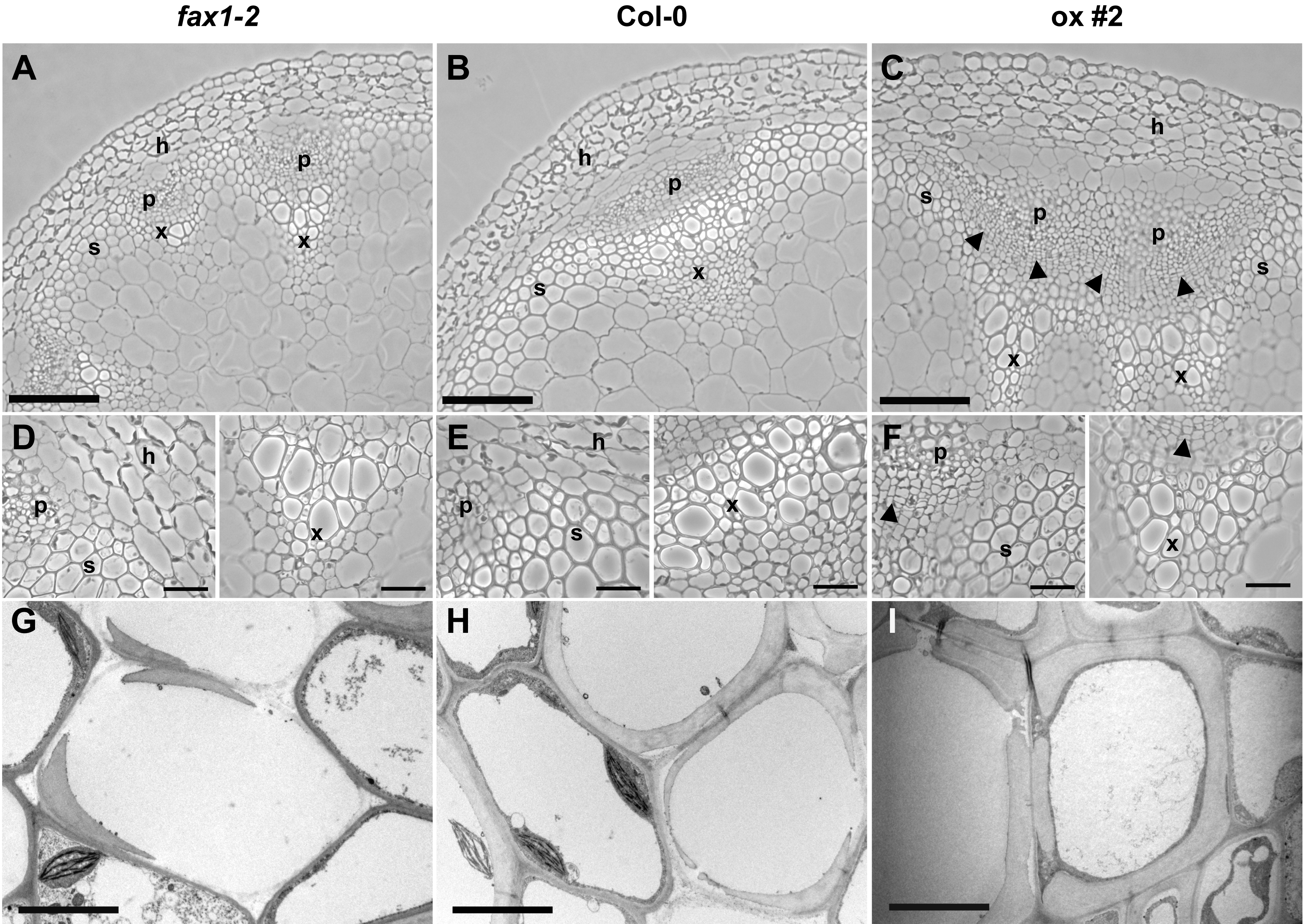

Supplement: S2 Fig — Cross-sections and vascular tissue of primary inflorescence stems (bottom part of second internode) from 5-week-old homozygous fax1–2 knockout [(A), (D), (G)], Col-0 wild-type [(B), (E), (H)] and the FAX1 over-expressor ox#2 [(C), (F), (I)]. (A), (B), (C) Overview of stem cross sections (light microscopy, bar = 100 μm). (D), (E), (F) Close-up of sclerenchyma/phloem (left) and xylem (right) (light microscopy, bar = 25 μm). (G), (H), (I) Cell walls of tracheids in xylem tissue (TEM, bar = 5 μm). h: hypodermis; p: phloem; s: sclerenchyma; x: xylem. Please note that FAX1ox#2 stems are characterized by an increased amount of xylem and phloem vessels as well as by a multi-layered procambium as depicted by arrowheads in (C) and (F). (TIF) [file pbio.1002053.s003.tif]

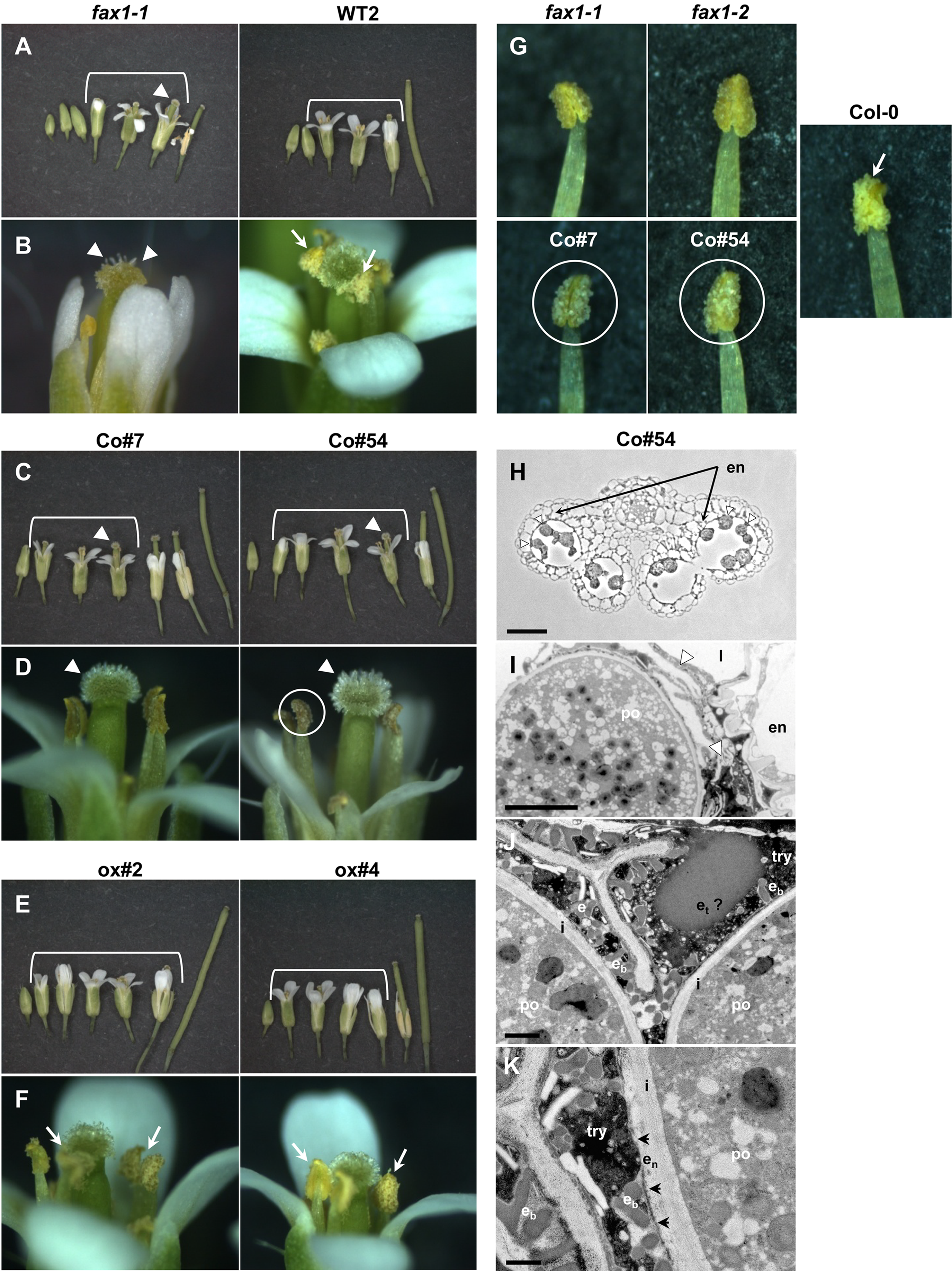

Supplement: S3 Fig — Pictures of flowers, anthers, and mature pollen of 5-week-old fax1–1 knockout, WT2 wild-type, complementation lines Co#7, Co#54, and FAX1 over-expressors ox#2, ox#4. (A), (C), (E) Development of flower buds and young siliques. Brackets indicate flower stages 10–15 [53] used for FA/lipid and microarray analysis. (B), (D), (F) Close-up of opened flowers. Arrowheads: non- or weakly pollinated stigma in fax1–1 (B) and Co#7, Co#54 (D), respectively; arrows: anthers with released pollen in WT2, ox#2, and ox#4; white circles: colorless pollen grains, released by Co#54 anther. (G) Close-up of dehiscent anthers. Please note that while fax1 k.o. anthers do not release pollen grains, Co#7 and Co#54 anthers produce few and colorless (white circles), and Col-0 wild-type generate many, yellow pollen, respectively. (H) Cross section of mature, dehisced anther of line Co#54 (light microscopy, bar = 50 μm). The appearance of Co#54 anthers is intermediate to that of fax1–2 and Col-0 (compare Fig. 4C). White arrowheads indicate that still some debris material is sticking to the pollen grain/endothecium boundary. en: endothecium cells of anthers. (I), (J), (K) TEM pictures of anther cell/pollen grain intersections in Co#54 (I, bar = 5 μm; J, bar = 1μm) and pollen cell wall (J, bar = 500 nm) at mature tricellular pollen stages. Please note that still some debris material is sticking to pollen grains (white arrowheads) and that in comparison to wild type (see Fig. 4E, F), the pollen exine is not fully established. For example, tectum structures seem to be absent, and the trypine pollen coat is not correctly assembled. en: endothecium cell; e: exine layer with eb: bacula structures; en: nexine layer (black arrowheads), i: intine layer; po: cytosol of pollen grain; try: tryphine pollen coat. (TIF) [file pbio.1002053.s004.tif]

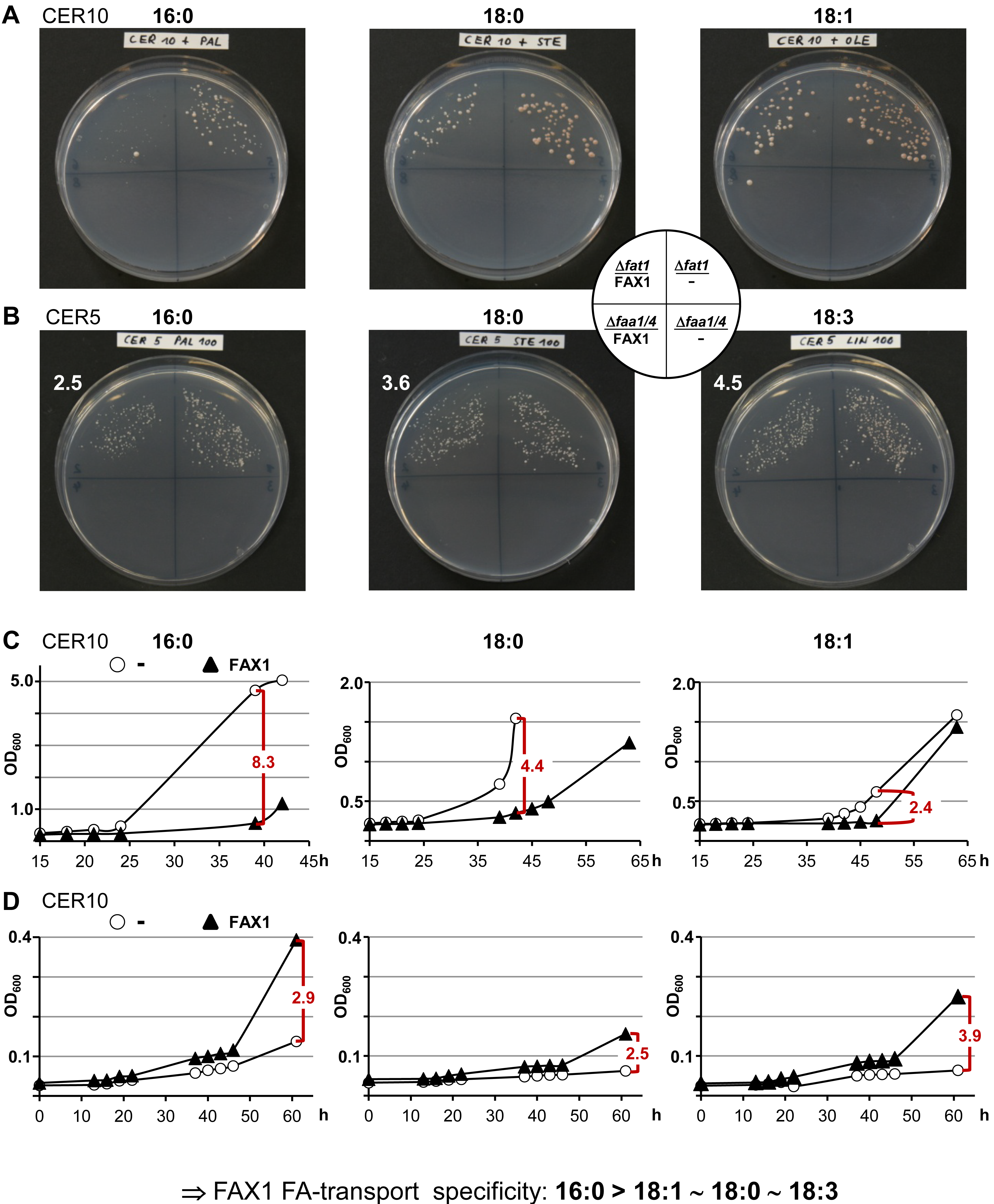

Supplement: S4 Fig — The mature At-FAX1 cDNA in pDR195 (FAX1) and the empty plasmid pDR195 (-) were introduced into fat1 and faa1/faa4 yeast mutants, respectively (compare Fig. 8). Growth assays were performed in the presence of 5–10 μM cerulenin (CER, inhibitor of FA-biosynthesis) and 100μM FAs according to [26,27]. To test for FAs, which in planta have to be exported from chloroplasts (see [1]), we used palmitic acid (PAL, C16:0), stearic acid (STE, C18:0), and oleic acid (OLE, C18:1). For results with the control α-linolenic acid (LIN, C18:3), which in vivo is not exported from plastids, see (B), right panel, and Fig. 8D. (A), (B) For growth on solid medium, 2 μl of exponentially growing yeast cells (diluted to an OD600 of 0.1/ml) were grown at 30°C on SD-ura plates (2% glucose, 0.5% Brij 58, 0.7% KH2PO4) in the presence of 10μM (A) and 5μM (B) CER, respectively. Cartoon: distribution of different strains on plates. Whereas faa1/faa4 mutants (on lower halves of plates) did not grow at all, fat1 cells transformed with the empty plasmid (-, upper right quarter) showed colonies in all assays after four to six days of incubation. In contrast, growth of fat1 with FAX1 (upper left quarter on plates with 10μM CER in [A]) was strongly restricted in the presence of PAL (left) and reduced with STE (middle) and OLE (right panel). In the presence of 5μM CER (B), growth inhibition by FAX1 was not as strong but still differential, resulting in an OD600/ml of all cells grown in the upper left quarter of 2.5 (for PAL), 3.6 (for STE), 4.0 (for OLE, see S1D Data), and 4.5 (for LIN), respectively. (C), (D) Growth of fat1 cells in liquid SD-ura with 10μM CER [see (A)]. White circles and black triangles: growth of pDR195 (-) and matFAX1/pDR195 (FAX1) cells. Red bars and numbers indicate maximal difference of cell density ratios, for numerical values see S1D Data. As on plates in (A) and (B), liquid cultures of faa1/faa4 cells did not grow (see S1D Data). (C) Cell growth at 30°C was started with an OD600 [file pbio.1002053.s005.tif]

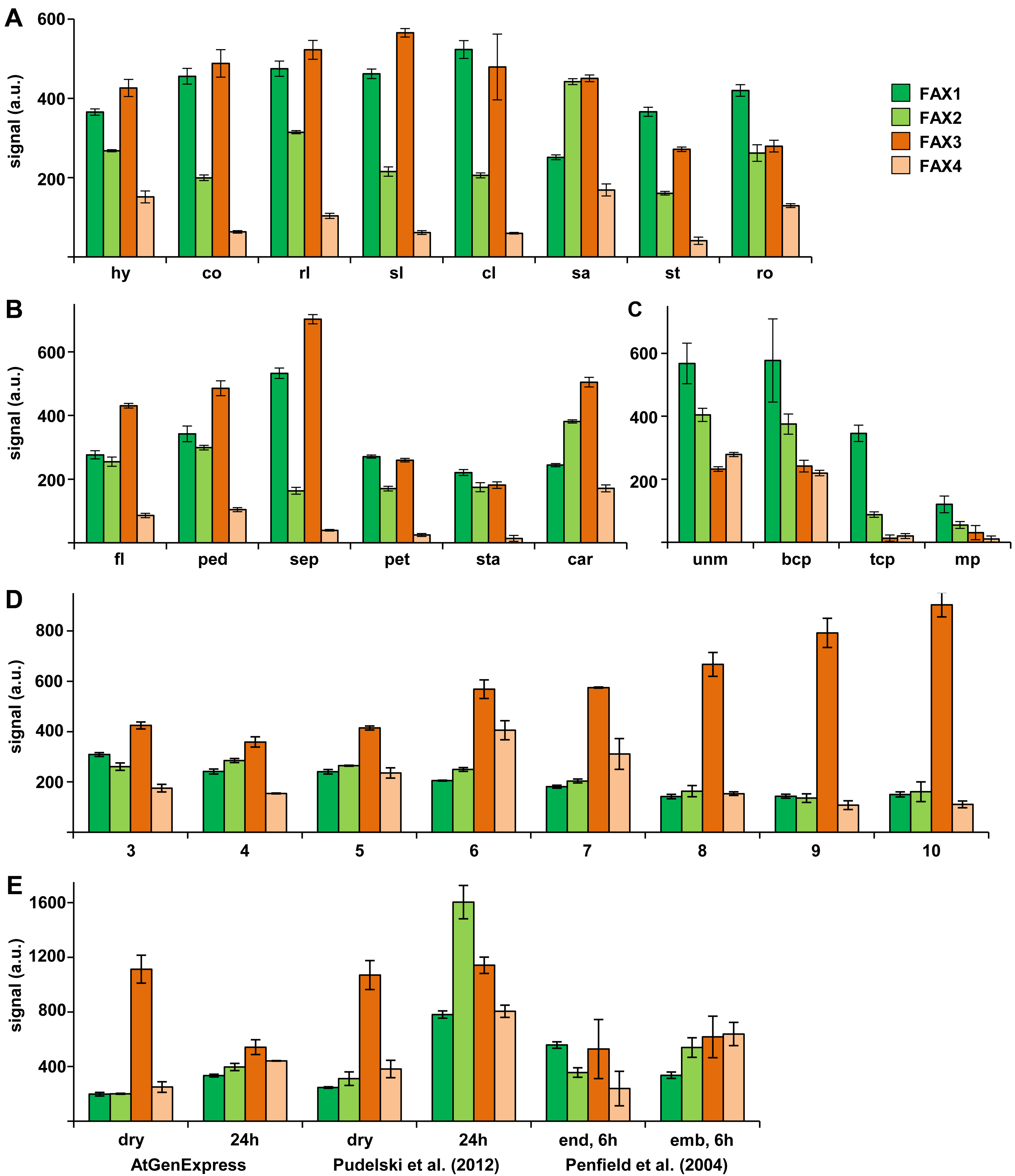

Supplement: S5 Fig — Expression profiles of Arabidopsis FAX1, FAX2, FAX3, FAX4 (green, light green, orange, light orange bars, respectively) during development. Data used to create digital Northern blots are based on DNA microarray analyses obtained from AtGenExpress developmental series (A–E; [61]), pollen development arrays (C; [62]), and from different seed microarray analyses (E; [63,64]). If not denoted elsewhere, the ecotype is Col-0. Mean signal intensities in (A)–(E) were averaged from two to three replicates (arbitrary units ± SD, for numerical values see S1E Data). Since expression data for FAX1–4 in seed tissue is generally high but differs between experiments and ecotypes (see also S6 Fig.), we show representative data in (D) and (E): (i) In general, FAX1 expression in seeds is low when compared to all other plastid FAX genes (see D, E, S6A Fig.). (ii) In contrast, FAX3 expression is quite strong in mature and dry seeds (see D, E). (iii) However, upon imbibition in aqueous solutions, expression of FAX2 and FAX4 is induced as well (see E, S6A Fig.), so that upon germination most likely FAX2, FAX3, and FAX4 are predominant. (iv) Whereas FAX2 and FAX3 are expressed in seed coat, endosperm and embryo of mature seeds, FAX1 and FAX4 transcripts are absent in seed coats (see S6A Fig.). (A), (B) Developmental series. Tissues and organs are specified as follows, age of plants in days grown in continuous light is indicated in brackets. (A) hy: hypocotyl (7); co: cotyledon (7); rl: rosette leaf no. 10 (17); sl: senescing leaf (35); cl: cauline leaf (21+); sa: shoot apex, before bolting (14); st: stem, second internode (21+); ro: root (17). (B) Dissected mature, open flowers (21+), stage 15 according to [53]. fl: total flower; ped: pedicel; sep: sepal; pet: petal; sta: stamen; car: carpel. (C) Pollen development. unm: uninucleate microspore; bcp: bicellular pollen; tcp: tricellular pollen; mp: mature pollen grain (mp data points from AtGenExpress). (D) Embryo and seed development. Seed [file pbio.1002053.s006.tif]

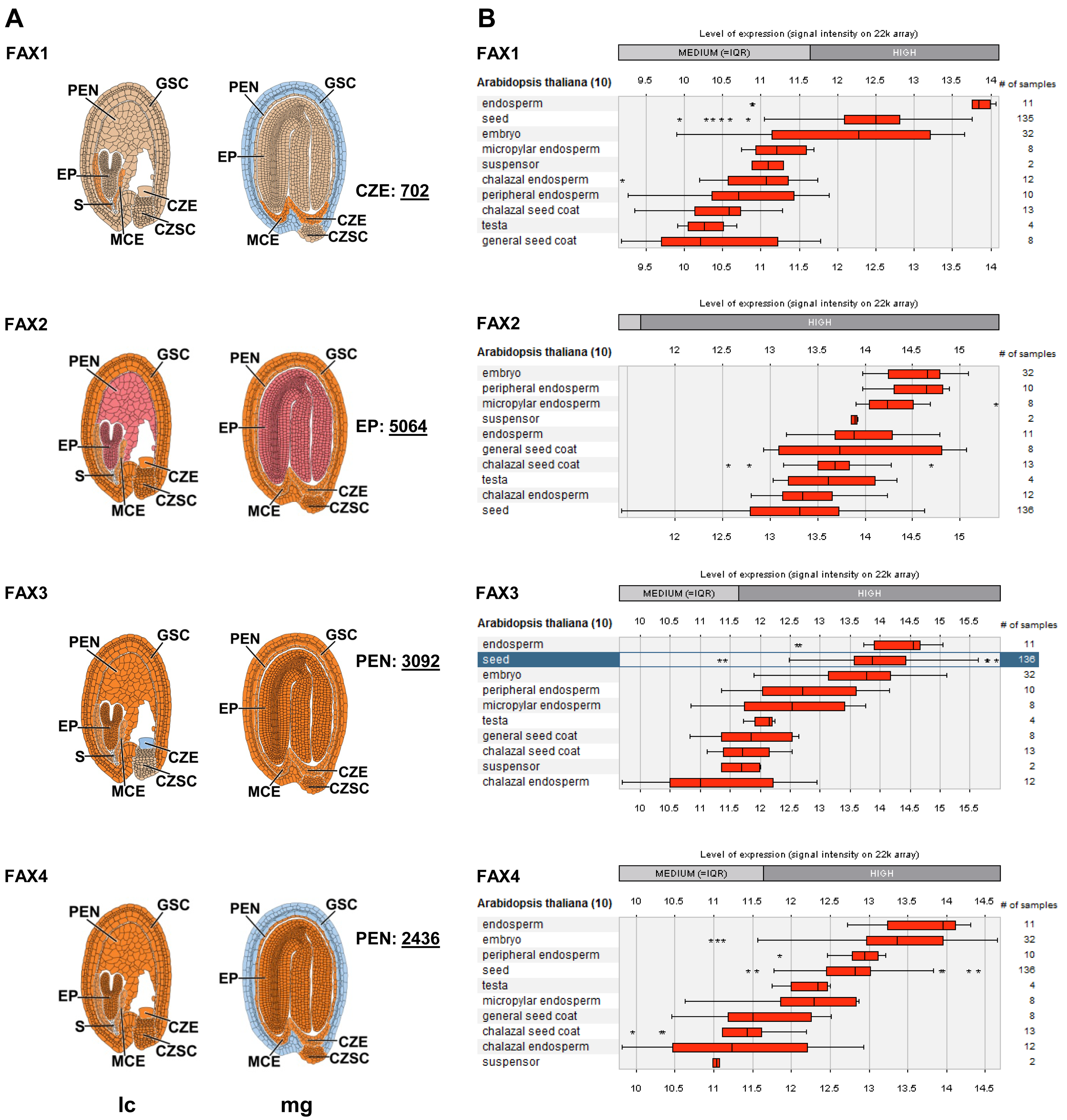

Supplement: S6 Fig — (A) Expression of FAX1–4 in tissues of linear cotyledon (lc) and maturation green (mg) stage embryos in late seed development (Harada-Goldberg dataset of laser capture microdissected seeds: “Gene Networks in Seed Development”). Please note that during fixation, tissue was submerged in aqueous solutions and therefore transcript levels of FAX2 and FAX4 might resemble those of imbibed tissue in S5E Fig. CZE, chalazal endosperm; CZSC, chalazal seed coat; EP, embryo proper; GSC, general seed coat; MCE, micropylar endosperm; PEN, peripheral endosperm; S, suspensor. Tissues are colored according to transcript density for signals that are absent (white), insufficient (blue), <500 (beige), 500–5,000 (orange), 5,000–10,000 (purple), and >10,000 (dark red). Highest expression in mature seed tissue is indicated. Data is available at http://estdb.biology.ucla.edu/seed/. (B) Seed anatomy series from the genevestigator database [65]. Please note that expression from large sets of samples including different ecotypes and experimental setups is depicted as boxplots of log2 values. Data is available at https://www.genevestigator.com/gv/. (TIF) [file pbio.1002053.s007.tif]

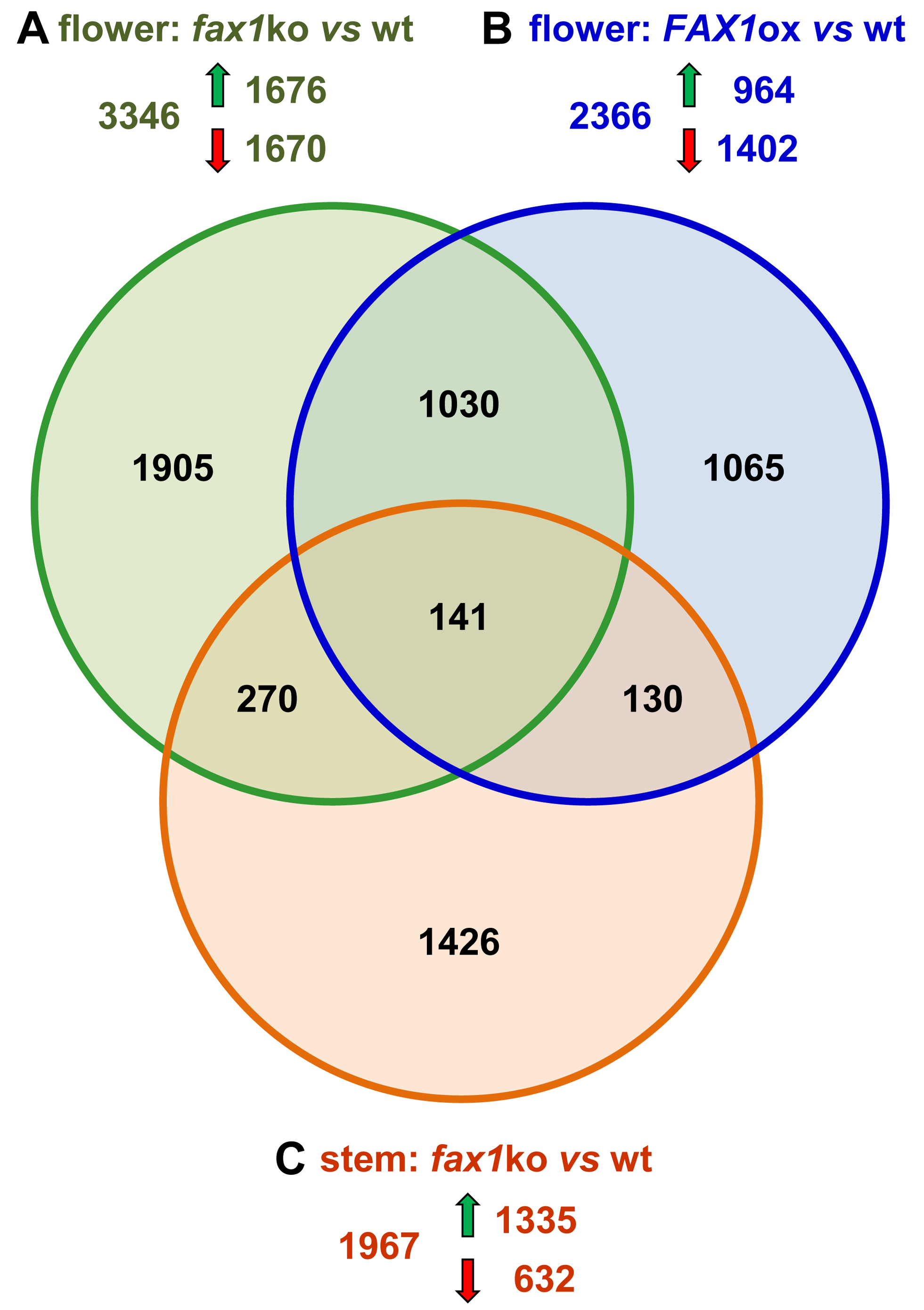

Supplement: S7 Fig — Venn diagram summarizing numbers and overlaps of significantly regulated genes (p-value ≤ 0.05) from DNA microarray analysis (ATH1 GeneChip) of FAX1 mutants in flower and stem tissues (see E-MTAB-3090 at www.ebi.ac.uk/arrayexpress). (A) Comparison fax1 knockout (n = 5) versus wild type (n = 5) in flower tissue. Of 3346 differentially regulated genes, 1676 were significantly up-regulated, whereas 1670 were down-regulated in fax1 knockout flowers. (B) Comparison FAX1 over-expressors (n = 8, 4 times each line ox#2, ox#4) versus wild type (n = 5) in flower tissue. In flowers of FAX1 over-expressors 2366 genes showed to be significantly regulated (964 up, 1402 down). (C) Comparison fax1 knockout (n = 4) versus wild type (n = 4) in stem tissue. Of 1967 differentially regulated genes, 1335 were significantly up-regulated, whereas 632 were down-regulated in fax1 knockout stems. (TIF) [file pbio.1002053.s008.tif]

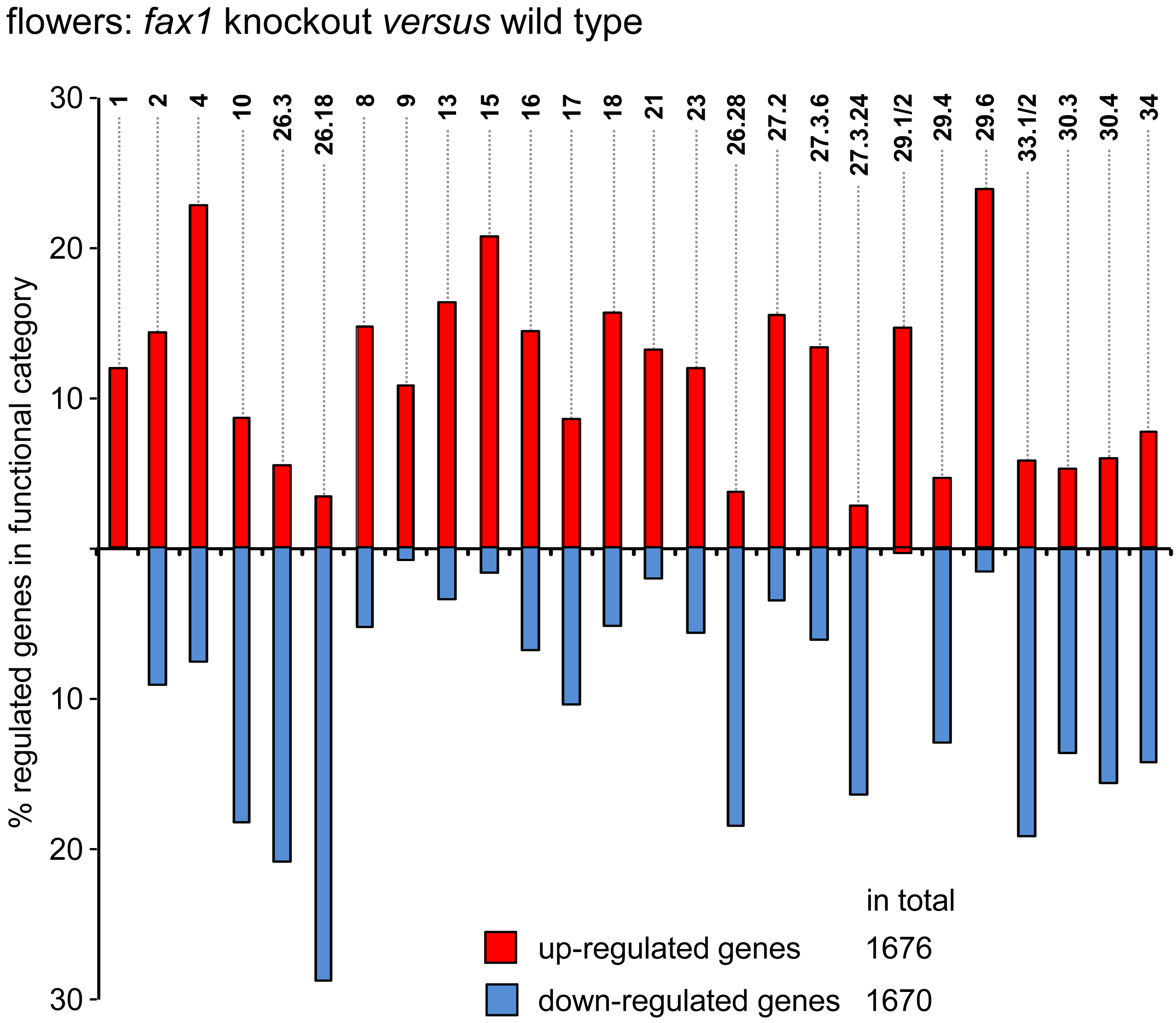

Supplement: S8 Fig — Results of DNA microarray analysis (ATH1 GeneChip) for the comparisons depicted in S7A Fig.: fax1 knockout (n = 5) versus wild type (n = 5) in flower tissue. For better visualization, we sub-divided TAIR10 functional categories (Ath_AFFY_ATH1_TAIR10_Aug2012; http://mapman.gabipd.org) into portions containing between 50–600 genes. Furthermore, we displayed only those categories containing more than 10% of significantly regulated genes (p-value ≤ 0.05), respectively (see S1F Data for numerical values). The complete microarray data are available in the ArrayExpress database (www.ebi.ac.uk/arrayexpress) under accession number E-MTAB-3090. Depicted functional categories are as follows: 1. photosynthesis; 2. major CHO metabolism; 4. glycolysis; 10. cell wall; 26.3. gluco-, galacto- and mannosidases; 26.18. invertase + pectin methylesterase inhibitor family; 8. TCA cycle / org transformation; 9. mitochondrial electron transport; 13. amino acid metabolism; 15. metal handling; 16. secondary metabolism; 17. hormone metabolism; 18. Co-factor and vitamin metabolism; 21. redox; 23. nucleotide metabolism; 26.28. GDSL-motif lipases; 27.2. transcription; 27.3.6. regulation of transcription: bHLH, Basic Helix-Loop-Helix family; 27.3.24. regulation of transcription: MADS box transcription factor family; 29.1./2. protein aa activation/protein synthesis; 29.4. protein postranslational modification; 29.6. protein folding; 33.1./2. development: storage/late embryogenesis abundant proteins; 30.3. signalling calcium; 30.4. signalling phosphoinositides; 34. transport. (TIF) [file pbio.1002053.s009.tif]

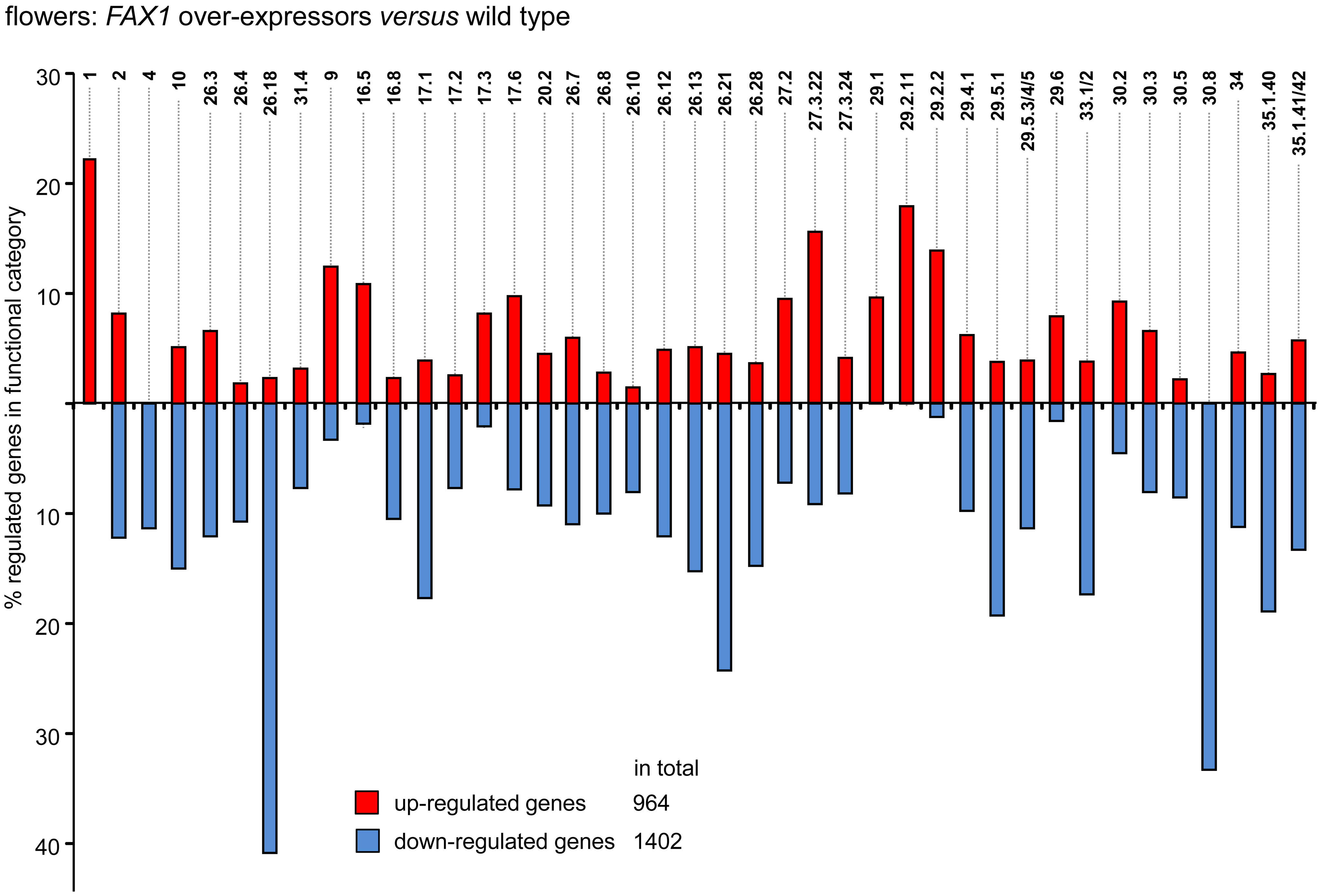

Supplement: S9 Fig — Results of DNA microarray analysis (ATH1 GeneChip) for the comparisons depicted in S7B Fig.: FAX1 over-expressors (n = 8, 4 times each line ox#2, ox#4) versus wild type (n = 5) in flower tissue. For better visualization, we sub-divided TAIR10 functional categories (Ath_AFFY_ATH1_TAIR10_Aug2012; http://mapman.gabipd.org) into portions containing between 50–600 genes. Furthermore, we displayed only those categories containing more than 7.5% of significantly regulated genes (p-value ≤ 0.05), respectively (see S1F Data for numerical values). The complete microarray data are available in the ArrayExpress database (www.ebi.ac.uk/arrayexpress) under accession number E-MTAB-3090. Depicted functional categories are as follows: 1. photosynthesis; 2. major CHO metabolism; 4. glycolysis: cytosolic branch; 10. cell wall; 26.3 gluco-, galacto- and mannosidases; 26.4 beta 1,3 glucan hydrolases; 26.18 invertase + pectin methylesterase inhibitor family protein; 31.4 cell: vesicle transport; 9. mitochondrial electron transport; 16.5 secondary metabolism: sulfur-containing.glucosinolates; 16.8 secondary metabolism: flavonoids; 17.1 hormone metabolism: abscisic acid; 17.2 hormone metabolism: auxin; 17.3 hormone metabolism: brassinosteroid; 17.6 hormone metabolism: gibberelin; 20.2 stress abiotic; 26.7 oxidases—copper, flavone etc.; 26.8 nitrilases, nitrile lyases, berberine bridge enzymes, reticuline oxidases, troponine reductases; 26.10 cytochrome P450; 26.12 peroxidases; 26.13 acid and other phosphatases; 26.21 protease inhibitor/seed storage/lipid transfer protein (LTP) family protein; 26.28 GDSL-motif lipase; 27.2 transcription; 27.3.22 regulation of transcription: HB, Homeobox transcription factor family; 27.3.24 regulation of transcription: MADS box transcription factor family; 29.1 protein aa activation; 29.2.11 protein synthesis: ribosomal protein.prokaryotic; 29.2.2 protein synthesis: ribosome biogenesis; 29.4.1 protein: postranslational modification.kinase; 29.5.1 protein: de [file pbio.1002053.s010.tif]

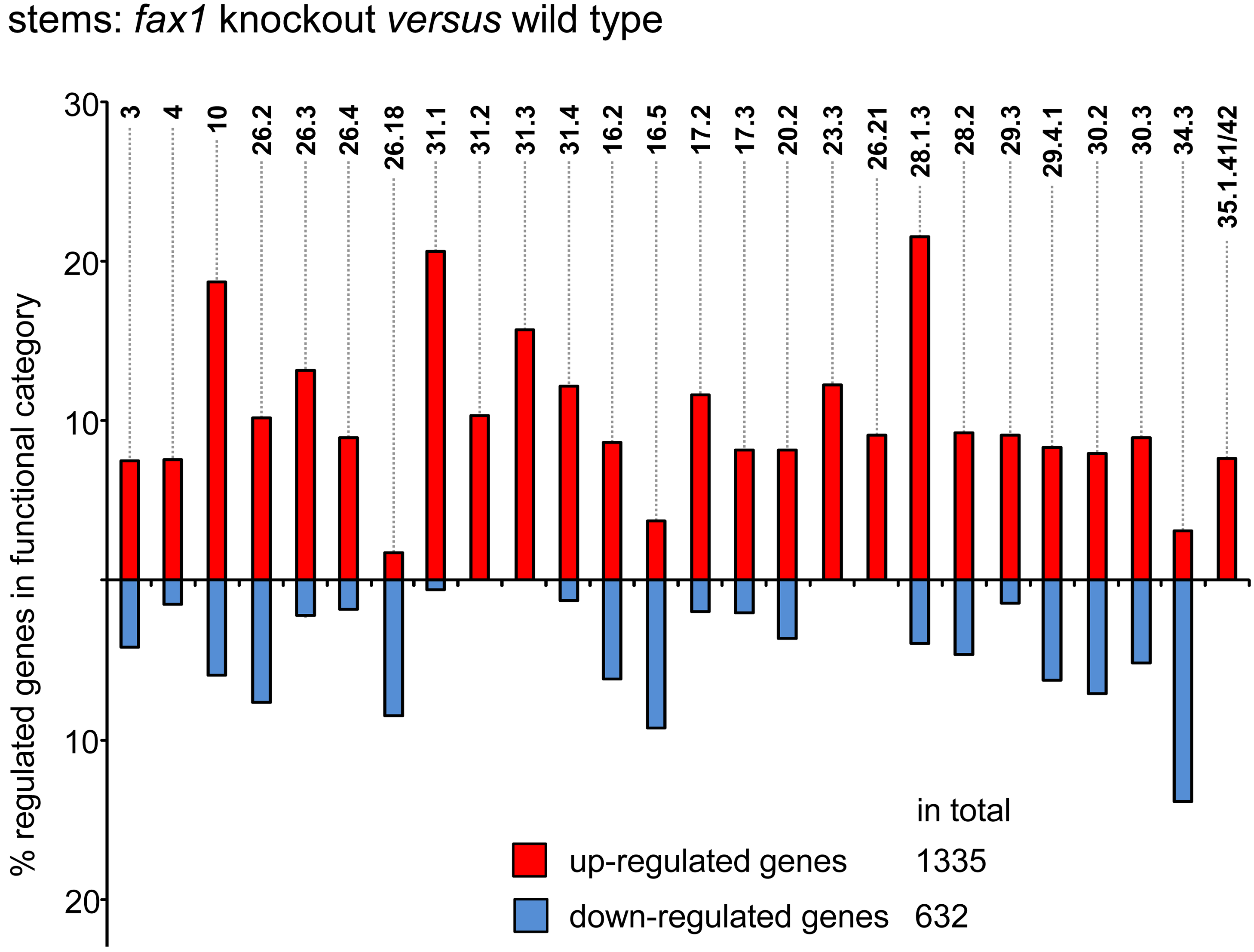

Supplement: S10 Fig — Results of DNA microarray analysis (ATH1 GeneChip) for the comparisons depicted in S7C Fig.: fax1 knockout (n = 4) versus wild type (n = 4) in stem tissue. For better visualization, we sub-divided TAIR10 functional categories (Ath_AFFY_ATH1_TAIR10_Aug2012; http://mapman.gabipd.org) into portions containing between 50–600 genes. Furthermore, we displayed only those categories containing more than 7.5% of significantly regulated genes (p-value ≤ 0.05), respectively (see S1F Data for numerical values). The complete microarray data are available in the ArrayExpress database (www.ebi.ac.uk/arrayexpress) under accession number E-MTAB-3090. Depicted functional categories are as follows: 3. minor CHO metabolism; 4. glycolysis; 10. cell wall; 26.2 UDP glucosyl and glucoronyl transferases; 26.3 gluco-, galacto- and mannosidases; 26.4 beta 1,3 glucan hydrolases; 26.18 invertase + pectin methylesterase inhibitor family; 31.1 cell organisation; 31.2 cell division; 31.3 cell cycle; 31.4 cell: vesicle transport; 16.2 secondary metabolism: phenypropanoids; 16.5 secondary metabolism: sulfur-containing glucosinolates; 17.2 hormone metabolism: auxin; 17.3 hormone metabolism: brassinosteroid; 20.2 stress abiotic; 23.3 nucleotide metabolism: salvage; 26.21 protease inhibitor/seed storage/lipid transfer protein (LTP) family protein; 28.1.3 DNA synthesis: chromatin structure.histone; 28.2 DNA repair; 29.3 protein targeting; 29.4.1 protein: postranslational modification.kinase; 30.2 receptor kinases; 30.3 signalling calcium; 34.3 transport: amino acids/ammonium; 35.1.41/42 hydroxyproline + proline rich protein family. (TIF) [file pbio.1002053.s011.tif]
