# Supplementary material for: FAX1, a Novel Membrane Protein Mediating Plastid Fatty Acid Export
Source: PLoS Biol. 2015 Feb 3;13(2):e1002053. doi: 10.1371/journal.pbio.1002053 (PMC4344464; doi:10.1371/journal.pbio.1002053)
Supplement: S2 Table — Content (mol %) of free FAs and polar lipids, determined in caulinary leaves of 7-week-old, mature plants. Please note that only species significantly different in FAX1 mutants (mu) compared to wild type (wt) are depicted. For a complete dataset, details on samples, and significance analysis see S1 Table. Samples and subdivision into different species (A–D) are identical to Fig. 6. Numbers in subheadings indicate significantly different species versus all molecules measured (see S1 Table). The direction of changes (↑: up; ↓: down), the fold change (FCH), and the differences of mol% in FAX1 mutants versus wild type are given. Asterisks label the two most abundant species of each molecule class determined (compare S1 Table). DGDG: digalactosyl-diacylglycerol; FA: free fatty acid; MGDG: monogalactosyl-diacylglycerol; PC: phosphatidyl-choline; PE: phosphatidyl-ethanolamine; PG: phosphatidyl-glycerol; PI: phosphatidyl-inositol; SQDG: sulphoquinovosyl-diacylglycerol. (DOCX) [file pbio.1002053.s013.docx]

**Table S2. Plastid FAX1 impacts cellular FA/lipid homeostasis in leaves.**

|  | **mu [mol%]** | **wt**  **[mol%]** | **change**  **mu *vs* wt** | **FCH** | **diff**  **mu *vs* wt** |
| --- | --- | --- | --- | --- | --- |

**(A) *fax1* ko: FA species from plastids, 34:x glycolipids, PG**: 10/23

| FA 18:2 | 0.163 | 0.070 | up | **2.31 ↑** | **0.092** |
| --- | --- | --- | --- | --- | --- |
| MGDG 34:1 | 0.062 | 0.029 | up | **2.17 ↑** | **0.034** |
| MGDG 34:2 | 0.224 | 0.142 | up | **1.58 ↑** | **0.082** |
| MGDG 34:3 | 0.423 | 0.451 | down | **1.07 ↓** | **-0.028** |
| DGDG 34:2 | 0.810 | 0.445 | up | **1.82 ↑** | **0.365** |
| SQDG 34:1 | 0.032 | 0.017 | up | **1.88 ↑** | **0.015** |
| PG 34:1 | 0.382 | 0.076 | up | **5.01 ↑** | **0.306** |
| PG 34:2 | 0.886 | 0.283 | up | **3.13 ↑** | **0.603** |
| PG 34:3 ***** | 1.945 | 0.647 | up | **3.01 ↑** | **1.298** |
| PG 34:4 ***** | 1.505 | 0.547 | up | **2.75 ↑** | **0.958** |

**(B) FAX1ox: FA species from plastids, 34:x glycolipids, PG**: 17/21

| FA 16:0 | 0.059 | 0.066 | down | **1.13 ↓** | **-0.008** |
| --- | --- | --- | --- | --- | --- |
| FA 18:0 | 0.041 | 0.049 | down | **1.19 ↓** | **-0.008** |
| FA 18:1 | 0.031 | 0.039 | down | **1.25 ↓** | **-0.008** |
| FA 18:2 | 0.011 | 0.013 | down | **1.23 ↓** | **-0.002** |
| MGDG 34:1 | 0.114 | 0.293 | down | **2.57 ↓** | **-0.179** |
| MGDG 34:2 | 0.335 | 0.499 | down | **1.49 ↓** | **-0.164** |
| MGDG 34:4 | 0.143 | 0.340 | down | **2.38 ↓** | **-0.197** |
| MGDG 34:5 | 1.480 | 2.226 | down | **1.50 ↓** | **-0.746** |
| MGDG 34:6 ***** | 9.210 | 10.676 | down | **1.16 ↓** | **-1.466** |
| DGDG 34:2 | 1.371 | 2.235 | down | **1.63 ↓** | **-0.864** |
| DGDG 34:3 ***** | 5.957 | 6.543 | down | **1.10 ↓** | **-0.586** |
| DGDG 34:5 | 0.462 | 0.532 | down | **1.15 ↓** | **-0.070** |
| DGDG 34:6 | 2.525 | 2.852 | down | **1.13 ↓** | **-0.327** |
| SQDG 34:1 | 0.025 | 0.038 | down | **1.53 ↓** | **-0.013** |
| SQDG 34:2 | 0.194 | 0.379 | down | **1.95 ↓** | **-0.184** |
| SQDG 34:3 ***** | 2.132 | 2.877 | down | **1.35 ↓** | **-0.745** |
| PG 34:2 | 0.125 | 0.142 | down | **1.13 ↓** | **-0.017** |

**(C) *fax1* ko: FA/lipid species derived from ER/cytosol**: 20/33

| FA 20:0 | 0.045 | 0.101 | down | **2.25 ↓** | **-0.056** |
| --- | --- | --- | --- | --- | --- |
| FA 24:0 | 0.021 | 0.029 | down | **1.41 ↓** | **-0.009** |
| FA 26:0 | 0.110 | 0.147 | down | **1.34 ↓** | **-0.038** |
| MGDG 36:6 ***** | 9.018 | 11.731 | down | **1.30 ↓** | **-2.714** |
| DGDG 36:6 ***** | 9.633 | 8.910 | up | **1.08 ↑** | **0.724** |
| SQDG 36:6 ***** | 1.656 | 1.619 | up | **1.02 ↑** | **0.036** |
| PC 34:3 ***** | 5.978 | 9.290 | down | **1.55 ↓** | **-3.312** |
| PC 34:4 | 0.100 | 0.134 | down | **1.34 ↓** | **-0.034** |
| PC 34:5 | 0.055 | 0.075 | down | **1.36 ↓** | **-0.020** |
| PC 34:6 | 0.071 | 0.146 | down | **2.06 ↓** | **-0.075** |
| PC 36:2 | 0.167 | 0.145 | up | **1.16 ↑** | **0.023** |
| PC 36:3 | 0.254 | 0.702 | down | **2.77 ↓** | **-0.448** |
| PC 36:5 | 4.281 | 5.010 | down | **1.17 ↓** | **-0.728** |
| PC 36:6 ***** | 3.188 | 7.396 | down | **2.32 ↓** | **-4.207** |
| PE 34:2 | 0.304 | 0.190 | up | **1.59 ↑** | **0.113** |
| PE 34:3 ***** | 0.665 | 0.849 | down | **1.28 ↓** | **-0.184** |
| PE 36:5 ***** | 0.725 | 0.761 | down | **1.05 ↓** | **-0.037** |
| PE 36:6 | 0.294 | 0.652 | down | **2.21 ↓** | **-0.357** |
| PI 34:2 | 0.033 | 0.012 | up | **2.83 ↑** | **0.022** |
| PI 34:3 | 0.069 | 0.014 | up | **5.04 ↑** | **0.055** |

**(D) FAX1ox: FA/lipid species derived from ER/cytosol:** 11/29

| MGDG 36:3 | 0.046 | 0.031 | up | **1.50 ↑** | **0.015** |
| --- | --- | --- | --- | --- | --- |
| MGDG 36:5 ***** | 1.843 | 2.232 | down | **1.21 ↓** | **-0.389** |
| SQDG 36:4 | 0.043 | 0.051 | down | **1.20 ↓** | **-0.009** |
| SQDG 36:5 | 0.146 | 0.174 | down | **1.19 ↓** | **-0.028** |
| PC 34:1 | 1.497 | 0.892 | up | **1.68 ↑** | **0.604** |
| PC 34:3 ***** | 8.809 | 7.253 | up | **1.21 ↑** | **1.556** |
| PC 34:4 | 0.661 | 0.761 | down. | **1.15 ↓** | **-0.101** |
| PC 34:5 | 0.112 | 0.152 | down | **1.35 ↓** | **-0.040** |
| PC 36:2 | 1.049 | 0.619 | up | **1.69 ↑** | **0.429** |
| PC 36:3 | 1.680 | 1.170 | up | **1.44 ↑** | **0.510** |
| PE 34:3 ***** | 0.533 | 0.452 | up | **1.18 ↑** | **0.081** |
