# Supplementary material for: FAX1, a Novel Membrane Protein Mediating Plastid Fatty Acid Export
Source: PLoS Biol. 2015 Feb 3;13(2):e1002053. doi: 10.1371/journal.pbio.1002053 (PMC4344464; doi:10.1371/journal.pbio.1002053)
Supplement: S3 Table — Content (mol %) of free FAs and polar lipids, determined in flower tissue of 7-week-old, mature plants. Please note that only species significantly different in FAX1 mutants (mu) compared to wild type (wt) are depicted. For a complete dataset, details on samples and significance analysis see S1 Table. Subdivision into different species (A–D) is similar to Fig. 6; numbers in subheadings indicate significantly different species versus all molecules measured (see S1 Table). The direction of changes (↑: up; ↓: down), the fold change (FCH), and the differences of mol% in FAX1 mutants versus wildtype are given. Asterisks label the two most abundant species of each molecule class determined (compare S1 Table). DGDG: digalactosyl-diacylglycerol; FA: free fatty acid; MGDG: monogalactosyl-diacylglycerol; PC: phosphatidyl-choline; PE: phosphatidyl-ethanolamine; PG: phosphatidyl-glycerol; PI: phosphatidyl-inositol; SQDG: sulphoquinovosyl-diacylglycerol. (DOCX) [file pbio.1002053.s014.docx]

**Table S3. Plastid FAX1 impacts cellular FA/lipid homeostasis in flowers.**

|  | **mu [mol%]** | **wt**  **[mol%]** | **change**  **mu *vs* wt** | **FCH** | **diff**  **mu *vs* wt** |
| --- | --- | --- | --- | --- | --- |

**(A) *fax1* ko: FA species from plastids, 34:x glycolipids, PG**: 9/22

| FA 16:0 | 0.630 | 0.391 | up | **1.61 ↑** | **0.240** |
| --- | --- | --- | --- | --- | --- |
| FA 18:0 | 0.517 | 0.475 | up | **1.09 ↑** | **0.042** |
| FA 18:1 | 0.252 | 0.240 | up | **1.05 ↑** | **0.013** |
| FA 18:3 | 1.324 | 1.460 | down | **1.10 ↓** | **-0.136** |
| MGDG 34:1 | 0.014 | 0.006 | up | **2.11 ↑** | **0.007** |
| MGDG 34:4 | 0.442 | 0.426 | up | **1.04 ↑** | **0.017** |
| MGDG 34:5 | 0.439 | 0.378 | up | **1.16 ↑** | **0.061** |
| MGDG 34:6 ***** | 8.144 | 7.505 | up | **1.09 ↑** | **0.640** |
| DGDG 34:6 ***** | 1.087 | 1.041 | up | **1.04 ↑** | **0.046** |

**(B) FAX1ox: FA species from plastids, 34:x glycolipids, PG**: 9/21

| FA 18:0 | 0.054 | 0.042 | up | **1.26 ↑** | **0.011** |
| --- | --- | --- | --- | --- | --- |
| MGDG 34:1 | 0.089 | 0.168 | down | **1.88 ↓** | **-0.079** |
| MGDG 34:2 | 0.254 | 0.374 | down | **1.47 ↓** | **-0.120** |
| MGDG 34:5 ***** | 1.103 | 1.406 | down | **1.27 ↓** | **-0.303** |
| DGDG 34:2 | 0.632 | 0.884 | down | **1.40 ↓** | **-0.252** |
| DGDG 34:5 | 0.164 | 0.197 | down | **1.20 ↓** | **-0.033** |
| SQDG 34:1 | 0.003 | 0.010 | down | **3.25 ↓** | **-0.007** |
| SQDG 34:2 | 0.073 | 0.121 | down | **1.65 ↓** | **-0.047** |
| SQDG 34:3 ***** | 0.595 | 0.745 | down | **1.25 ↓** | **-0.150** |

**(C) *fax1* ko: FA/lipid species derived from ER/cytosol**: 19/33

| FA 24:0 | 0.273 | 0.376 | down | **1.38 ↓** | **-0.103** |
| --- | --- | --- | --- | --- | --- |
| MGDG 36:4 | 0.162 | 0.141 | up | **1.15 ↑** | **0.021** |
| MGDG 36:5 | 0.510 | 0.452 | up | **1.13 ↑** | **0.059** |
| MGDG 36:6 ***** | 6.788 | 5.989 | up | **1.13 ↑** | **0.799** |
| DGDG 36:6 ***** | 5.859 | 4.882 | up | **1.20 ↑** | **0.977** |
| SQDG 36:4 | 0.035 | 0.031 | up | **1.15 ↑** | **0.005** |
| SQDG 36:5 | 0.118 | 0.123 | down | **1.04 ↓** | **-0.005** |
| SQDG 36:6 ***** | 0.500 | 0.489 | up | **1.02 ↑** | **0.012** |
| PC 34:5 | 0.007 | 0.009 | down | **1.35 ↓** | **-0.002** |
| PC 34:6 | 0.021 | 0.038 | down | **1.76 ↓** | **-0.016** |
| PC 36:2 | 0.185 | 0.212 | down | **1.15 ↓** | **-0.027** |
| PC 36:6 ***** | 2.692 | 3.737 | down | **1.39 ↓** | **-1.045** |
| PE 34:2 | 1.087 | 0.975 | up | **1.11 ↑** | **0.112** |
| PE 34:3 ***** | 1.474 | 1.270 | up | **1.16 ↑** | **0.204** |
| PE 36:4 | 0.806 | 0.742 | up | **1.09 ↑** | **0.064** |
| PE 36:5 | 0.965 | 0.857 | up | **1.13 ↑** | **0.108** |
| PE 36:6 | 0.600 | 0.668 | down | **1.11 ↓** | **-0.068** |
| PI 34:2 | 0.532 | 0.675 | down | **1.27 ↓** | **-0.143** |
| PI 34:3 | 0.870 | 0.991 | down | **1.14 ↓** | **-0.121** |

**(D) FAX1ox: FA/lipid species derived from ER/cytosol**: 18/31

| MGDG 36:3 | 0.027 | 0.023 | up | **1.20 ↑** | **0.005** |
| --- | --- | --- | --- | --- | --- |
| DGDG 36:5 | 0.452 | 0.595 | down | **1.32 ↓** | **-0.144** |
| SQDG 36:4 | 0.014 | 0.024 | down | **1.72 ↓** | **-0.010** |
| SQDG 36:5 | 0.045 | 0.057 | down | **1.27 ↓** | **-0.012** |
| PC 34:1 | 0.609 | 0.753 | down | **1.24 ↓** | **-0.144** |
| PC 34:3 ***** | 5.806 | 7.027 | down | **1.21 ↓** | **-1.221** |
| PC 34:4 | 0.936 | 1.038 | up | **1.11 ↓** | **-0.102** |
| PC 34:5 | 0.050 | 0.070 | down | **1.42 ↓** | **-0.021** |
| PC 34:6 | 0.021 | 0.042 | down | **2.01 ↓** | **-0.021** |
| PC 36:2 | 0.900 | 1.167 | down | **1.30 ↓** | **-0.267** |
| PC 36:3 | 0.915 | 1.145 | down | **1.25 ↓** | **-0.230** |
| PC 36:4 | 4.266 | 5.271 | down | **1.24 ↓** | **-1.005** |
| PC 36:5 | 4.362 | 5.526 | down | **1.27 ↓** | **-1.163** |
| PC 36:6 | 3.616 | 4.997 | down | **1.38 ↓** | **-1.381** |
| PE 34:3 ***** | 0.912 | 0.999 | up | **1.10 ↓** | **-0.087** |
| PE 36:4 | 0.754 | 0.834 | up | **1.11 ↓** | **-0.080** |
| PE 36:5 | 0.785 | 0.967 | down | **1.23 ↓** | **-0.182** |
| PE 36:6 | 0.213 | 0.337 | down | **1.58 ↓** | **-0.124** |
