# Supplementary material for: FAX1, a Novel Membrane Protein Mediating Plastid Fatty Acid Export
Source: PLoS Biol. 2015 Feb 3;13(2):e1002053. doi: 10.1371/journal.pbio.1002053 (PMC4344464; doi:10.1371/journal.pbio.1002053)
Supplement: S4 Table — Content (mol %) of triacylglycerol (TAG) oils, determined in tissues of 7-week-old, mature flowering plants. Please note that only species significantly different in FAX1 mutants (mu) compared to wild type (wt) are depicted. For a complete dataset, details on samples, and significance analysis see S1 Table. Samples and subdivision into (A–D) are identical to Fig. 7. Numbers in subheadings indicate significantly different species versus all molecules determined (see S1 Table). The direction of changes (↑: up; ↓: down), the fold change (FCH), and the differences of mol% in FAX1 mutants versus wild type are given. Asterisks label the five most abundant species of all TAGs measured (compare S1 Table). (DOCX) [file pbio.1002053.s015.docx]

**Table S4. Plastid FAX1 impacts TAG storage lipid homeostasis.**

|  | **mu [mol%]** | **wt**  **[mol%]** | **change**  **mu *vs* wt** | **FCH** | **diff**  **mu *vs* wt** |
| --- | --- | --- | --- | --- | --- |

**(A) *fax1* ko: TAG content in leaves**: 20/40

| TAG 48:0 | 0.046 | 0.055 | down | **1.18 ↓** | **-0.009** |
| --- | --- | --- | --- | --- | --- |
| TAG 48:1 | 0.064 | 0.107 | down | **1.67 ↓** | **-0.043** |
| TAG 48:2 | 0.034 | 0.059 | down | **1.74 ↓** | **-0.025** |
| TAG 50:1 | 0.090 | 0.108 | down | **1.21 ↓** | **-0.018** |
| TAG 50:2 | 0.165 | 0.186 | down | **1.13 ↓** | **-0.021** |
| TAG 50:3 | 0.085 | 0.173 | down | **2.03 ↓** | **-0.087** |
| TAG 50:5 | 0.019 | 0.022 | down | **1.17 ↓** | **-0.003** |
| TAG 52:1 | 0.045 | 0.064 | down | **1.42 ↓** | **-0.019** |
| TAG 52:2 | 0.203 | 0.237 | down | **1.16 ↓** | **-0.033** |
| TAG 52:3 | 0.243 | 0.292 | down | **1.20 ↓** | **-0.049** |
| TAG 52:4 | 0.203 | 0.314 | down | **1.55 ↓** | **-0.112** |
| TAG 52:5 ***** | 0.933 | 1.346 | down | **1.44 ↓** | **-0.413** |
| TAG 52:6 | 0.773 | 0.837 | down | **1.08 ↓** | **-0.064** |
| TAG 52:7 | 0.101 | 0.110 | down | **1.09 ↓** | **-0.009** |
| TAG 54:1 | 0.029 | 0.054 | down | **1.88 ↓** | **-0.025** |
| TAG 54:5 | 0.784 | 0.729 | up | **1.07 ↑** | **0.055** |
| TAG 54:6 | 0.241 | 0.275 | down | **1.14 ↓** | **-0.034** |
| TAG 54:7 ***** | 1.003 | 0.899 | up | **1.12 ↑** | **0.104** |
| TAG 54:8 ***** | 1.838 | 2.295 | down | **1.25 ↓** | **-0.457** |
| TAG 54:9 ***** | 1.306 | 2.161 | down | **1.66 ↓** | **-0.856** |
| TAG 56:2 | 0.062 | 0.087 | down | **1.41 ↓** | **-0.025** |
| TAG 56:3 | 0.301 | 0.451 | down | **1.50 ↓** | **-0.150** |
| TAG 56:4 | 0.452 | 0.568 | down | **1.26 ↓** | **-0.116** |
| TAG 56:5 | 0.232 | 0.611 | down | **2.63 ↓** | **-0.378** |
| TAG 56:6 | 0.097 | 0.518 | down | **5.34 ↓** | **-0.421** |
| TAG 56:7 | 0.042 | 0.352 | down | **8.33 ↓** | **-0.310** |
| TAG 56:8 | 0.012 | 0.028 | down | **2.24 ↓** | **-0.015** |
| TAG 56:9 | 0.012 | 0.033 | down | **2.77 ↓** | **-0.021** |
| TAG 58:3 | 0.076 | 0.201 | down | **2.62 ↓** | **-0.124** |
| TAG 58:4 | 0.090 | 0.331 | down | **3.69 ↓** | **-0.241** |
| TAG 58:5 | 0.044 | 0.287 | down | **6.50 ↓** | **-0.243** |
| TAG 58:6 | 0.034 | 0.166 | down | **4.93 ↓** | **-0.132** |

**(B) FAX1ox: TAG content in leaves**: 8/17

| TAG 50:2 | 0.128 | 0.038 | up | **3.36 ↑** | **0.090** |
| --- | --- | --- | --- | --- | --- |
| TAG 50:3 | 0.579 | 0.220 | up | **2.64 ↑** | **0.360** |
| TAG 50:6 | 0.035 | 0.011 | up | **3.28 ↑** | **0.024** |
| TAG 52:4 | 0.905 | 0.594 | up | **1.52 ↑** | **0.311** |
| TAG 52:5 ***** | 1.412 | 0.506 | up | **2.79 ↑** | **0.906** |
| TAG 52:6 ***** | 1.936 | 0.622 | up | **3.11 ↑** | **1.314** |
| TAG 54:4 | 0.489 | 0.602 | down | **1.23 ↓** | **-0.113** |
| TAG 54:7 ***** | 0.790 | 0.564 | up | **1.40 ↑** | **0.227** |
| TAG 56:6 | 0.140 | 0.057 | up | **2.45 ↑** | **0.083** |

**(C) *fax1* ko: TAG content in flowers**: 28/40

| TAG 50:1 | 0.397 | 0.500 | down | **1.26 ↓** | **-0.103** |
| --- | --- | --- | --- | --- | --- |
| TAG 50:2 | 1.184 | 1.334 | down | **1.13 ↓** | **-0.151** |
| TAG 50:3 | 2.256 | 3.597 | down | **1.59 ↓** | **-1.341** |
| TAG 50:4 | 0.182 | 0.203 | down | **1.12 ↓** | **-0.022** |
| TAG 50:6 | 0.160 | 0.174 | down | **1.09 ↓** | **-0.014** |
| TAG 52:1 | 0.168 | 0.238 | down | **1.42 ↓** | **-0.070** |
| TAG 52:3 | 0.932 | 0.864 | up | **1.08 ↑** | **0.068** |
| TAG 52:4 | 2.749 | 3.199 | down | **1.16 ↓** | **-0.451** |
| TAG 52:5 ***** | 3.922 | 3.722 | up | **1.05 ↑** | **0.200** |
| TAG 52:6 ***** | 4.042 | 4.650 | down | **1.15 ↓** | **-0.608** |
| TAG 52:7 | 0.280 | 0.358 | down | **1.28 ↓** | **-0.078** |
| TAG 54:1 | 0.110 | 0.120 | down | **1.09 ↓** | **-0.010** |
| TAG 54:3 | 0.530 | 0.842 | down | **1.59 ↓** | **-0.312** |
| TAG 54:4 | 0.693 | 1.008 | down | **1.45 ↓** | **-0.315** |
| TAG 54:5 | 1.308 | 1.992 | down | **1.52 ↓** | **-0.684** |
| TAG 54:6 | 2.592 | 3.022 | down | **1.17 ↓** | **-0.430** |
| TAG 54:7 ***** | 5.127 | 4.821 | up | **1.06 ↑** | **0.306** |
| TAG 54:8 ***** | 6.439 | 6.965 | down | **1.08 ↓** | **-0.527** |
| TAG 54:9 ***** | 4.200 | 5.131 | down | **1.22 ↓** | **-0.931** |
| TAG 56:1 | 0.053 | 0.063 | down | **1.18 ↓** | **-0.010** |
| TAG 56:6 | 0.383 | 0.939 | down | **2.45 ↓** | **-0.556** |
| TAG 56:7 | 0.278 | 0.548 | down | **1.97 ↓** | **-0.270** |
| TAG 56:8 | 0.225 | 0.215 | up | **1.05 ↑** | **0.010** |
| TAG 56:9 | 0.121 | 0.120 | up | **1.01 ↑** | **0.001** |
| TAG 58:2 | 0.070 | 0.108 | down | **1.55 ↓** | **-0.038** |
| TAG 58:3 | 0.125 | 0.375 | down | **3.00 ↓** | **-0.250** |
| TAG 58:4 | 0.281 | 0.783 | down | **2.78 ↓** | **-0.502** |
| TAG 58:6 | 0.164 | 0.295 | down | **1.80 ↓** | **-0.131** |

**(D) FAX1ox: TAG content in flowers**: 21/40

| TAG 50:5 | 0.123 | 0.096 | up | **1.29 ↑** | **0.027** |
| --- | --- | --- | --- | --- | --- |
| TAG 52:2 | 0.312 | 0.226 | up | **1.38 ↑** | **0.085** |
| TAG 52:3 | 0.852 | 0.669 | up | **1.27 ↑** | **0.183** |
| TAG 52:4 | 2.047 | 1.451 | up | **1.41 ↑** | **0.596** |
| TAG 52:5 ***** | 2.597 | 1.934 | up | **1.34 ↑** | **0.663** |
| TAG 54:2 | 0.195 | 0.150 | up | **1.30 ↑** | **0.045** |
| TAG 54:4 | 1.054 | 0.784 | up | **1.34 ↑** | **0.270** |
| TAG 54:5 | 1.949 | 1.466 | up | **1.33 ↑** | **0.483** |
| TAG 54:6 ***** | 3.606 | 2.536 | up | **1.42 ↑** | **1.070** |
| TAG 54:7 ***** | 4.420 | 3.369 | up | **1.31 ↑** | **1.052** |
| TAG 54:8 ***** | 3.299 | 2.365 | up | **1.39 ↑** | **0.934** |
| TAG 56:2 | 0.030 | 0.012 | up | **2.55 ↑** | **0.019** |
| TAG 56:3 | 0.146 | 0.085 | up | **1.73 ↑** | **0.061** |
| TAG 56:4 | 0.379 | 0.218 | up | **1.74 ↑** | **0.161** |
| TAG 56:5 | 0.580 | 0.235 | up | **2.47 ↑** | **0.345** |
| TAG 56:6 | 0.486 | 0.291 | up | **1.67 ↑** | **0.194** |
| TAG 56:7 | 0.463 | 0.289 | up | **1.60 ↑** | **0.174** |
| TAG 56:8 | 0.213 | 0.131 | up | **1.62 ↑** | **0.081** |
| TAG 56:9 | 0.091 | 0.068 | up | **1.34 ↑** | **0.023** |
| TAG 58:5 | 0.158 | 0.066 | up | **2.40 ↑** | **0.092** |
| TAG 58:6 | 0.101 | 0.024 | up | **4.21 ↑** | **0.077** |
