# Supplementary material for: FAX1, a Novel Membrane Protein Mediating Plastid Fatty Acid Export
Source: PLoS Biol. 2015 Feb 3;13(2):e1002053. doi: 10.1371/journal.pbio.1002053 (PMC4344464; doi:10.1371/journal.pbio.1002053)
Supplement: S5 Table — Depicted are 64 genes (plus two with strongest changes), which according to DNA micorarray analysis are significantly regulated in flower tissue of both fax1 knockout and FAX1 over-expressor lines, and represent genes of acyl lipid metabolism (ARALIP database; http://aralip.plantbiology.msu.edu/; see [1]). Genes also regulated in stems of fax1 knockouts (see S6 Table) are boxed and underlined. Arabidopsis Genome Initiative (AGI) codes and the average scaled signals of mutant and wild type, as well as the fold change (FCH) in flowers of FAX1 knockouts (ko) and over-expressors (ox) are given. Annotation of acyl lipid pathway, protein family, and gene names is according to the ARALIP database. nr: not significantly regulated (DOCX) [file pbio.1002053.s016.docx]

**Table S5. Genes of acyl lipid metabolism, simultaneously regulated in flowers of *FAX1* knockouts and over-expressors.**

|  | **ko_flower** | | | **ox_flower** | | | |  |  |  |  |
| --- | --- | --- | --- | --- | --- | --- | --- | --- | --- | --- | --- |
| **AGI** | **Mu**  **signal** | **Wt signal** | **FCH** | **Mu signal** | **Wt signal** | **FCH** | **Pathway** | | **Protein Family Name** | **Gene Name** | |

**strongest change in ko_flower**

| At5g47350 | 108.18 | 1671.07 | **0.06** | 365.09 | 1535.93 | **0.24** | Pathway. function or subcellular location uncertain | Thioesterase (PPT1-like) |  |
| --- | --- | --- | --- | --- | --- | --- | --- | --- | --- |
| At5g08030 | 27.28 | 195.03 | **0.14** | 64.64 | 199.11 | **0.32** | Pathway. function or subcellular location uncertain | Glycerophosphoryl Diester Phosphodiesterase |  |
| At5g09370 | 70.32 | 422.77 | **0.17** | 112.22 | 300.67 | **0.37** | Fatty Acid Elongation & Wax Biosynthesis | Lipid Transfer Protein | (LTP type 5) |
| At5g39400 | 203.49 | 1063.88 | **0.19** | nr | nr | nr | Phospholipid Signaling | Phosphoinositide 3-Phosphatase | PTEN1 |

**strongest change in ox_flower**

| Atcg00500 | nr | nr | nr | 1044.61 | 235.58 | **4.43** | Fatty Acid Synthesis | Carboxyltransferase ß-subunit of heteromeric ACCase | ß-CT |
| --- | --- | --- | --- | --- | --- | --- | --- | --- | --- |
| At5g47350 | 108.18 | 1671.07 | **0.06** | 365.09 | 1535.93 | **0.24** | Pathway. function or subcellular location uncertain | Thioesterase (PPT1-like) |  |
| At5g07560 | 932.65 | 2752.69 | **0.34** | 599.94 | 2131.36 | **0.28** | Triacylglycerol Biosynthesis | Pollen-surface Oleosin |  |
| At4g08670 | 295.76 | 1299.75 | **0.23** | 334.03 | 1134.62 | **0.29** | Fatty Acid Elongation & Wax Biosynthesis | Lipid Transfer Protein | (LTP type 5) |

**reciprocal regulation**

| At4g34250 | 120.27 | 90.25 | **1.33** | 65.60 | 81.81 | **0.80** | Fatty Acid Elongation & Wax Biosynthesis | Ketoacyl-CoA Synthase | KCS16 |
| --- | --- | --- | --- | --- | --- | --- | --- | --- | --- |

**up-regulated**

| At1g64670 | 914.35 | 721.61 | **1.27** | 850.01 | 666.68 | **1.27** | Cutin/Suberin Synthesis & Transport 1 | Hydrolase-like Protein | BDG | |
| --- | --- | --- | --- | --- | --- | --- | --- | --- | --- | --- |
| At5g22420 | 107.93 | 54.31 | **1.99** | 69.63 | 48.41 | **1.44** | Fatty Acid Elongation & Wax Biosynthesis | Alcohol-forming Fatty Acyl-CoA Reductase | BDG | |
| At5g60340 | 67.90 | 48.27 | **1.41** | 48.76 | 39.11 | **1.25** | Mitochondrial Fatty Acid & Lipoic Acid Synthesis | Hydroxyacyl-ACP Dehydrase | AlcFAR7 | |
| At3g16170 | 579.43 | 492.07 | **1.18** | 484.99 | 362.82 | **1.34** | Mitochondrial Fatty Acid & Lipoic Acid Synthesis | Malonyl-CoA Synthase | HAD |  |
| At4g37050 | 288.29 | 206.71 | **1.39** | 195.49 | 148.51 | **1.32** | Oxylipin Metabolism 1 | Acyl-Hydrolase (Patatin-like) | MCS/AAE13 | |
| At3g25760 | 261.04 | 78.81 | **3.31** | 130.61 | 70.76 | **1.85** | Oxylipin Metabolism 1; Oxylipin Metabolism 2 | Allene Oxide Cyclase |  | |
| At3g14205 | 736.85 | 557.03 | **1.32** | 676.40 | 477.02 | **1.42** | Phospholipid Signaling | Sac domain-containing Phosphoinositide Phosphatase |  | |
| At3g25610 | 107.90 | 84.97 | **1.27** | 87.56 | 68.75 | **1.27** | Pathway. function or subcellular location uncertain | Translocase |  | |

**down-regulated**

| At3g03540 | 69.08 | 131.34 | **0.53** | 39.48 | 83.83 | **0.47** | Eukaryotic Galactolipid & Sulfolipid Synthesis | Phospholipase C (Non specific) | NPC5 |
| --- | --- | --- | --- | --- | --- | --- | --- | --- | --- |
| At4g11030 | 40.22 | 108.55 | **0.37** | 33.61 | 83.40 | **0.40** | Eukaryotic Phospholipid Synthesis & Editing | Long-Chain Acyl-CoA Synthetase | LACS5 |
| At4g38570 | 510.52 | 651.04 | **0.78** | 516.78 | 619.08 | **0.83** | Eukaryotic Phospholipid Synthesis & Editing | Phosphatidylinositol Synthase | PIS2 |
| At1g80460 | 1080.22 | 1314.99 | **0.82** | 884.00 | 1041.08 | **0.85** | Eukaryotic Phospholipid Synthesis & Editing | Glycerol Kinase | GLI1 |
| At5g52160 | 278.56 | 1127.84 | **0.25** | 523.65 | 991.45 | **0.53** | Fatty Acid Elongation & Wax Biosynthesis | Lipid Transfer Protein | (LTP type 3) |
| At5g62080 | 788.92 | 2647.72 | **0.30** | 1307.80 | 2558.93 | **0.51** | Fatty Acid Elongation & Wax Biosynthesis | Lipid Transfer Protein | (LTP type 3) |
| At1g18280 | 430.65 | 1439.97 | **0.30** | 449.74 | 1202.10 | **0.37** | Fatty Acid Elongation & Wax Biosynthesis | Lipid Transfer Protein | (LTP type 5) |
| At1g73560 | 36.28 | 87.21 | **0.42** | 34.53 | 71.10 | **0.49** | Fatty Acid Elongation & Wax Biosynthesis | Lipid Transfer Protein | (LTP type 5) |
| At1g36150 | 97.26 | 182.37 | **0.53** | 87.96 | 181.84 | **0.48** | Fatty Acid Elongation & Wax Biosynthesis | Lipid Transfer Protein | (LTP type 5) |
| At3g07450 | 306.29 | 885.32 | **0.35** | 415.54 | 760.26 | **0.55** | Fatty Acid Elongation & Wax Biosynthesis | Lipid Transfer Protein | (LTP type 3) |
| At2g27130 | 469.18 | 640.99 | **0.73** | 412.78 | 508.77 | **0.81** | Fatty Acid Elongation & Wax Biosynthesis | Lipid Transfer Protein | (LTP type 5) |
| At3g52130 | 291.25 | 747.65 | **0.39** | 298.68 | 684.40 | **0.44** | Fatty Acid Elongation & Wax Biosynthesis | Lipid Transfer Protein | (LTP type 3) |
| At1g66850 | 5938.48 | 12365.67 | **0.48** | 7454.70 | 11837.91 | **0.63** | Fatty Acid Elongation & Wax Biosynthesis | Lipid Transfer Protein | (LTP type 2) |
| At5g01870 | 455.46 | 1153.39 | **0.39** | 707.68 | 1034.84 | **0.68** | Fatty Acid Elongation & Wax Biosynthesis | Lipid Transfer Protein | (LTP type 1) |
| At4g28395 | 930.39 | 2071.49 | **0.45** | 797.62 | 1596.73 | **0.50** | Fatty Acid Elongation & Wax Biosynthesis | Lipid Transfer Protein | ATA7 / (LTP type 8) |
| At3g52160 | 232.68 | 560.47 | **0.42** | 334.15 | 609.23 | **0.55** | Fatty Acid Elongation & Wax Biosynthesis | Ketoacyl-CoA Synthase | KCS15 |
| At3g23840 | 325.40 | 690.38 | **0.47** | 307.89 | 635.87 | **0.48** | Fatty Acid Elongation & Wax Biosynthesis | CER2-like Protein |  |
| At5g55320 | 60.29 | 93.57 | **0.64** | 42.86 | 60.76 | **0.71** | Fatty Acid Elongation & Wax Biosynthesis | Wax Synthase |  |
| At3g55100 | 75.63 | 132.80 | **0.57** | 65.80 | 104.59 | **0.63** | Fatty Acid Elongation & Wax Biosynthesis | ABC Transporter | WBC17 / ABCG17 |
| At5g13580 | 216.49 | 328.27 | **0.66** | 180.98 | 319.00 | **0.57** | Fatty Acid Elongation & Wax Biosynthesis  Suberin Synthesis & Transport 3 | ABC Transporter | WBC6 / ABCG6 |
| At1g06250 | 94.15 | 169.68 | **0.55** | 58.37 | 119.71 | **0.49** | Oxylipin Metabolism 1; Oxylipin Metabolism 2 | Acylhydrolase (DAD1-like) |  |
| At1g02660 | 363.58 | 608.05 | **0.60** | 327.83 | 537.41 | **0.61** | Oxylipin Metabolism 1; Oxylipin Metabolism 2 | Lipid Acylhydrolase-like |  |
| At1g73680 | 358.05 | 536.74 | **0.67** | 341.67 | 472.36 | **0.72** | Oxylipin Metabolism 2 | α-Dioxygenase-Peroxidase (involved in fatty acid α-oxidation) |  |
| At1g52570 | 184.75 | 459.16 | **0.40** | 193.80 | 409.75 | **0.47** | Phospholipid Signaling | Phospholipase D alpha |  |
| At5g57690 | 89.17 | 209.42 | **0.43** | 116.19 | 196.25 | **0.59** | Phospholipid Signaling | Diacylglycerol Kinase |  |
| At5g66020 | 77.14 | 176.60 | **0.44** | 72.11 | 136.52 | **0.53** | Phospholipid Signaling | Sac domain-containing Phosphoinositide Phosphatase |  |
| At2g41210 | 117.75 | 246.79 | **0.48** | 122.98 | 210.16 | **0.59** | Phospholipid Signaling | Phosphatidylinositol-Phosphate Kinase type IB |  |
| At1g64460 | 197.63 | 280.05 | **0.71** | 204.76 | 261.34 | **0.78** | Phospholipid Signaling | Phosphatidylinositol-4-Kinase gamma |  |
| At4g26770 | 36.89 | 69.09 | **0.53** | 32.71 | 55.36 | **0.59** | Pro. Galactolipid. Sulfolipid. & Phospholipid Synt. 1  Mitochondrial Phospholipid Synthesis | CDP-DAG Synthase | CDS3/CDP-DAGS |
| At5g20410 | 397.05 | 1332.82 | **0.30** | 725.29 | 1198.33 | **0.61** | Pro. Galactolipid. Sulfolipid. & Phospholipid Synt. 2  Euk. Galactolipid & Sulfolipid Synthesis | Monogalactosyldiacylglycerol Synthase | MGD2 |
| At2g46210 | 610.48 | 2569.78 | **0.24** | 1121.18 | 1757.65 | **0.64** | Sphingolipid Biosynthesis 1 | Sphingobase-D8 Desaturase | SLD2 |
| At4g04930 | 408.06 | 1594.41 | **0.26** | 829.95 | 1546.09 | **0.54** | Sphingolipid Biosynthesis 1 | Dihydrosphingosine Delta-4 Desaturase | DSD1 |
| At4g20870 | 1226.84 | 1718.98 | **0.71** | 1140.14 | 1510.77 | **0.75** | Sphingolipid Biosynthesis 1 | Fatty Acid 2-hydroxylase | FAH2 |
| At4g39670 | 323.13 | 1051.85 | **0.31** | 347.75 | 757.58 | **0.46** | Sphingolipid Biosynthesis 1;2 | Sphingosine Transfer Protein |  |
| At3g11430 | 864.62 | 1351.18 | **0.64** | 672.96 | 1034.10 | **0.65** | Suberin Synthesis & Transport 1 | Glycerol-3-Phosphate Acyltransferase | sn-2-GPAT5 |
| At3g55180 | 76.91 | 223.87 | **0.34** | 102.62 | 203.30 | **0.50** | Triacylglycerol & Fatty Acid Degradation | Monoacylglycerol Lipase (MAGL) |  |
| At3g55190 | 22.62 | 60.42 | **0.37** | 27.82 | 60.86 | **0.46** | Triacylglycerol & Fatty Acid Degradation | Monoacylglycerol Lipase (MAGL) | SDP1-LIKE |
| At3g57140 | 38.58 | 113.73 | **0.34** | 65.69 | 102.84 | **0.64** | Triacylglycerol & Fatty Acid Degradation;  Oxylipin Metabolism 1 | Triacylglycerol Lipase (TAGL) |  |
| At2g25890 | 72.45 | 262.81 | **0.28** | 85.72 | 274.66 | **0.31** | Triacylglycerol Biosynthesis | Oil-Body Oleosin |  |
| At3g18570 | 87.99 | 178.18 | **0.49** | 88.54 | 171.48 | **0.52** | Triacylglycerol Biosynthesis | Oil-Body Oleosin |  |
| At5g07510 | 316.55 | 767.51 | **0.41** | 290.08 | 536.44 | **0.54** | Triacylglycerol Biosynthesis | Pollen-surface Oleosin |  |
| At5g07540 | 602.11 | 1219.27 | **0.49** | 345.23 | 1107.87 | **0.31** | Triacylglycerol Biosynthesis | Pollen-surface Oleosin |  |
| At5g07530 | 4443.58 | 8718.73 | **0.51** | 3976.09 | 7545.64 | **0.53** | Triacylglycerol Biosynthesis | Pollen-surface Oleosin |  |
| At5g07520 | 245.72 | 481.52 | **0.51** | 185.68 | 426.34 | **0.44** | Triacylglycerol Biosynthesis | Pollen-surface Oleosin |  |
| At5g07550 | 6545.73 | 11367.27 | **0.58** | 4824.89 | 9493.73 | **0.51** | Triacylglycerol Biosynthesis | Pollen-surface Oleosin |  |
| At1g23250 | 101.47 | 165.98 | **0.61** | 102.77 | 148.73 | **0.69** | Triacylglycerol Biosynthesis | Caleosin |  |
| At3g15820 | 1187.12 | 1646.36 | **0.72** | 1150.52 | 1353.21 | **0.85** | Triacylglycerol Biosynthesis | Phosphatidylcholine:diacylglycerol cholinephosphotransferase | PDCT/ROD1 |
| At1g51260 | 52.91 | 133.89 | **0.40** | 89.20 | 150.17 | **0.59** | Triacylglycerol Biosynthesis  Euk. Phospholipid Synthesis & Editing | 1-Acylglycerol-3-Phosphate Acyltransferase | LPAAT3 |
| At4g17480 | 405.96 | 593.73 | **0.68** | 374.30 | 530.74 | **0.71** | Pathway. function or subcellular location uncertain | Thioesterase (PPT1-like) |  |
| At1g54280 | 58.83 | 181.00 | **0.33** | 96.73 | 170.75 | **0.57** | Pathway. function or subcellular location uncertain | Translocase |  |
