# Supplementary material for: FAX1, a Novel Membrane Protein Mediating Plastid Fatty Acid Export
Source: PLoS Biol. 2015 Feb 3;13(2):e1002053. doi: 10.1371/journal.pbio.1002053 (PMC4344464; doi:10.1371/journal.pbio.1002053)
Supplement: S6 Table — Depicted are 58 genes, which according to DNA micorarray analysis are significantly regulated in stem tissue of fax1 knockout mutants, and represent genes of acyl lipid metabolism (ARALIP database; http://aralip.plantbiology.msu.edu/; see [1]). Genes also regulated in FAX1 mutant flowers (see S5 Table) are boxed and underlined. Arabidopsis Genome Initiative (AGI) codes and the average scaled signals of mutant and wild type as well as the fold change (FCH) in flowers of fax1 knockouts (ko) are given. Annotation of acyl lipid pathway, protein family and gene names is according to the ARALIP database. (DOCX) [file pbio.1002053.s017.docx]

**Table S6. Genes of acyl lipid metabolism, regulated in stem tissue of *fax1* knockout mutants.**

|  | **ko_stem** | | |  |  |  |
| --- | --- | --- | --- | --- | --- | --- |
| **AGI** | **Mu**  **signal** | **Wt signal** | **FCH** | **Pathway** | **Protein Family Name** | **Gene Name** |

**strongest change in ko_stem**

| At1g57750 | 1911.17 | 193.67 | **9.87** | Fatty Acid Elongation & Wax Biosynthesis | Secondary Alcohol Dehydrogenase; Midchain Alkane Hydroxylase | CYP96A15/MAH1 |
| --- | --- | --- | --- | --- | --- | --- |
| At4g33790 | 619.85 | 115.02 | **5.39** | Fatty Acid Elongation & Wax Biosynthesis;  Suberin Synthesis & Transport 1 | Alcohol-forming Fatty Acyl-CoA Reductase | AlcFAR3/CER4 |
| At2g13820 | 1074.19 | 225.61 | **4.76** | Fatty Acid Elongation & Wax Biosynthesis | Lipid Transfer Protein | (LTP type 5) |
| At1g06350 | 181.93 | 39.58 | **4.60** | Pathway, function or subcellular location uncertain | Acyl-CoA Desaturase-like / FAD5-like Desaturase |  |

**up-regulated**

| At3g48720 | 257.98 | 107.80 | **2.39** | Cutin Synthesis & Transport 1 | Feruloyl Transferase | DCF |
| --- | --- | --- | --- | --- | --- | --- |
| At4g00400 | 90.57 | 41.28 | **2.19** | Cutin Synthesis & Transport 1 | Glycerol-3-Phosphate Acyltransferase | sn-2-GPAT8 |
| At2g26910 | 680.53 | 447.66 | **1.52** | Cutin Synthesis & Transport 1 | ABC Transporter | ABCG32/PEC1 |
| At3g03540 | 35.31 | 16.06 | **2.20** | Eukaryotic Galactolipid & Sulfolipid Synthesis | Phospholipase C (Non specific) | NPC5 |
| At3g18000 | 253.24 | 105.28 | **2.41** | Eukaryotic Phospholipid Synthesis & Editing | Phosphoethanolamine N-Methyltransferase | NMT1 |
| At3g05420 | 582.57 | 341.03 | **1.71** | Eukaryotic Phospholipid Synthesis & Editing | Acyl CoA Binding Protein | ACBP4 |
| At1g55260 | 94.84 | 22.00 | **4.31** | Fatty Acid Elongation & Wax Biosynthesis | Lipid Transfer Protein | (LTP type 5) |
| At1g62790 | 985.90 | 456.44 | **2.16** | Fatty Acid Elongation & Wax Biosynthesis | Lipid Transfer Protein | (LTP type 5) |
| At3g43720 | 764.16 | 371.95 | **2.05** | Fatty Acid Elongation & Wax Biosynthesis | Lipid Transfer Protein | (LTP type 5) |
| At2g27130 | 618.57 | 345.45 | **1.79** | Fatty Acid Elongation & Wax Biosynthesis | Lipid Transfer Protein | (LTP type 5) |
| At4g24510 | 389.75 | 204.92 | **1.90** | Fatty Acid Elongation & Wax Biosynthesis | CER2-like Protein | CER2 |
| At1g10670 | 3952.44 | 1453.62 | **2.72** | Fatty Acid Elongation & Wax Biosynthesis | ATP Citrate Lyase A subunit | ACLA-1 |
| At1g60810 | 1654.10 | 391.95 | **4.22** | Fatty Acid Elongation & Wax Biosynthesis | ATP Citrate Lyase A subunit | ACLA-2 |
| At3g06650 | 1761.47 | 1092.56 | **1.61** | Fatty Acid Elongation & Wax Biosynthesis | ATP Citrate Lyase B subunit | ACLB-1 |
| At5g49460 | 2327.85 | 622.54 | **3.74** | Fatty Acid Elongation & Wax Biosynthesis | ATP Citrate Lyase B subunit | ACLB-2 |
| At2g47240 | 230.37 | 74.51 | **3.09** | Fatty Acid Elongation & Wax Biosynthesis;  Cutin/Suberin Synthesis & Transport 1 | Long-Chain Acyl-CoA Synthetase | LACS1 |
| At1g64400 | 1221.43 | 556.37 | **2.20** | Fatty Acid Elongation & Wax Biosynthesis;  Cutin/Suberin Synthesis & Transport 1 | Long-Chain Acyl-CoA Synthetase | LACS3 |
| At5g25390 | 178.10 | 101.40 | **1.76** | Fatty Acid Elongation & Wax Biosynthesis;  Cutin/Suberin Synthesis & Transport 1 | SHN Transcription Factors | SHN3 |
| At1g19440 | 316.09 | 201.47 | **1.57** | Fatty Acid Elongation & Wax Biosynthesis;  Suberin Synthesis & Transport 1 | Ketoacyl-CoA Synthase | KCS4 |
| At5g16230 | 54.86 | 34.83 | **1.58** | Fatty Acid Elongation, Desaturation & Export From Plastid;  Fatty Acid Synthesis;  Pro. Galactolipid, Sulfolipid, & Phospholipid Synthesis 1 | Stearoyl-ACP Desaturase | DES3 |
| At4g16155 | 224.24 | 125.69 | **1.78** | Fatty Acid Synthesis | Dihydrolipoamide Dehydrogenase,  E3 component of Pyruvate Dehydrogenase Complex | LPD2 (E3) |
| At1g63430 | 493.58 | 339.81 | **1.45** | Mitochondrial Phospholipid Synthesis | CDP-DAG Synthase | CDS1; CDP-DAGS |
| At3g63200 | 208.03 | 68.92 | **3.02** | Oxylipin Metabolism 1 | Acyl-Hydrolase (Patatin-like) |  |
| At3g08510 | 1643.86 | 1189.24 | **1.38** | Phospholipid Signaling | Phosphoinositide-specific Phospholipase C |  |
| At4g00240 | 95.21 | 72.07 | **1.32** | Phospholipid Signaling | Phospholipase D beta |  |
| At4g20870 | 656.25 | 337.76 | **1.94** | Sphingolipid Biosynthesis 1 | Fatty Acid 2-hydroxylase | FAH2 |
| At3g06060 | 444.72 | 301.83 | **1.47** | Sphingolipid Biosynthesis 1 | Ketosphinganine Reductase | TSC10A |
| At2g40890 | 3124.42 | 1307.77 | **2.39** | Suberin Synthesis & Transport 2 | Coumaroyl 3-Hydroxylase | CYP98A3 |
| At2g30490 | 8521.15 | 3903.89 | **2.18** | Suberin Synthesis & Transport 2 | Cinnamate 4-Hydroxylase | CYP73A5 |
| At4g34050 | 15751.28 | 7257.13 | **2.17** | Suberin Synthesis & Transport 2 | Caffeoyl-CoA O-Methyltransferase |  |
| At4g14440 | 390.23 | 176.59 | **2.21** | Triacylglycerol & Fatty Acid Degradation | Enoyl CoA isomerase | ECI3 |
| At4g32010 | 814.61 | 472.90 | **1.72** | Triacylglycerol Biosynthesis | a member of a novel family of B3 domain proteins | HSL1/VAL2 |
| At2g29980 | 1486.81 | 535.98 | **2.77** | Triacylglycerol Biosynthesis; Euk. Phospholipid Synthesis & Editing | Linoleate Desaturase | FAD3 |
| At3g18850 | 195.48 | 73.54 | **2.66** | Triacylglycerol Biosynthesis; Euk. Phospholipid Synthesis & Editing | 1-Acylglycerol-3-Phosphate Acyltransferase | LPAAT5 |
| At5g47730 | 844.73 | 444.99 | **1.90** | Pathway, function or subcellular location uncertain | Sec14-like Protein |  |

**down-regulated**

| At4g01950 | 50.04 | 106.16 | **0.47** | Cutin Synthesis & Transport 1 | Glycerol-3-Phosphate Acyltransferase | GPAT3 |
| --- | --- | --- | --- | --- | --- | --- |
| At3g05630 | 51.79 | 84.00 | **0.62** | Eukaryotic Galactolipid & Sulfolipid Synthesis | Phospholipase D zeta | PLD zeta2 |
| At5g06530 | 3735.37 | 6032.49 | **0.62** | Fatty Acid Elongation & Wax Biosynthesis | ABC Transporter | WBC23 / ABCG22 |
| At4g34250 | 21.08 | 31.52 | **0.67** | Fatty Acid Elongation & Wax Biosynthesis | Ketoacyl-CoA Synthase | KCS16 |
| At2g26560 | 34.20 | 97.29 | **0.35** | Oxylipin Metabolism 1 | Acyl-Hydrolase (Patatin-like) |  |
| At1g76690 | 885.61 | 1542.35 | **0.57** | Oxylipin Metabolism 1 | Oxo-Phytodienoic Acid Reductase |  |
| At5g24210 | 389.27 | 942.16 | **0.41** | Oxylipin Metabolism 1; Oxylipin Metabolism 2 | Lipid Acylhydrolase-like |  |
| At5g42650 | 923.39 | 1430.32 | **0.65** | Oxylipin Metabolism 1; Oxylipin Metabolism 2 | Allene Oxide Synthase |  |
| At1g73680 | 573.59 | 873.55 | **0.66** | Oxylipin Metabolism 2 | alpha-Dioxygenase-Peroxidase (involved in fatty acid alpha-oxidation) |  |
| At5g39400 | 19.42 | 29.38 | **0.66** | Phospholipid Signaling | Phosphoinositide 3-Phosphatase | PTEN1 |
| At3g18220 | 25.02 | 34.65 | **0.72** | Pro. Galactolipid, Sulfolipid, & Phospholipid Synthesis 1;  Euk. Galactolipid & Sulfolipid Synthesis;  Phospholipid Signaling | Phosphatidate Phosphatase /  Diacylglycerol-Pyrophosphate Phosphatase | LPP4 |
| At5g01220 | 285.43 | 486.91 | **0.59** | Pro. Galactolipid, Sulfolipid, & Phospholipid Synthesis 2;  Euk. Galactolipid & Sulfolipid Synthesis | UDP-sulfoquinovose:DAG sulfoquinovosyltransferase | SQD2 |
| At1g69640 | 483.29 | 815.40 | **0.59** | Sphingolipid Biosynthesis 1 | Sphingobase C4-Hydroxylase | SBH1 |
| At4g04930 | 21.34 | 33.43 | **0.64** | Sphingolipid Biosynthesis 1 | Dihydrosphingosine Delta-4 Desaturase | DSD1 |
| At1g73480 | 692.89 | 1867.35 | **0.37** | Triacylglycerol & Fatty Acid Degradation | Monoacylglycerol Lipase (MAGL) |  |
| At1g23330 | 261.05 | 461.22 | **0.57** | Triacylglycerol & Fatty Acid Degradation | Triacylglycerol Lipase (TAGL) |  |
| At5g14930 | 68.61 | 110.41 | **0.62** | Triacylglycerol & Fatty Acid Degradation | Triacylglycerol Lipase (TAGL) | SAG101 |
| At1g76150 | 1060.49 | 1440.39 | **0.74** | Triacylglycerol & Fatty Acid Degradation | Peroxisomal Enoyl-CoA Hydratase 2 | ECH2 |
| At3g51970 | 53.00 | 102.58 | **0.52** | Pathway, function or subcellular location uncertain | Membrane-bound O-acyltransferase | ACAT1 |
