# Supplementary material for: FAX1, a Novel Membrane Protein Mediating Plastid Fatty Acid Export
Source: PLoS Biol. 2015 Feb 3;13(2):e1002053. doi: 10.1371/journal.pbio.1002053 (PMC4344464; doi:10.1371/journal.pbio.1002053)
Supplement: S7 Table — (DOCX) [file pbio.1002053.s018.docx]

**Table S7. Oligonucleotides used in this study**

| **PCR genotyping** |  |
| --- | --- |
| At-fax1-2 LP | 5'-AAGGAAACCTAAGCTTAAACCAGC-3' |
| At-fax1 LP | 5'-TTTCTTCGCAACATTTTGACC-3' |
| LB1 SAIL | 5'-GCCTTTTCAGAAATGGATAAATAGCCTTGCTTCC-3' |
| GABI LB1 | 5'-ATAATAACGCTGCGGACATCTACATTTT-3' |
| At-fax1 RP2 | 5'-AGTGGAGACACTATCAATCCC-3' |
| At-fax1 RP | 5'-CCTCTACTGGCTCTGTGATGC-3' |
| **Quantitative real time RT-PCR** | |
| At-FAX1 LC fw | 5'-CCTATGATTCGTCCCCAG-3' |
| At-FAX1 LC rev | 5'-CACTCACAACGAGACCA-3' |
| **Isolation of Ps-FAX1 cDNA** | |
| Ps-fax1 fw | 5'-TACTTCGCCGGACAGGTTCGTGAACG-3' |
| Ps-fax1 rev | 5'-CATCGTCGAACCAAAATACATGTGCCG-3' |
| **Subcloning of At-FAX1 cDNA** | |
| At-FAX1 fw | 5'-CACCATGGCTTCACAAATCTCTCAGC-3' |
| At-FAX1 rev (-stop) | 5'-GTATGAAGGACTAGTCGCAGATGG-3' |
| At-FAX1 rev | 5'-TCAGTATGAAGGACTAGTCGCAGATGG-3' |
| matFAX1/pDR195 fw | 5'-ctcgagatgtcttttgtagttaaaagtg-3' |
| matFAX1/pDR195 rev | 5'-GGATCCTCAGTATGAAGGACTAGTCGCAGA-3' |
