# Supplementary material for: FAX1, a Novel Membrane Protein Mediating Plastid Fatty Acid Export
Source: PLoS Biol. 2015 Feb 3;13(2):e1002053. doi: 10.1371/journal.pbio.1002053 (PMC4344464; doi:10.1371/journal.pbio.1002053)
Supplement: S1 Text — (DOCX) [file pbio.1002053.s019.docx]

**Text S1. Differential gene expression in FAX1 mutants.**

To identify effects of *FAX1* mutation on the transcript level, we monitored differential gene expression by DNA microarray analysis (Affymetrix ATH1 GeneChip) in flower and stem tissue of *fax1* knockout, *FAX1* over-expressor, and corresponding wild-type lines. In comparison to wild type, we determined 3346 significantly regulated genes (p-value ≤ 0.05) in *fax1* knockout flowers (Figure S7A), 2366 in flowers of FAX1 over-expressors (Figure S7B) and 1947 in *fax1* knockout stems (Figure S7C), respectively. All microarray data are available in the ArrayExpress database (www.ebi.ac.uk/arrayexpress) under accession number E-MTAB-3090.

Differential Expression of Acyl Lipid Metabolism Genes in *FAX1* Mutants

To spotlight FAX1 function in FA-transport and lipid homeostasis, we first generated overlaps of regulated genes from *FAX1* mutants with genes involved in acyl lipid metabolism (ARALIP database: http://aralip.plantbiology.msu.edu/, [1]; Table S5, Table S6). When we analyzed overlaps with the ARALIP database in flower tissue, we found that 131 genes ‑ regulated in *fax1* knockout ‑, and 99 genes ‑ differentially expressed in *FAX1* over-expressors ‑ have a function in acyl lipid metabolism, thereby covering 17.8% and 13.5% of the genes represented in this functional category, respectively. In total, 1171 genes were simultaneously regulated in flowers of both *fax1* knockouts and *FAX1* over-expressors (Figure S7A,B), including 64 genes present in ARALIP (Table S5). The strongest regulation in *fax1* knockouts showed a putative palmitoyl protein thioesterase (At5g47350; FCH = 0.06), most likely targeted to the secretory pathway. In *FAX1* over-expressors, transcripts for At5g47350 were also decreased (2^nd^ strongest change; FCH = 0.24), but the highest change (increase by factor 4.43) was for AtCg00500 (ß-CT/accD), the plastid encoded carboxyltransferase ß-subunit of the heteromeric ACCase (acetyl-CoA carboxylase) complex, involved in the 2^nd^ step of plastid intrinsic FA-synthesis (for overview, see [1]). Thus, the latter finding is well in line with increased plastid FA-synthesis, due to increased FA-export via over-expressed FAX1 proteins in flower tissue.

A focus of regulation in *fax1* knockout flowers was for genes functioning in phospholipid signaling (22 genes, see E-MTAB-3090 at www.ebi.ac.uk/arrayexpress), including the strong down-regulation of PTEN1 (At5g39400; FCH = 0.19; Table S5) a phosphoinositide 3-phosphatase [2]. Since transcripts for PTEN1 were also reduced in *fax1* knockout stems (see Table S6), this phosphatase might play a general role in signaling associated with the loss of FAX1 function. Interestingly only one gene, KCS16, functioning in fatty acid elongation and wax biosynthesis, was reciprocally regulated in the overlap with ARALIP, i.e. down in *FAX1* over-expressors, but up-regulated in *fax1* knockouts (Table S5). For the majority of genes (55), however, the transcript content decreased in flower tissue of both mutant lines. Here, one focus was on genes described to function in FA elongation and wax biosynthesis (18), mostly represented by lipid transfer proteins (13), which also were among those genes with the strongest differential expression. A second focus was on triacylglycerol biosynthesis (11 genes), including 8 oleosin genes down-regulated in flowers of *fax1* knockouts and over-expressors (Table S5). Oleosins are amphipathic structural proteins, which stabilize triacylglycerol granules in seeds and tapetum cells of anthers. Since 6 of the down-regulated oleosin proteins are specifically expressed in tapetum cells [3], their differential expression in *FAX1* mutants might reflect the observed "sticky pollen" phenotype in *fax1* knockout anthers (compare Figure 4).

For the comparison of *fax1* knockouts *versus* wild type in stem tissue (Figure S7C), 58 genes were represented in the ARALIP database (Table S6). In general, most genes (39) of acyl lipid metabolism were up-regulated. Here, the strongest fold change for CYP96A15/MAH1 (At1g57750; FCH = 9.87), and the highest portion of genes (10), belonged to the FA-elongation and wax biosynthesis category. In the latter pathway CYP96A15/MAH1, a midchain alkane hydroxylase, catalyses the two last steps in formation of ketone wax components, most likely in the ER [4]. Thus, this finding is well in line with the observed reduction of wax C29 ketones in *fax1* knockout stems (compare Figure 5). The second strongest regulated gene in *fax1* knockout stems, the alcohol forming fatty acyl CoA reductase AlcFAR3/CER4, also functions in ER wax biosynthesis two steps ahead of CYP96A15/MAH1 (for overview, see [1]). In particular AlcFAR3/CER4 produces fatty alcohols (C_24:0_-C_30:0_) for synthesis of cuticular wax esters.

Although we did not perform an in depth analysis of differential gene expression in stem tissue of *FAX1* over-expressors, we interestingly found transcripts for a plastid-localized fatty acyl CoA reductase, AlcFAR6 (At3g56700), to be highly increased (FCH = 30.29, p-value < 0.5; mean signals of 2177 and 72 in FAX1ox#4 (n = 2) and wild type (n = 2), respectively). . Since AlcFAR6 has been shown to produce C16:0 fatty alcohols in plastids [5], it is tempting to speculate that in addition to FA-transport, FAX1 might be involved in plastid export of fatty alcohols as well. Moreover, the very similar pollen phenotypes of knockouts for *fax1* (see Figure 4, Discussion) and a second plastid-intrinsic fatty-acyl-ACP reductase (AlcFAR2/MS2) that is involved in primary fatty alcohol synthesis for anther cuticle and pollen sporopollenin formation [6], underlines this hypothesis.

Differential Gene Expression in *FAX1* Mutants Affects Carbon Metabolism

To further identify hot spots of gene regulation covering all cellular functions, we assigned differentially regulated genes to functional categories according to MAPMAN, based on TAIR10 annotation (Ath_AFFY_ATH1_TAIR10_ Aug2012; http://mapman.gabipd.org). Therefore, we sub-divided the TAIR10 functional categories into portions containing between 50-600 genes (exceptions 35.1_"not assigned.no ontology": 1298; 35.2_"not assigned.unknown": 5407 genes, respectively). Except for lipid metabolism (detailed comparison with ARALIP, see above), we further selected only those categories, which contained more than 10% and 7.5% of significantly regulated genes in *fax1* knockout flowers (Figure S8) and flowers of FAX1 over-expressors (Figure S9), stems of *fax1* knockouts (Figure S10), respectively. Here, the major focus of regulation over all three comparisons concerned carbohydrate and cellular/cell wall metabolism, as specified in the following (for more details, see E-MTAB-3090 at www.ebi.ac.uk/arrayexpress). In flowers of *FAX1* knockouts and over-expressors (Figure S8, Figure S9) first of all, genes involved in the light-reaction of photosynthesis were up-regulated (about 2-times stronger in over-expressors). Further, genes of major and minor CHO metabolism and glycolysis (functional categories 2., 3., 4.) were regulated in all three comparisons. Interestingly, in flowers of *fax1* knockouts, the plastid branch of glycolysis was induced, whereas in over-expressors, the cytosolic branch was repressed. Several categories of cell wall metabolism, predominantly associated with modification of polysaccharides and thus as well linked to carbohydrate metabolism, were highly adjusted on the transcript level in *FAX1* mutants. In flower tissue of *FAX1* knockouts and over-expressors, genes for cell wall synthesis, degradation, cell wall proteins, and cell modification (sub classes of category 10) were mainly down-regulated whereas they were induced in *fax1* knockout stems (Figure S8, Figure S9, Figure S10). The strongest down-regulation in flowers, however, was observed for pectin esterases (10.8.1) and invertase/pectin esterase inhibitors (26.18). Only in stem tissue (Figure S10), we saw an induction of expression for UDP glycosyl/glucuronyl transferases (26.2), which are involved in synthesis of cell wall polysaccharides. Further, glycosyl hydrolases that degrade complex sugars such as cellulose were repressed in flowers of *FAX1* mutants, but induced in stems (numbers 26.3 gluco-, galacto-, mannosidases; 26.4 ß-glucan hydrolases).

In summary, in stem tissue cell wall associated processes were stimulated, which is very well reflected by the strong morphological changes we observed in microscopic analysis (compare Figure 5, Figure S2), and by the differences in biomass, e.g. stem dry weight (Table 1). In contrast, the same functional categories in flowers were repressed. This interpretation is underlined by the finding that only in *fax1* knockout stems, genes for cell organization, cell division, cell cycle, and cellular vesicle transport (categories 31.1-4, Figure S10) were almost exclusively up-regulated.

Another focus of differential gene expression, present in all three comparisons, was for genes coding for transporters (category 34, Figure S8, Figure S9, Figure S10). In flowers of *FAX1* mutants most of these transporter genes were down-regulated. In *fax1* knockout flowers the most intense impact was for P-, V-type ATPases (23.2%), sugar (23.9%), and metal (24.7%) transporters, whereas in flowers of *FAX1* over-expressors many genes for metal (21.4%) and peptide/oligopeptide (22%) transporters were affected. In *fax1* stems instead, only genes for amino acid/ammonium transporters were down-regulated.

For lipid associated processes, besides those genes represented in the ARALIP database (see above), we found expression of GDSL-motif lipases (category 26.28) to be repressed in flowers of *FAX1* mutants (Figure S8, Figure S9). Further, lipid transfer proteins (category 26.21) in flowers of *FAX1* over-expressors were down-regulated but up-regulated in stems of *fax1* knockouts (compare Table S5, Table S6, Figure S9, Figure S10). In flowers of *FAX1* over-expressors (Figure S9) three other spotlights for regulation of gene expression could be found: hormone metabolism concerning abscisic acid, auxin, brassinosteroids, and giberellin (17.1, 17.2, 17.3, 17.6) as well as protein- and signaling-associated processes. For the protein category, interestingly genes for amino acid activation (29.1) and protein synthesis (29.2.11, 29.2.2) were induced, whereas genes for protein degradation (29.5.1, 29.5.3/4/5) were mainly repressed. For signaling, we found besides lipid signaling (see above), regulation of calcium signaling genes (30.3) in all three comparisons (Figure S8, Figure S9, Figure S10), and of receptor kinases (30.2) in flowers of *FAX1* over-expressors and *fax1* knockout stems. A strong focus on down-regulation of RALF genes (30.8), coding for peptide growth factors, was only present in flowers of *FAX1* over-expressors.

**References**

1. Li-Beisson Y, Shorrosh B, Beisson F, Andersson MX, Arondel V, et al. (2013) Acyl-lipid metabolism. Arabidopsis Book 11: e0161. Available: http://www.ncbi.nlm.nih.gov/ pubmed/23505340.

2. Pribat A, Sormani R, Rousseau-Gueutin M, Julkowska MM, Testerink C, et al. (2012) A novel class of PTEN protein in Arabidopsis displays unusual phosphoinositide phosphatase activity and efficiently binds phosphatidic acid. Biochem J 441: 161-171.

3. Kim HU, Hsieh K, Ratnayake C, Huang AH (2002) A novel group of oleosins is present inside the pollen of Arabidopsis. J Biol Chem 277: 22677-22684.

4. Greer S, Wen M, Bird D, Wu XM, Samuels L, et al. (2007) The cytochrome p450 enzyme CYP96A15 is the midchain alkane hydroxylase responsible for formation of secondary alcohols and ketones in stem cuticular wax of arabidopsis. Plant Physiol 145: 653-667.

5. Doan TT, Domergue F, Fournier AE, Vishwanath SJ, Rowland O, et al. (2012) Biochemical characterization of a chloroplast localized fatty acid reductase from Arabidopsis thaliana. Biochim Biophys Acta 1821: 1244-1255.

6. Chen W, Yu XH, Zhang K, Shi J, De Oliveira S, et al. (2011) Male Sterile2 encodes a plastid-localized fatty acyl carrier protein reductase required for pollen exine development in Arabidopsis. Plant Physiol 157: 842-853.
