# Supplementary material for: FAX1, a Novel Membrane Protein Mediating Plastid Fatty Acid Export
Source: PLoS Biol. 2015 Feb 3;13(2):e1002053. doi: 10.1371/journal.pbio.1002053 (PMC4344464; doi:10.1371/journal.pbio.1002053)
Supplement: S2 Text — (DOCX) [file pbio.1002053.s020.docx]

**Text S2. The role of FAX1 in male flower tissue**

The exine of the pollen cell wall is built by sporopollenin, made of complex biopolymers such as phenols and FA-derivatives. The tapetum cell layer, which surrounds the locules of anthers, in early stages of pollen development synthesizes and secretes lipid precursors of sporopollenin into the locular fluid. Subsequently, sporopollenin is deposited on the microspore surface and forms the sculptured exine layers. At the end of the bicellular pollen stage, tapetum cells undergo programmed cell death and in parallel provide mainly lipid components for the tryphine pollen coat, a mixture of proteins and lipids that is deposited in the exine cavities (for overview, see [1]). Thus, the FAs for exine and tryphine components are delivered by plastids of the sporophytic tapetum tissue, which is fully degraded when mature, tricellular pollen stages are reached. For FAX1, we therefore propose a function in FA-export from plastids of tapetum cells, which in *fax1* knockouts leads to the strongly impaired assembly of exine layers and pollen coat, due to the absence of FA-precursors for sporopollenin and/or tryphine synthesis. Thereby, we can explain the strong effect of homozygous *fax1* male sporophytes (anthers) observed in genetic analysis. Most likely, the electron-dense, sticky material in *fax1* knockout anthers, which prevents release of pollen grains, represents cellular debris of degenerated tapetum cells and/or not incorporated sporopollenin or tryphine material of non FA-derived precursors, e.g. proteinaceous tryphine compounds or oleosin protein bodies (see [2]). These morphological findings are underlined by differential gene expression in *FAX1* mutant flowers, showing a focus on down-regulation for tapetum-specific oleosin genes (see Table S5). An additional effect on fertility, originating from the male gametophyte (pollen grain) became visible when heterozygous *fax1* anthers were used for pollination (Table 2). Here the absence of the outer pollen cell wall, of course might impair germination and pollen tube growth on the stigma. Further, missing FA-compounds from pollen intrinsic plastids, could contribute to exine and pollen coat assembly or impaired growth of pollen tubes as well.

In the plastid IE membrane, two proteins are described to affect lipid homeostasis in tapetum cells and pollen cell wall formation, which lead to male sterility upon mutation as well: (i) The protein BnMS3 in *Brassica napus*, corresponds to the chloroplast IE protein translocon component TIC40 and is involved in tapetum development, microspore release and pollen wall formation, most likely by transcriptional regulation in mutants and/or by specific protein translocation across the IE [3]. (ii) Disruption of the protein NEF1, predicted as plastid integral membrane protein with 29 α-helical membrane domains, affects lipid accumulation in plastids of tapetum cells and pollen exine formation [4]. Since NEF1 contains a prokaryotic membrane lipoprotein lipid attachment site a function in maintenance of envelope integrity is suggested.

**References**

1. Ariizumi T, Toriyama K (2011) Genetic Regulation of Sporopollenin Synthesis and Pollen Exine Development. Annu. Rev. Plant Biol. 62: 437-460.

2. Hsieh K, Huang AH (2004) Endoplasmic reticulum, oleosins, and oils in seeds and tapetum cells. Plant Physiol 136: 3427-3434.

3. Zhou Z, Dun X, Xia S, Shi D, Qin M, et al. (2012) BnMs3 is required for tapetal differentiation and degradation, microspore separation, and pollen-wall biosynthesis in Brassica napus. J Exp Bot 63: 2041-2058.

4. Ariizumi T, Hatakeyama K, Hinata K, Inatsugi R, Nishida I, et al. (2004) Disruption of the novel plant protein NEF1 affects lipid accumulation in the plastids of the tapetum and exine formation of pollen, resulting in male sterility in Arabidopsis thaliana. Plant J 39: 170-181.
